# The evolution of vertebrate tetraspanins: gene loss, retention, and massive positive selection after whole genome duplications

**DOI:** 10.1186/1471-2148-10-306

**Published:** 2010-10-13

**Authors:** Shengfeng Huang, Haozhen Tian, Zelin Chen, Ting Yu, Anlong Xu

**Affiliations:** 1 State Key Laboratory of Biocontrol, Guangdong Key Laboratory of Pharmaceutical Functional Genes, College of Life Sciences, Sun Yat-sen University, Guangzhou, 510275, China

## Abstract

**Background:**

The vertebrate tetraspanin family has many features which make it suitable for preserving the imprint of ancient sequence evolution and amenable for phylogenomic analysis. So we believe that an in-depth analysis of the tetraspanin evolution not only provides more complete understanding of tetraspanin biology, but offers new insights into the influence of the two rounds of whole genome duplication (2R-WGD) at the origin of vertebrates.

**Results:**

A detailed phylogeny of vertebrate tetraspanins was constructed by using multiple lines of information, including sequence-based phylogenetics, key structural features, intron configuration and genomic synteny. In particular, a total of 38 modern tetraspanin ortholog lineages in bony vertebrates have been identified and subsequently classified into 17 ancestral lineages existing before 2R-WGD. Based on this phylogeny, we found that the ohnolog retention rate of tetraspanins after 2R-WGD was three times as the average (a rate similar to those of transcription factors and protein kinases). This high rate didn't increase the tetrapanin family size, but changed the family composition, possibly by displacing vertebrate-specific gene lineages with the lineages conserved across deuterostomes. We also found that the period from 2R-WGD to recent time is controlled by gene losses. Meanwhile, positive selection has been detected on 80% of the branches right after 2R-WGDs, which declines significantly on both magnitude and extensity on the following speciation branches. Notably, the loss of mammalian RDS2 is accompanied by strong positive selection on mammalian ROM1, possibly due to gene loss-induced compensatory evolution.

**Conclusions:**

First, different from transcription factors and kinases, high duplicate retention rate after 2R-WGD didn't increase the tetraspanin family size but just reshaped the family composition. Second, the evolution of tetraspanins right after 2R-WGD had been impacted by a massive wave of gene loss and positive selection on coding sequences. Third, the lingering effect of 2R-WGD on tetraspanin gene loss and positive selection might last for 300-400 million years.

## 1 Background

**Figure 1:**
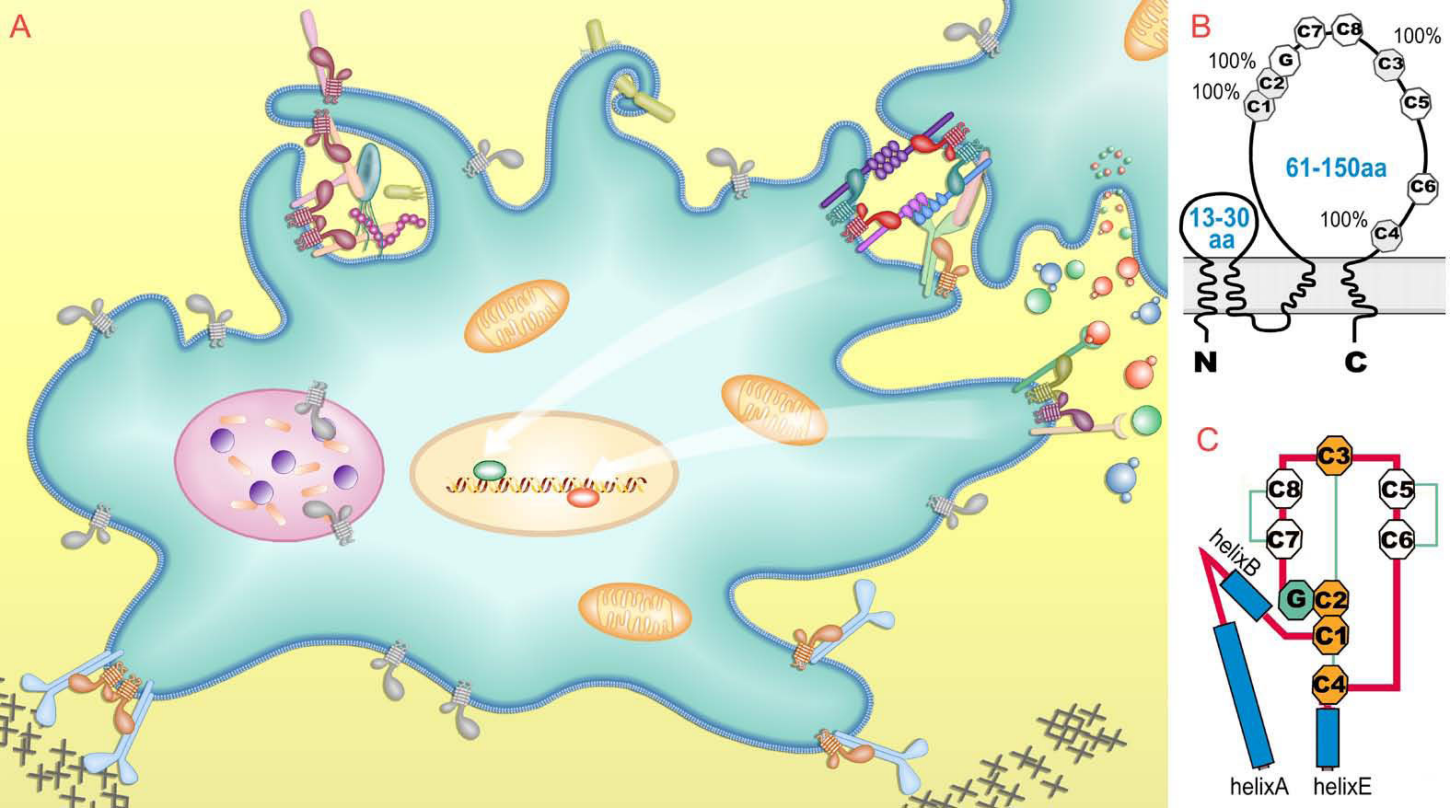
**The functions and structures of vertebrate tetraspanins**. (A) Schematic of some tetraspanin functions. (B) The tetraspanin overall structure. C1~C8 indicate the conserved cysteines; 100% conserved cysteines are labeled; 'CCG' is the so-called tetraspanin signature. (C) The LEL structure of tetraspanins.

Tetraspanin genes are virtually expressed on all cell types in animals [1], and in green plants, fungi and amoebas [2]. They have roles in the immune system, the nervous system, tumor, development, infection and fertilization [3-9]. They mediate various biological processes including cell morphology, adhesion, motility, proliferation, differentiation, fusion, invasion, phagocytosis, exocytosis, receptor signaling, synapse formation, protein trafficking, and more [3-9]. Though other mechanisms do exist, tetraspanins are most found to function as organizers of various kinds of tetraspanin-enriched membrane microdomains (TEMs) [10-14]. As organizers, tetraspanins bind with primary partner proteins and dimerize with each other to form "tetraspanin webs", which further recruit a variety of proteins to form specialized functional complexes (Figure 1A). Despite the biological importance and many advances achieved in last two decades, most tetraspanins have not been functionally explored because of their subtle and overlapping roles [15]. Tetraspanin proteins are 200~350aa long, containing four transmembrance domains (TM), one small extracellular loop (SEL) and one large extracellular loop (LEL) (Figure 1B) [16-18]. LEL contains most of the sites for protein interactions [16-18], and is composed of a constant region and a variable region. The constant region is formed by three helices, whereas the variable region contains a CCG motif, two to four internal disulphide bonds, and some other motifs (Figure 1C) [16-18]. Because of incremental analyses from several early reports, the origin and basic phylogenetic features of tetraspanins are quite clear now [2,19-23].

Gene duplication is a major force for gene functional innovation [24]. However, most duplicate genes are doomed to loss, but gene retention may be facilitated by expression divergence, neofunctionalization or subfunctionalization [25-27]. Small-scale duplication (SSD) and whole genome duplication (WGD) differ in their influence on the fate of duplicate genes [28,29]. For example, in yeasts and plants, duplicate genes from functional classes like transcription factors and kinases tend to be preferentially retained after WGD; also in yeasts, duplicate genes retained after WGD tend to diverge more quickly in expression regulation than in protein function [reviewed in [30]]. In vertebrates, two rounds of WGD (2R-WGD) happened at the origin of vertebrates, and a third round occurred specifically to teleost fishes (FSGD). Early genome-wide studies suggest that no increase of positive selection has been detected on coding sequences of retained duplicate genes right after FSGD [31,32]. The same conclusion is also suggested to be applicable for 2R-WGD [32]. Consistently, several studies on individual gene families find little positive selection right after 2R-WGD, although these studies are not specifically designed to address this issue [33-37]. These reports suggest that the rapid increase of biological complexity and novelties at the origin of vertebrates [38,39], which is believed to be triggered by 2R-WGD, might not be initially attained through positive selection. However, we have reasons to suspect that 2R-WGD's effect on positive selection could be underestimated. First, the effect has not been carefully evaluated actually; second, since 2R-WGD is so ancient (450-500 Mya), even a massive positive selection happened, not every modern genes may still carry the selection imprint after such a long time.

The vertebrate tetraspanin superfamily has several advantages which make it suitable for preserving the imprint of ancient sequence evolution and amenable for phylogenomic analysis. It is a large family with 38 modern ortholog lineages in bony vertebrates (see Results). It has a rigid and compartmentalized protein architecture, which makes it free from domain reshuffling and internal module duplication, or in other words, which limits its structural evolution to amino acid substitutions, insertions and deletions. It maintains highly conserved coding sequences, structural motifs and intron configurations within sub-families. It has high gene retention rate and most of its members preserve traceable genomic syntenic relationships (see Results). Finally, its molecular function is to act as versatile organizers by interacting with various proteins at different affinities, and most of its members serve important but subtle, overlapping and non-essential functions. These biochemical properties make tetraspanins highly adaptable for functional changes.

In this study, we first reconstructed a detailed phylogeny for bony vertebrate tetraspanins by integrating multiple lines of information from sequence-based phylogenetics, key structural features, intron configuration and genomic synteny. Based on this phylogeny, we evaluated the impact of 2R-WGD on the following gene loss, retention and natural selection of the major lineages of vertebrate tetraspanins. In terms of selection tests, we used a stringent branch-site codon model, and contrasted duplication branches with speciation branches. Finally, we concluded that vertebrate tetraspanins had undergone a massive wave of gene losses and positive selection on coding sequences right after 2R-WGD.

## 2 Results

### 2.1 Tetraspanin genes of deuterostomes

To evaluate the 2R-WGD effect on bony vertebrate genes, we require three aspects of phylogenomic information, including the closest invertebrate (outgroup) orthologs, ohnolog patterns, and molecular gene trees with dense taxa. Since we concentrate on bony vertebrates and most tetraspanin lineages are phylum-specific [2], this study is focused on deuterostome tetraspanins. To determine the major phylogenetic structure of vertebrate tetraspanins, we curated a reference set of tetraspanins from the genomes of human (33 genes), mouse (32), zebrafish (50), ascidian *C. intestinalis *(33), amphioxus *B. floridae *(39) and echinoderm *S. purpurastus *(29) (see Methods). Four molecular protein trees have been constructed from these reference sequences using ME, MP and ML methods (Additional file 1, Figure S1-4). To our knowledge, these reference sequences define the most complete tetraspanin repertoires from these species thus far (Additional file 2). Since the protein sequences between sub-families are so divergent (p-distance between many sub-families ranging from 0.75 to 0.9), these trees provide little reliable consensus on the relation between major sub-families. However, together with the information from previous reports [2,21,22], these trees clearly define 38 ortholog lineages for modern bony vertebrates (discussed later).

### 2.2 Phylogenetics of deuterostome invertebrate tetraspanins

**Figure 2:**
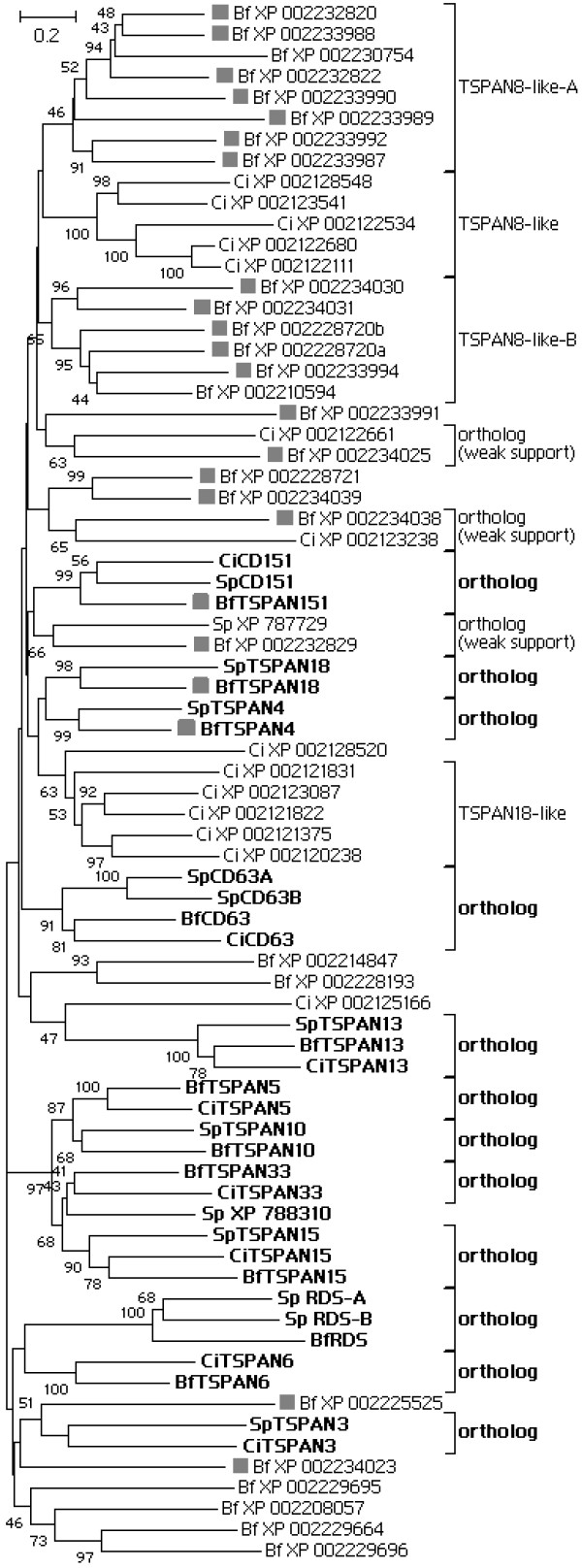
**The protein phylogenetic (ME) tree of invertebrate deuterostome tetraspanins**. Proteins marked with a rectangle are members of the TSPAN4 gene cluster. This tree includes all 39 amphioxus sequences but omitted 9 *C. instestinalis *sequences and 5 *S. purpuratus*. Readers interested in the omitted genes are referred to Figure S1-4.

Assuming that the phylogenetic structure of invertebrate tetraspanins may be distorted by both the large number of species-specific genes and the shift of the evolutionary pattern after 2R-WGD, here we first evaluated invertebrate deuterostome tetraspanins. Fifteen ortholog lineages have been identified from the phylogenetic tree (Figure 2). Twelve of them have vertebrate orthologs and hence represent the lineages conserved across deuterostomes; three of them, with less statistic support, have no vertebrate orthologs (probably lost). Amphioxus tetraspanins are found on fourteen lineages, whereas *C. intestinalis *and *S. purpuratus *each have tetraspanins on ten lineages. Remarkably, amphioxus orthologs on these lineages remain single copy status, suggesting that their gene duplicability might be suppressed by gene dosage effect [see review [30] for the theory]. In contrast to this, amphioxus developed 25 species-specific tetraspanins. Such species-specific gene expansion also occurred in *C. intestinalis *and *S. purpuratus*. So species-specific SSDs and rapid divergence are major force to shape the tetraspanin repertoires in deuterostome invertebrates, which is consistent with the situation in other invertebrates like *C. elegans *and *D. melanogaster *[2].

### 2.3 A large tetraspanin gene cluster in amphioxus

**Figure 3:**
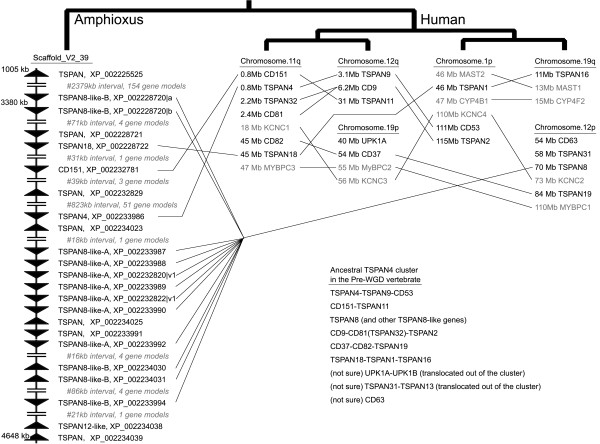
**The chordate TSPAN4 gene clusters in amphioxus and humans**. Triangles show the gene direction. Picture is not drawn to scale. The relationships between four human paralogous region are shown using a tree pattern. An inferred TSPAN4 cluster in the pre-WGD vertebrate ancestor is also provided.

A large tetraspanin cluster is present in the genome of *D. melanogaster*, accounting for half of the 35 *D. melanogaster *tetraspanin genes [20]. Here we reported a tetraspanin cluster (designated the TSPAN4 cluster) in the amphioxus genome. It is located on the scaffold_V2_39 and spans 3600 kb, containing 23 tetraspanin genes and 223 non-tetraspanin genes (Figure 2, 3). If the 5-most member (XP_002225525) is excluded, it still spans 1300 kb and contains 69 non-tetraspanin genes. The TSPAN4 cluster includes TSPAN4, TSPAN18, CD151, seven TSPAN8-like-A, five TSPAN8-like-B and eight other tetraspanins. Given the existence of its orthologous regions in *C. intestinalis *(data not shown) and in vertebrates (Figure 3), the origin of this cluster should be dated back to 500~600 Mya. This cluster offers rich information for tracing the evolutionary history of vertebrate tetraspanins.

### 2.4 Phylogenomic analysis of vertebrate tetraspanins

We compared different molecular trees of reference tetraspanins, and identified 38 bony vertebrate tetraspanin ortholog lineages (Additional file 1, Figure S1-4). We also expanded the analysis to include more than 23 genomes to make sure no more lineages found (see Methods). Notably, here we required an ortholog lineage to contain representatives from at least two bony vertebrate classes among mammals, reptiles, amphibians and teleost fishes. Further comparison between this analysis and that of deuterosome invertebrate tetraspanins helped to classify 38 ortholog lineages into 17 ancient super-lineages which were originated before 2R-WGD. Because sequence divergence within ancient lineages was sometimes large and the sequence-based inference of some ancient lineages were statistically less reliable, we confirmed each of these ancient lineages by examining their critical structural features (Table 1), intron configurations (like [22]) and genomic synteny. Notably, these information provided support for some evolutionary relations between ancient lineages with certain reliability (discussed later).

**Table 1:** Classification of all vertebrate tetraspanin LELs

subtype	cysteine pattern of LEL	tetraspanin families
4-cys	CCG--C--C	CD81
6-cys-a	CCG--[DN][WY]--PXXCC--C--GC	CD151, CD63, TSPAN3, TSPAN6, TSPAN12, TSPAN18, RDS, UPK1, TSPAN4(the member CD53 secondarily lost cysteine No.4&5)
6-cys-b1	CCG--[DN][WY]--[P]XXCXC--C--GC	TSPAN8, some amphioxus and ascidian TSPAN8-like
6-cys-b2	CCG--[DN][WY]--PCXC--C--GC	CD37
6-cys-c	types other than 6a, 6b1, 6b2	TSPAN13, some amphioxus TSPAN8-like
8-cys	CCG--[DN][WY]--C--C--PXXCC--C--GC	TSPAN5, TSPAN10, TSPAN15 and TSPAN33

It is worth noting that phylogenetic structures inferred in this study provides more complete information than our previous work [2], and can be quite different from Garcia-Espana et al's work [21]. For the latter case we figured out three reasons. First, more genome sequences and more complete gene data set have been included; second, multiple tree reconstruction methods including more reliable methods like maximum-likelihood (ML) and Bayesian inference were used (see Methods); third, structural motifs, intron configurations and genomic synteny were integrated into the analyses in addition to sequence-based phylogenetics.

### 2.5 Seventeen ancestral vertebrate tetraspanin lineages

**Figure 4:**
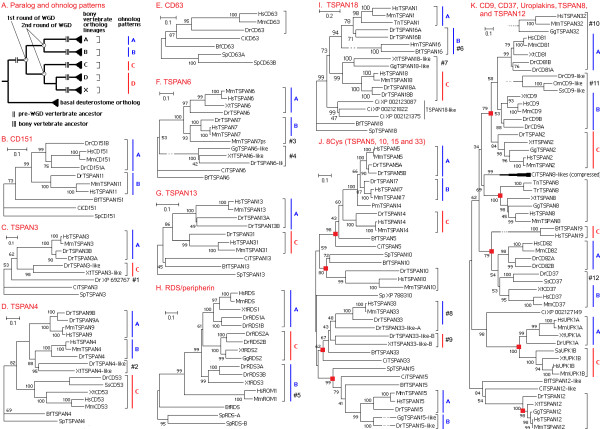
**The protein phylogenetic (ME) trees of all 17 ancestral (pre-WGD) vertebrate tetraspanin lineages**. The bony vertebrate ortholog lineages and the ohnolog patterns are highlighted as shown in panel A. Red-filled rectangles are used to mark each ancestral lineage in panel J and K. #1, a distant tetraspanin, only found in ray-finned fish *otocephala*; #2, found in ray-finned fishes, xenopus and reptiles, probably an independent duplicate of TSPAN4; #3, a tetraspanin pseudogene from mouse; #4, a divergent tetraspanin found in all bony vertebrates, originated by retrotransposition; #5, mammalian ROM1, the true ortholog of bony vertebrate ROM1, but too divergent to cluster with other ROM1; #6, mammal TSPAN16, the true ortholog of teleost TSPAN16, but too divergent to cluster with other TSPAN16; #7, a divergent lineage, its position is not determined; #8 and #9, no synteny shared between TSPAN33 and TSPAN33-like, but since they were separated before the radiation of bony vertebrates, here we treated them as an ohnolog pair; #10, found in reptiles and mammals, as a result of tandem duplication of CD81, becoming too divergent to cluster with CD81; #11, only found in ray-finned fish *otocephala*, as a result of independent duplication of CD9, becoming too divergent to cluster with CD9; #12, has weak support for its clustering with tetrapod CD37 and sharing no synteny, so its identity is questionable.

The above phylogenomic analysis suggests that 17 pre-2R-WGD ancestral vertebrate tetraspanin genes survived until now. One may envisage that through duplications and speciation, these ancient genes expanded into 17 tetraspanin lineages (subfamilies), which further gave rise to all 38 modern vertebrate tetraspanin sub-lineages. Twelve ancient lineages have orthologs in invertebrate deuterostomes, including CD63, CD151, TSPAN3, TSPAN4, TSPAN6, TSPAN13, RDS/peripherin, TSPAN5, TSPAN10, TSPAN15, TSPAN18 and TSPAN33. Notably, three of these twelve lineages have reliable protostome orthologs, including CD63 (e.g. mosquito XM_311807), CD151 (e.g. mosquito XM_315290) and TSPAN13 (e.g. fruit fly NM_079800). The other five lineages, including CD9, CD37, uroplakins, TSPAN8 and TSPAN12, either have no invertebrate orthologs at all or have distant homologs but have been disproved as orthologs. All 17 ancient lineages and 38 sub-lineages are shown in separate distance trees in Figure 4, which is aimed to show major topologies and the events of long-branch attractions (e.g. the mammalian ROM1). For reliable gene trees with dense taxa (31 taxa used, see Methods), readers are referred to the reference trees used for selection tests (Additional file 1, Figure S5-21), and to the Bayesian protein trees which better contain long-branch effect (Additional file 1, Figure S37-53). Finally, more details on these lineages are presented in the legend of Figure 4; and the supporting data for the ancient lineages that are most difficult to be defined are presented in the Supplemental Text.

### 2.6 The monophyletic 8-cysteine super-lineage and the vertebrate TSPAN4 gene cluster

Although the inferred phylogenetic relations between 17 ancient lineages are much less supported, some reliable parts are nevertheless worth noting. Four 8-Cysteine-containing ancient lineages (TSPAN5, TSPAN10, TSPAN15 and TSPAN33), including eight ortholog sub-lineages, form a monophyletic super-lineage (Figure 4). This super-lineage has a special LEL structure defined by eight cysteines capable of forming four disulphite bonds. In fact, all metaozoan 8-Cys tetraspanins belong to this super-lineage with the oldest one from cnidarians [2], reflecting the ancient origin and independent evolution of this super-lineage. On the other hand, syntenic analysis indicated that the TSPAN4 cluster not only exists in amphioxus and ascidians, but also exists in bony vertebrates, except that in bony vertebrates the cluster has been quadrupled by 2R-WGD. Figure 3 shows the reconstructed pre-WGD status of the vertebrate TSPAN4 cluster. This gene cluster provides insights into the evolution of six ancestral lineages. Four lineages (CD151, TSPAN4, TSPAN18 and TSPAN8-like) should represent the earliest members of this cluster, whereas two other lineages (CD9 and CD37) should be derived from ancestral TSPAN8-like genes given structural similarity between them. Besides, the origin of uroplakin, CD63 and TSPAN13 seem to be related to this cluster. Lastly, more evidence and discussion on the evolution of these tetraspanins are presented in the text of additional file 1.

### 2.7 The ohnolog duplication patterns produced by 2R-WGD

The 2R-WGD has been confirmed by lines of convincing evidence [40-42]. Apparently due to WGDs, bony vertebrates have many more genes than invertebrates [39]. Paralogs arisen from WGD are called "ohnologs" [43]. Ideally, four ohnologs produced by two consecutive WGDs manifest a hierarchical pattern like ((A, B),(C, D)), but the pattern recovered from phylogenetic analysis is often violated by gene losses and unequal evolutionary pace in different duplicates. Apparently, all 38 tetraspanin lineages of bony vertebrates are paralogs derived from 17 pre-2R-WGD lineages, but not all paralogs from within an ancient lineage are ohnologs produced by 2R-WGD. In other words, some paralogs could be paralogs originated through SSDs. Here we used phylogenetic trees and syntenic data to determine SSD-derived paralogs, 2R-WGD-derived ohnologs and the ohnolog duplication patterns (Figure 4) (see Methods). More details on these patterns are presented in the legend of Figure 4.

### 2.8 Gene retentions and losses after 2R-WGD

Among 17 ancestral lineages, 13 retain two or more ohnologs and 6 of 13 retain three ohnologs. These 13 lineages include CD151, TSPAN3, TSPAN4, TSPAN6, TSPAN13, RDS/peripherin, TSPAN18, TSPAN5, TSPAN15, TSPAN33, CD9, CD37 and uroplakin, accounting for 32 of 38 tetrapanin lineages in bony vertebrates (Figure 4). This ohnolog retention rate is quite high, approximately three times the average rate (average rate is ~25%, see [42]) of all bony vertebrate genes, which is similar to the retention rate of transcription factors and protein kinases. In summary, of 21 new tetraspanin lineages in bony vertebrates (38-17 = 21), 19 are contributed by 2R-WGD and only 2 by SSDs (Figure 4). This contrasts the invertebrate situation, where SSDs control the family size tetraspanins (see Section 2.2). Remarkably, although the high ohnolog retention rate greatly increased the family sizes of vertebrate transcription factors and kinases [42,44], it didn't increase the family size of bony vertebrate tetraspanins (for comparison: 33 members in human, 32 in mouse, 28 in chicken, 33 in *C. intestinalis*, 39 in *B. floridae*, 29 in *S. purpurastus*, 35 in *D. melanogaster *and 20 in *C. elegans*). Taken together, 2R-WGD and the high ohnolog retention rate reshape the tetraspanin family composition rather than increase the tetraspanin family size.

Although 75% (13 of 17) of the ancient tetraspanin lineages have ohnologous duplicates retained in bony vertebrates, nearly half (17*4-36 = 32) of the duplicates produced by 2R-WGD were lost before the radiation of bony vertebrates, not mentioning some lineages which might be lost without trace left. In contrast to this, meanwhile only two new lineages were acquired through SSDs. Gene losses are also observed to continue prevailing over gene gains in later evolution of bony vertebrates. For example, human lost six of 38 tetraspanin ortholog lineages and gained only one new gene (TSPAN32) through SSD; mouse lost eight and gained one (TSPAN32, excluding the TSPAN7-like pseudogene); chicken gained two new tetraspanins but probably lost twelve; amphibians probably lost three but gained one; zebrafish gained two through SSDs (pseudogene XP_692767 excluded), but even FSGD could not prevent it from losing four bony vertebrate tetraspanin lineages. So we concluded that it is gene losses but not gene gains which prevail in the evolutionary history of bony vertebrate tetraspanins since 2R-WGD.

### 2.9 Massive and extensive positive selection right after WGD

**Figure 5:**
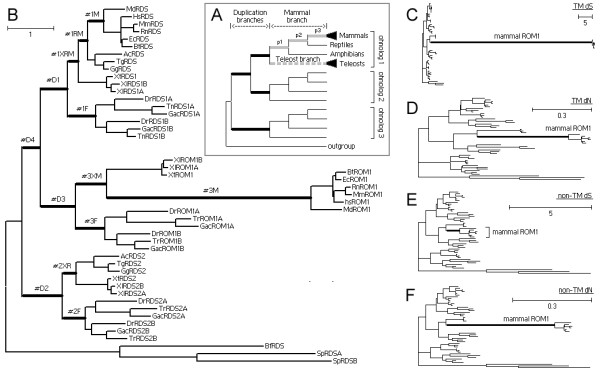
**(A) Schematic representation of the tree topology**. Three types of branches (in thick lines) are selected for branch-site model tests for positive selection. (B) The one-ratio tree of RDS. The branches selected for tests are marked with "#". (C-F) RDS gene trees inferred using branch-specific models, with the mammalian ROM1 branch as the foreground branch. (C) Synonymous substitution (d_S_) tree of transmembrane region (TM). (D) Nonsynonymous substitution (d_N_) tree of TMs. (E) d_S _tree of the non-TM regions. (F) d_N _tree of the non-TM region.

We divided branches following 2R-WGD into two conceptual categories, duplication branches and speciation branches (Figure 5A). Contrasting duplication branches with speciation branches permits to detect the changes in selective regimes on two categories of branches. The major difference between duplication branches and speciation branches is their divergent time from the first WGD. Here we used a stringent branch-site test to detect positive selection (see Methods). Three types of branches were examined, including duplication branches, speciation branches leading to mammals, and speciation branches leading to the teleost fishes (Figure 5A).

Several factors that complicate the analysis of deep evolution of vertebrates need to be assessed before performing selection tests. A large tree with many branches will improve the power of likelihood tests when sequence divergence is high [45]. We sampled 24 fully-sequenced genomes and many sequences from other sources. Several mammalian genomes were excluded due to poor quality. Excessive mammalian species won't enhance the power and robustness of our tests because they don't break long internal branches and the real bottle neck in our tests is the lack of sequences from reptiles and amphibians. Any way, we compiled at least five mammal species, four teleost species and as many species as possible for reptiles and amphibians. So, to our knowledge, in most cases the taxon number used in our tests has exceeded early studies [31-37]. The structures of the reference trees used for selection tests have been justified by comparing information from Bayesian protein trees, Maximum likelihood trees, distance trees, and syntenic data. So, one may notice that the reference trees (Additional file 1, Figure S5-21) and the Bayesian protein trees (Additional file 2, Figure S37-53) have some discrepancies. In fact, selection tests using both types of trees delivered consistent results (data not shown), which is expected because the difference between two trees is caused by the lack of sufficient substitution  information on the related branches and the lack of such information is unlikely to produce false positives. Highly divergent branches were removed before tests (but will be separately analyzed in the next section). Since we are testing multiple branches in one tree, a scheme of multiple-testing correction is required: first, because the evolution of duplicated genes may affect with each other right after WGD, all duplication branches in a tree are treated as one hypothesis; second, since the branch leading to the mammal class has been divided into three portions by amphibians and reptiles, all three portions of the branch are also treated as one hypothesis; third, as for the branch leading to the teleost class, it forms one hypothesis alone (Figure 5A). This scheme is expected to be conservative for duplication branches and relaxed for mammal branches. Hocheberg's method is used here (5% and 1%) [46], which is more stringent than FDR method use in [32] (10% and 1%). As for the distributions of codon usage, GC content and transition-transversion ratio, we analyzed them in three layers. First, within the vertebrate ortholog lineages, the difference could be presented between vertebrate classes. but these differences unlikely affect the accuracy and power of likelihood ratio tests (LRT) on branches inside the lineage according to the previous simulations [32]. Second, between ohnolog lineages we detected no significant difference in most cases, which was expected because different lineages contained similar composition of species. Third, there was significant difference between invertebrate and vertebrate genes. Though we had not to test invertebrate branches, we performed tests using trees without invertebrates and confirmed that similar results could be obtained with or without invertebrate sequences (data not shown). The last complicating factor we need to consider is the possible dS saturation and model violation on divergent deep branches (duplication branches). Saturation of dS may not be a problem for PAML's likelihood tests [32,45,47], but which is often accompanied by model violation and hence may cause false positives [47]. In the next section, we will show that these problems have little impact on our conclusions.

We have tested all 17 tetraspanin trees (Test No.1-383 in Additional file 3) by using a stringent branch-site model (see Methods), but only 15 were put into further statistic analysis (Table 2). Two trees (TSPAN8 and TSPAN12) were excluded from tests because they lacked proper outgroups and duplication branches. We detected positive selection on 28 of all 36 duplication branches (P < 0.05), which consisted of 12 of 15 trees. In contrast, we detected positive selection on 16 of 33 mammal branches and 16 of 30 teleost branches. So, the difference between duplication branches and speciation branches is statistically significant. This difference becomes even more significant if the P-value for likelihood tests was lifted to 0.01, suggesting that positive selection on duplication branches is not only more prevalent but also stronger than that of speciation branches. In consistence, positive selection affects 6~36% percent of sites (average 18%) on the duplication branches, contrasting to 0.6~23% (average 7%) on the mammal branches and 0.9~35% (average 9.6%) on the teleost branches. Typically, positive selection on many tested speciation branches was contributed by only 1-3 sites (Additional file 3). This result was also confirmed by the proportion of the sites predicted to be under positive selection by the Bayes empirical Bayes (BEB) method (Additional file 3). Furthermore, if we considered the post-WGD evolution towards mammal radiation as a four-staged process (before the bony vertebrate radiation, before the tetrapod radiation, before the reptile-mammal radiation and before the mammal radiation, see Figure 5A), we could observe a gradual decline on the occurrence of positive selection from 80% to 36%, to 24% and to 12% (the difference in last three stages is insignificant due to small sample size). Taken together, we concluded that for vertebrate tetraspanin genes, massive and extensive positive selection dominated the early period after 2R-WGD, which gradually and significantly decreased in both magnitude and extensity in later bony vertebrate evolution.

**Table 2:** Statistics on branches under significant positive selection

Branch type	No. of branches	Proportion of sites under positive selection average(median)	Significant LRT^a ^(P < 0.05) (proportion)	Fisher's exact test^b^	Significant LRT^a ^(P < 0.01) (proportion)	Fisher's exact test^b^
* *13 tested trees, CD63, TSPAN8, TSPAN10 and TSPAN12 excluded*						
A. duplications	34	-	26(76%)	0.0425;A/B	19(56%)	0.0045;A/B
B. mammals^c ^(portion 1,2,3 as a whole)	31	-	15(48%)	0.7961;B/D	6(19%)	0.7552;B/D
C. mammals^c ^(portion 1,2,3 as independent branches)	81	-	26(32%)	1.4e-5;A/C	13(16%)	3.0e-5;A/C
D. teleosts	28	-	15(54%)	0.0667;A/D	7(25%)	0.0202;A/D
* *15 tested trees, TSPAN8 and TSPAN12 excluded*						
E. duplications	36	18.4%(19.0%)	28(78%)	0.0138;E/F	20(56%)	0.0025;E/F
F. mammals^c ^(portion 1,2,3 as a whole)	33	-	16(48%)	0.8025;F/H	6(18%)	0.7577;F/H
G. mammals^c ^(portion 1,2,3 as independent branches)	86	7.2%(4.1%)	27(31%)	3.0e-6;E/G	14(16%)	0.0001;E/G
H. teleosts	30	9.6%(8.3%)	16(53%)	0.0652;E/H	7(23%)	0.0118;E/H
* *TSPAN8 and TSPAN12* *(Though the branch-site model detects no positive selection, the site-specific model detects positive selection on TSPAN8)*						
I. duplications	0	-	-	-	-	-
J. speciation (as independent branches)	6	-	0(0%)	-	0(0%)	-

^a^Number of branches on which positive selection is detected using branch-site model at given p-value; Hochberg's method (1988) was used for multiple testing correction.

^b^Fisher's exact test was used for testing the difference between different types of branches

^c^The bony vertebrate-mammal branch was interrupted by amphibians and reptiles, hence produced portion 1,2,3, see Figure 5A for details.

### 2.10 Further confirmation of the massive wave of positive selection after 2R-WGD

Since the statistic conclusion from last section is so different from those early studies using similar detection schemes for positive selection on vertebrate gene lineages [31-37] (also see the Introduction), we decided to thoroughly evaluate its robustness from multiple angles. In our tests, we ignored two trees (TSPAN8 and TSPAN12) due to the lack of proper outgroups and took into account two trees (CD63 and TSPAN10) that have fallen back to single gene status, but in fact, whether to count in these trees or not would not change our statistic conclusions (Additional file 3). To further verify the reliability of inference, we performed tests on twelve arbitrarily selected duplication branches using the F61 codon model (CodonFreq = 3 in PAML4), which is expected to contain the bias on GC content, codon usage and transition-transversion ratio better than the F3X4 model. Both models produced similar results, except that the F61 model caused a bit of power loss in LRT. Notably, the quality of sequences can affect the parameter estimation, especially when genomes are sequenced and assembled in varying quality [for example [48]], but after analyzing the branch-site likelihood tests, we figured out that sequence errors tend to increase more false positives on the terminal speciation branches than the deep internal duplication branches. In fact, since in this study the positive selection on duplication branches was much stronger and affected much more sites than on speciation branches, we expect that no matter what testing schemes or sequence problems are encountered, the duplication branches should be less affected than speciation branches.

Two factors, the dS saturation and the violation of the codon model assumption, will inflate false positive rates of likelihood ratio tests for positive selection [47,49]. In our tetraspanin dataset, duplication branches produced by 2R-WGD are so ancient and divergent that they have much higher chance to suffer from saturation and model violation than speciation branches. Therefore, we have designed three schemes to confirm that the excessive positive selection detected on tetraspanins' duplication branches is evident and trust-worthy.

First, we ran simulations that reproduced the original dataset under nearly-neutral model to evaluate the false positive rates of positive selection on duplication branches (see Methods). We detected no positive selection (P < 0.05) on all 170 tested branches. To correction for the possible underestimation of dS substitution rate, we ran another simulation with branch length multiplied by 1.5. This time we detected positive selection (P < 0.05) on only 2 branches among all 170 tested branches. So, it is clear that dS saturation-induced false positive rate is not a big problem, which is consistent with early simulation analyses [32,45,47].

Second, we especially assessed those divergent branches in our tetraspanin dataset. There are four duplication branches with dS > 1.8, two of which are reported under positive selection (TSPAN14 and TSPAN15-like). Positive selection on the TSPAN14 branch is highly supported (P < 0.00002) and affects a large proportion of sites (48%), whereas dS of the TSPAN15-like branch is merely above 1.8 (≈1.87) and positive selection on it is also highly supported (P < 0.002). Moreover, after we removed branches with dS > 1.5 (15 branches) or dS > 1.2 (23 branches), our statistic conclusions still hold, suggesting that in our tetraspanin dataset, divergent branches did not show any higher false positive rate than those less divergent.

Third, we employed two protein-based approaches to detect evolutionary rate shifts on or between tetraspanin ohnolog lineages (implemented in software DIVERGE2 [50] and RASER2 [51], see Methods for details). It is suggested that in theory amino acid-based methods for selection tests may better contain divergent sequences and dS saturation than codon-based methods [45]. Using DIVERGE2, we detected significant functional divergence between all pairs of tetraspanin ohnolog lineages except the pair of TSPAN6/TSPAN7 (Additional file 4). It should be noted that there were also no positive selection detected on TSPAN6 and TSPAN7 using the branch-site codon model (Additional file 3). Using RASER2, we detected significant rate shifts on all tetraspanin ohnolog lineages (Additional file 5). Globally, protein-based methods produced much higher significance on each ohnolog lineages than codon-based methods (readers can compare Additional files 3-5 to see this point). Taken together, we concluded that: 1) protein-based methods seem less stringent than codon-based methods; 2) significant rate shifts did happen on the protein level on tetraspanin ohnolog lineages (corresponding to duplication branches), which strongly corroborates the results from the branch-site codon model.

### 2.11 Natural selection on the extremely long branches

The most divergent branches were not analyzed in Section 2.9, which include four branches originated by independent SSD (TSPAN4-like, TSPAN6-like, TSPAN32-like, CD9-like), two branches arisen from speciation (mammalian ROM1 and TSPAN16), and one branch produced by WGD (TSPAN19). We set up new tests on these branches by using several different models and phylogenetic contexts, but no positive selection could be detected on these branches over the full-length sequence. This outcome is not surprising because codon-based positive-selection models have been shown to have little power in dealing with highly divergent sequences [47]. However, we observed that the evolutionary rate, transition-transversion ratio and d_N_/d_S _ratio could be different between the TM and the non-TM regions in some tetraspanin lineages (data not shown), so we redid tests on these branches with the TM and non-TM regions separated. This time, we detected positive selection in non-TM regions on two branches, the mammalian ROM1 branch (discussed later) and the CD9-like branch. All these tests are present in Additional file 3 (test No.462-671), and their corresponding reference trees are presented on Figure S22-36 (in Additional file 1).

### 2.12 Possible compensatory evolution of ROM1

The RDS family forks into three ohnolog lineages, RDS, ROM1 and RDS2. RDS is a major organizer for the photoreceptor outer segment (OS) architecture [52]. Defects of RDS may cause blindness. ROM1 forms hetero-tetramers and -octamers with RDS in OS [53]. A study of ROM1 -/- mice indicates that though ROM1 has a role in rod photoreceptor viability and in the regulation of disc morphogenesis, RDS alone is sufficient for both disc and outer segment morphogenesis [54]. Defects of ROM1 may also cause photoreceptor degeneration but the phenotype is milder than that of RDS. As for RDS2, the function has not been explored.

The protein structure of RDS family is highly conserved and free of indels, suggesting strong purifying selection against indels. Positive selection can be detected on all duplication branches and affects 12~29% of the sites, whereas positive selection is much less on speciation branches (Addition file 3). A notable observation about the RDS family is the accelerated substitution rate of the mammalian ROM1 (mROM1) branch (Figure 5B), which is so extreme that it causes aberrant topology in the distance tree (Figure 4H), but the correct topology can be recovered by using ML or Bayesian methods (Figure S3 & S43 in Additional file 1). Analysis of the genes adjacent to the genomic location of mROM1 showed that the acceleration is restricted to mROM1, suggesting that regional effects are not responsible for the phenomenon. Since the high substitution rate of mROM1 produces no indels or poor alignment portions, we suspected that acceleration is directional or under certain constraints. The BEB site prediction method reports many sites under positive selection in non-TM regions on the mROM1 branch without significant LRT support. Prompted by this, we divided the alignment into TM and non-TM regions and re-tested the branch (Test No.384-461 in Additional file 3). The rationale of this scheme is that to increase the proportion of positive selection sites will increase the power of LRT [47]. In this time, positive selection has been significantly detected in non-TM regions, affecting 48% of the sites and having 42 under-positive-selection sites predicted with > 95% probability. The likelihood test of positive selection will become much more significant if we reduced the interference from non-ROM1 sequences and model violations by restricting the tests to the sub-tree of bony vertebrate ROM1 (Test No.462-479 in Additional file 3).

To further find out the relation between the positive selection and the substitution acceleration, we applied the branch-specific model for further tests. These tests indicate that most synonymous substitutions on the mROM1 branch are accumulated in TM regions, whereas the synonymous substitution rate in non-TM regions has no difference from other branches (Figure 5C-D). In contrast, most nonsynonymous replacements on the mROM1 branch occur in non-TM regions (Figure 5E-F). These observations not only explain why positive selection is not significant across the whole sequence, but suggest that massive and rapid positive selection in non-TM regions of the mROM1 branch is driven by the acceleration of global substitution rate. However, it is still hard to comprehend why rapid and massive positive selection suddenly acted on the mROM1 branch. We proposed that it is due to compensatory evolution caused by the loss of mammalian RDS2 (see Discussion).

## 3 Discussion

### 3.1 Distinct influence of 2R-WGD on family size and composition of vertebrate tetraspanins

It is reported that after 2R-WGD, only 25% of the ancestral chordate genes retained more than two ohnologs in modern vertebrates [42], but retention events are not random, instead, related to gene functional modes. For instance, genes with high retention rates are enriched in development, signal transduction and transcription regulation [42]. The ohnolog retention rate of vertebrate tetraspanins is found to be 75%, three times the average and similar to that of transcription factors (TFs) and kinases, suggesting that new tetraspanins tended to be quickly recruited into novel biological processes after 2R-WGD. In line with this, in invertebrates, species-specific SSD-derived tetraspanins also had high retention rate, hence accounting for the majority of invertebrate tetraspanins (see Section 2.2). Why do tetraspanins have high duplicability and retention rate? It is proposed that genes with biochemical features permitting easy adaptation for new functions should have higher duplicability [30]. And genes with secondary functions or properties more likely retain both duplicates and develop new functions from secondary properties [30]. These hypotheses may explain the situation of tetraspanins, because tetraspanins are involved in various biological processes and interacting with various proteins at different affinities [13].

Frequent SSDs dominated the tetraspanin family sizes in invertebrates (see Section 2.2), and 2R-WGD could increase TFs and kinases by 2~4 folds. In contrast, neither 2R-WGD nor SSDs significantly increased the family size of vertebrate tetraspanins. Instead, high ohnolog retention rate just reshaped the family composition. So in these terms, influence of 2R-WGD on tetraspanins is quite different from those genes (TFs and kinases) with high retention rates or those genes with low retention rates. Now the question is: why did this happen? We find that only 5 of 17 ancient vertebrate tetraspanin lineages are vertebrate-specific, contrasting to the invertebrate situation where the majority of tetraspanins are species-specific. If assuming that vertebrate ancestors before 2R-WGD had roughly the same amount of tetraspanins as deuterostome invertebrates had (i.e., 29-39 genes), we may infer that 2R-WGD didn't increase the family size of vertebrate tetraspanins but changed the family composition by displacing those vertebrate-specific lineages with the lineages conserved across the deuterostome phylum. If this inference was true, then how did it happen? It is suggested that conserved genes and essential genes are favorable for duplication if initial gene dosage constraint is not a problem [55-57]. It is also proposed that WGD but not SSD is able to lift dosage constraint because dosage balance is not initially altered by WGD [30]. Based these theories, we speculate that in invertebrates conserved tetraspanins are preferable for duplication but constrained by dosage barrier (which might be especially true in amphioxus, where frequent SSDs kept happening but all conserved tetraspanins remain single gene status). However, in vertebrates, since 2R-WGD lifted the dosage barrier, the duplicates of conserved tetraspanins might be able to displace non-conserved genes quickly.

### 3.2 Post-2R-WGD massive positive selection and its relation to gene retention and gene loss

In addition to the high ohnolog retention rates, we also detected a massive wave of positive selection in bony vertebrate tetraspanins right after 2R-WGD. This wave of positive selection impacted nearly 80% of the duplication branches and affected 18% of their sites on average. Rather than classify this selection wave as a peculiar case on tetraspanins, we argue that it occurred on genome-wide scale but some families like tetraspanins might preserve the signal of positive selection better than others. Positive selection were also detected on later speciation branches, but at much less extensity and magnitude, with many branches affected by 1~3 sites of strong signal. We theorize that such a few sites in most cases may not create entirely new functions, but likely contribute minor adaptation or optimization for the new function acquired in early evolution.

Initial retention of new tetraspanin ohnologs permitted subsequent differential changes on their coding regions. Early studies suggest that initial retention of duplicates after WGD is likely facilitated by dosage balancing selection, expression divergence and subfunctionalization [review in [30]]. In this course, Darwin's natural selection favored those adaptive changes (positive selection) and led to neofunctionalization. Although initial retention opens a time window for positive selection, it doesn't directly accelerate positive selection. Some other events like subfunctionalization and gene loss may facilitate positive selection. Initial subfunctionalization on duplicate genes may relax original selective constraints on coding regions and facilitate neofunctionalization (positive selection) in later sequence evolution [58,59]. As for gene loss, it may also cause positive selection according to the dosage-compensation model. This model predicts that after WGD dosage effect strongly prevent the loss of the duplicated genes encoding interacting proteins in order to maintain the balance of the interaction network (note that tetrapanins are known to form huge "tetraspanin webs" with various proteins), but this model also predicts that once one of the interacting duplicates is lost, the remaining duplicated gene will be positively selected for [60]. Therefore, gene loss may facilitate positive selection by triggering compensatory evolution on the remaining duplicate gene. The positive selection on mammalian ROM1 is possibly due to compensatory evolution triggered by the loss of mammalian RDS2.

Compensatory evolution commonly occurs within a gene, a protein complex or a network [61]. Within a gene, deleterious mutations can be rectified or compensated by other mutations; within a complex or a network, defects of a component may be compensated by changes on other components. However, gene-for-gene compensation is rare. Here we propose a functional vacancy hypothesis to explain gene loss-induced positive selection. This model assumes that close related ohnologs or paralogs have diverged but partially overlapped function. So once an ohnolog is lost, other ohnolog may fill the functional vacancy, but since the replacing ohnolog is not as good as the original, directional (or positive) selective pressure may step in and drive further adaptive changes on the replacing ohnolog. This hypothesis may explain the positive selection on mammalian ROM1. Functional study demonstrates that RDS acquired its present function before the radiation of tetrapods [52]. The branch pattern and length of RDS family suggests that before the mammalian speciation, RDS2 is more divergent from RDS and ROM1, whereas ROM1 is more similar to RDS and could be more dispensable or under stronger subfunctionalization. The dispensability of ROM1 has support from reptiles, where the loss of reptile ROM1 caused no discernable effects on the sequence evolution of reptile RDS and RDS2 (Figure 5B). Taken together, we speculate that the loss of mammalian RDS2 might cause an instant functional vacancy, which may drive the less important ROM1 to evolve adaptive changes to fill the vacancy.

Finally, we infer that 2R-WGD associated gene loss and positive selection could have affected the evolution of vertebrate tetraspanins for 300-400 million years or more. First, the early period right after 2R-WGD is ~100 million years, as the divergence of chordates is 520-680 Mya and that of bony vertebrates is ~420 Mya [62,63]. Second, gene losses continued to prevail over gene gains in the later evolution of bony vertebrates (see Section 2.8), and the number of branches under positive selection declined in a gradual way along the evolutionary path from 2R-WGD to the mammal radiation (see Section 2.9). Both phenomena suggest that after the bony vertebrate radiation the influence of 2R-WGD kept declining but continued to act in later 200~300 million years. This lingering effect of 2R-WGD is not restricted to tetraspanins but likely presented on a genome-wide scale, because early studies indicate that the effect of 2R-WGD on the gene family size distribution is still detectable after the tetrapod radiation [64,65].

### 3.3 The possible differences between 2R-WGD and FSGD

An early study shows that duplicate genes retained after FSGD bias to genes under purifying selection or relaxation of purifying selection [31]. Studer *et al*'s work further suggests that FSGD had no effect on the prevalence of positive selection [32]. These conclusions are quite different from ours on 2R-WGD and tetraspnains (a massive wave of positive selection right after 2R-WGD). However, if disregarding the duplication branches, Studer *et al*'s work and our work are more consistent than different: both studies provide similar estimation of the occurrence of positive selection on the mammal branches (12~20%, P < 0.05, portion 3 only, shown in Figure 5A) and on the bony vertebrate branches (43-50%, P < 0.05 equivalent to the teleost+tetrapod branches, shown in Figure 5A). This implies that the difference lies in duplication branches, in other words, lies between 2R-WGD and FSGD.

One difference between 2R-WGD and FSGD is their differential roles in the increase of biological complexity. 2R-WGD is believed to greatly increase the morphological complexity (including developmental boundaries and gene functional modules) in the transition from basal chordates to vertebrates [38]. However, it appears less dramatic about the changes on morphological complexity before and after FSGD. In addition, 2R-WGD and FSGD seem to have different duplicate retention rate, for example, both tetraspanins and Hox genes had had 75% retention rate after 2R-WGD but 25-30% after FSGD. So it may not be surprising that 2R-WGD and FSGD had different effect on the prevalence of positive selection. One interesting possibility is that the lingering effect of 2R-WGD lasted 300-400 million years and hence eclipsing the effect of FSGD. An alternative possibility is that different choices of gene families for tests may make a different result. Specifically, our work focused on 38 genes from one family that probably suitable for preserving the imprint of ancient sequence evolution, whereas Studer *et al*'s work concerned a set of 117 FSGD-related genes which are biased to genes of high sequence conservation and retention rate [32]. Anyway, the final answer requires the analysis of more genes and gene families.

## 4 Conclusions

In this study, we demonstrate how the 2R-WGD affected the gene retention, losses and positive selection on the bony vertebrate tetraspanin superfamily. Based on these results, we argue that a gene family which has high duplicate retention rate after WGD may not lead to the increase of family size; instead, it may just reshape the family composition. This is a new finding about the effect of WGD on the fate of multi-gene families. We also detect a massive wave of gene losses and positive selection that struck vertebrate tetraspanins right after 2R-WGD. To our knowledge, this phenomenon (especially the massive wave of positive selection) has not been detected in early studies [31-37]. So, we argue that the effect of WGD on positive selection should not be underestimated, which could be significantly more extensive and intensive than that of speciation. We finally propose that although the influence of WGD are gradually diminished, the influencing time of WGD could be much longer than we thought before; in terms of tetraspanins, the lingering influence of 2R-WGD on gene losses and positive selection might last for 300-400 million years.

## 5 Methods

### 5.1 Identification of tetraspanin sequences

Predicted transcripts of 22 deuterostome genome sequences were downloaded from the Ensembl FTP site [66], including *Dasypus novemcinctus *(Dn), *Equus caballus *(Ec), *Homo sapiens *(Hs), *Monodelphis domestica *(Md), *Mus musculus *(Mm), *Rattus norvegicus *(Rn), *Ornithorhynchus anatinus *(Oa), *Gallus gallus *(Gg), *Taeniopygia guttata *(Tg), *Anolis carolinensis *(Ac), *Xenopus tropicalis *(Xt), *Gasterosteus aculeatus *(Gac), *Danio rerio *(Dr), *Oryzias latipes *(Ol), *Takifugu rubripes *(Tr), *Tetraodon nigroviridis *(Tn), *Branchiostoma floridae *(Bf), *Ciona intestinalis *(Ci), *Ciona savignyi *(Cs). Predicted transcripts of the genome of *Strongylocentrotus purpuratus *(Sp) were downloaded from the NCBI FTP site. Unique gene data sets of *Xenopus laevis *(Xl), *Strongylocentrotus purpuratus *(Sp), *Ciona intestinalis *(Ci), *Ciona savignyi *(Cs) were downloaded from the NCBI FTP site. Besides, some sequences from other species were also used, including *Leucoraja erinacea *(Le), *Squalus acanthias *(Sa), *Meleagris gallopavo *(Mg), *Lachesis muta *(Lm), *Cyprinus carpio *(Cc), *salmo salar *(Ss) and *Oncorhynchus mykiss *(Om). Identification of tetraspanin sequences from these data was performed by using the PSSM model pfam00335 and the stand-alone RPS-BLAST [67]. In order to build a reference set of tetraspanin, we set out to find all tetraspanin sequences from human, mouse, zebrafish, *C. intestinalis*, *B. floridae *and *S. purpuratus*. Therefore, for these species, we also downloaded and scanned their *ab initio *predicted transcripts, expressed sequence tags and genomic sequences.

Because of the structure features (see Background), tetraspanin sequences generally have higher quality than many other gene families, but manual sequence inspection and correction is still needed. Fortunately, the sequence, protein architecture and intron positions of tetraspanins are highly conserved within ortholog lineages (in practice, we can divide tetraspanins in 4 TM, 2EC, and the large EC can be further divided into 4-6 portions (Figure 1B &1C), so by dividing the sequence into these short portions it is easy to detect and correct the sequence problem. Three problems could be encountered: incorrect splicing sites, fragmented genes and sequencing gaps. Briefly, four procedures were performed: 1) FGENEH or FGENEH+ with close homologs were used to re-predict the transcript to fix incorrect splicing and fragmental genes; 2) mRNA/EST was used to correct sequences; 3) when multiple different sequences for a same gene were available, high-quality sequence from close species were used as referee to decide which one and or which portion was more accurate; 4) incomplete genes with sequencing gaps or not corrected genes were deleted.

### 5.2 Protein sequence-based phylogenetic reconstruction

Only the sequences of "tetraspanin core" (4TM+SEL+LEL) were used for alignment and tree reconstruction. Clustalw 1.83 with all default settings was used to produce multiple alignments. Unlike the previous analysis [2], in order not to introduce subjective bias, manual alignment editing was minimized. Minimum-evolution (ME) trees were built by using Mega v4.1 [68], with 1000 bootstrap tests and pairwise deletion. Both poisson correction model and p-distance model were used in the ME method. Maximum-likelihood (ML) method was conducted by using Phylip v3.65, with 100 bootstrap tests, Jones-Taylor-Thornton model, no gamma correction, and 2 times of jumble for Figure S3 (but 10 times for the analyses of 17 individual trees). Maximum parsimony (MP) method was conducted by using Phylip v3.65, with 100 bootstrap tests and 10 times of jumble. Bayesian protein trees were carried out using MrBayes v3.1, with amino acid substitution models recommended by the program. All other not mentioned parameters were set as defaults. In this study, ME trees were presented as bootstrapped original trees with branch length, whereas ML and MP trees were presented as bootstrapped consensus trees without branch length.

### 5.3 Codon usage, Intron configuration analysis and syntenic context analysis

Codon bias and GC contents was analyzed using CodonW program http://mobyle.pasteur.fr/cgi-bin/portal.py?form=codonw. Intron positions for each gene were identified by using stand-alone SPLIGN program http://www.ncbi.nlm.nih.gov/sutils/splign/ and marked on the protein sequences using a home-made Perl script. Syntenic context analysis was carried out manually on the UCSC gene sorter and genome browser http://genome.ucsc.edu/[69].

### 5.4 Reconstruction of ohnolog lineages and their duplicating patterns

Paralogs arisen from WGDs are called ohnologs. We selected all duplicate pairs, triplets or quartets that were created between the speciation of vertebrates and bony vertebrates, and then used the UCSC gene sorter and genome browser http://genome.ucsc.edu/[69] to determine if these duplicates are located on syntenic regions. Duplicates sharing synteny were considered ohnolog pairs or triplets (note: no ohnolog quatets found). The next step is to determine the duplicating patterns by constructing phylogenetic trees. The theoretical pattern of four ohnologs arising from 2R-WGD should be (AB)(CD), but 2R-WGD happened more than 500 Mya ago and the interval between 2R-WGDs was rather short, so divergence and gene loss may distorted the ideal pattern. To reconcile the duplicating patterns, we not only constructed trees from tetraspanin ohnologs, but also constructed trees from those ohnolog genes co-localized with tetraspanin genes. Likelihood ratio tests were used to compare the fitness of different phylogenetic topologies using PAML program [70].

### 5.5 Detection of positive selection and statistic analysis

Based on the phylogeny obtained from reference tetraspanin seqences, we produced extended alignments and phylogenetic trees for each ancestral vertebrate tetraspanin lineage (seventeen in total) by including more species. For each tetraspanin ortholog lineage, we included at least (but not limited) five mammal species, four teleost species, and as many species as possible for reptiles and amphibians. Because of the conserved protein sequence and architecture, it is easy to produce high-quality alignments for each tetraspanin lineages. Protein-based alignments were converted to nucleotide alignments by using RevTrans [71]. To guarantee reliable likelihood tests, manual sequence correction was conducted on alignments. Reference sequences were used as criteria to detect potential problems by eyes, and the problematic sequences were either deleted or corrected, depending on their dispensability in an alignment. The alignment browser of Mega 4 was used as the basic tool for sequence editing [68]. Highly divergent sequences were also deleted from the alignments.

PAML 4 program was used to detect positive selection [70]. In a codon model, d_N_/d_S _> 1 (ω > 1) is considered as indicator of positive selection. In this study, the basic model (one-ratio), two site-specific models (nearly-neutral and positive-selection) and the branch-site model A were applied on each tree. The branch-site model A has been reported to be more powerful and sensitive than the site-specific model and the branch-specific models [72]. In a test using branch-site model A, the branch being tested for positive selection is called the foreground branch, and the other branches in the tree are called the background branches. Likelihood ratio test (LRT) is used to evaluate whether significant positive selection happens on the foreground branch. Two LRTs may be constructed: 1) to compare the model A to the nearly-neutral model or 2) to compare the model A to the modified model A with ω = 1 fixed. The first LRT is known to have high false positive rate because it does not distinguish between positive selection and relaxed selective constraints [72], hence the second LRT is preferred. The test is done by comparing 2ΔlnL to χ_1_^2 ^or a 1:1 mixture of 0 and χ_1_^2^. To guide against violations of model assumptions [47], the more conservative test with χ_1_^2 ^was used in this study. To avoid the known convergence problems (for example, sometimes the parameters may get stuck at a corner of parameter space), we ran the program twice with two set of parameters, the first set is the defaults, whereas the second set is the parameters inferred from the one-ratio model. Since multiple branches from the same tree have been tested, we used the Hochberg multiple testing correction method [46] to control the family-wise error rate. Except for the construction of reference trees, other processes were automated using a home-made Perl script. All statistic analysis following the evaluation of positive selection was conducted by using Microsoft Office Excel.

### 5.6 Simulations

Those deep ancient branches (the duplication branches right after 2R-WGD) may be suffered from d_S _saturation, which in return may cause false detection of positive selection on these branches. To gain insight into the false positive rates on these branches, we used EvolveNSsite from PAML4 to generate alignments under the nearly-neutral (M1a) model [70], and ran selection tests on the duplication branches of these alignments using a stringent branch-site model as described in Section 5.5. Thirteen tetraspanin families which contain at least two duplication branches were used for simulation. For each family five alignments were simulated, using the same parameters derived from the corresponding family. These parameters include two categories, one derived directly from the original alignment (sequence number, sequence length, tree topology, and codon usage), the other inferred from the original alignment under the nearly-neutral (M1a) model using PAML4 (branch length, d_N_/d_S _ratio ω, Ts/Tv ratioκ, and the proportion of site with ω = 1). Furthermore, to correct for possible underestimation of the branch length, we generated and tested five additional simulated alignments for each family with all the same parameters except multiplying the branch length by 1.5.

### 5.7 Detection of rate shifts among sites using protein-based methods

There are higher chance for those deep ancient branches (the duplication branches right after 2R-WGD) to suffer from d_S _saturation and codon model violation. If so, the power and accuracy of selection tests on these branches based on codon models may be impaired [47]. Alternatively, maximum-likelihood approaches based on protein sequences are able to better contain divergent sequences in theory [45], although as a trade-off, they lose part of the codon substitution information. To provide complement and confirmation to the codon models used here, we incorporated two protein-based maximum-likelihood approaches to detect the alteration of selection constraints (or the rate shift) on those tetraspanin ohnolog lineages produced by 2R-WGD. The first method is Gu's method (Gu99) that is implemented in the software DIVERGE2 [50], which detect specific amino acid sites under functional divergence between two paralog lineages after gene duplication [73,74]. This method tests whether the coefficient of functional divergence θ of two selected lineages is significantly larger than 0. If so, then there is significant functional divergence between two lineages, and the sites contributed to divergence are also inferred using a probabilistic model. The second method is based on a covarion model that is implemented in the software RASER2, which allows raft shifts to vary among lineages and permits to identify the rate-shifting sites by empirical Beyesian inference [51]. Here we used RASER2 to compare the lineage-specific model (invoked by using the parameter file "raser.stochasticMapping.params", and specifying a tetraspanin ohnolog lineage in this file, which thus enables rate shifts on the specified lineage) with the null model (invoked by using the parameter file "null.params", which does not enable rate shifts). All other parameters were set to defaults. To avoid the known convergence problems, we ran the program for at least twice for each lineage and each model on different platforms. Then likelihood-ratio tests (LRT) were performed to determine whether the lineage-specific model fit the data significantly better than the null model. If so, rate shifts on the specified lineage is significant. Protein-based analyses were only conducted on duplication branches (or ohnolog lineages) from 13 tetraspanin families with at least two duplication branches. Used alignments and tree topologies are the same as used in codon-based analyses.

### 5.8 Data availability

The reference set of deuterostome tetraspanins including six genomes (human, mouse, zebrafish, *C. intestinalis*, *B. floridae *and *S. purpuratus*) and some other selected sequences were presented in Additional file 2. All alignments used for phylogenetic analyses and positive seletion tests were presented in Additional file 6.

## Abbreviations

LEL: large extracellular loop; LRT: likelikhood ratio test; SEL: small extracellular loop; TEM: tetraspanin-enriched microdomain; TM: transmembrane; TSPAN: tetraspanin; WGD: whole genome duplication; 2R-WGD: two rounds of WGD at the origin of vertebrates; FSGD: fish-specific WGD.

## 6 Authors' contributions

SH and AX conceited the analysis and drafted the manuscript. SH prepared all scripts and performed most of the analyses. HZ carried out the Bayesian tree inference. CZ and YT collected raw data, and provided technical support on computation and statistics. All authors read and approved the final manuscript.

## Supplementary Material

Additional file 1 **Supplemental text and figures, including**:. Figure S1. Protein ME tree of reference tetraspanins, using poisson correction distance.
Figure S2. Protein ME tree of reference tetraspanins, using p-distance.
Figure S3. Protein ML tree of reference tetraspanins.
Figure S4. Protein MP tree of reference tetraspanins.
Figure S5-S21, One-ratio codon trees of 17 ancestral tetraspanin lineage inferred using PAML4; used for positive selection tests.
Figure S22-S36, One-ratio codon trees of other tetraspanin lineage used for positive selection tests.
Figure S37-S53, Bayesian inference protein trees of 17 ancestral tetraspanin lineage inferred using MrBayes. Click here for file

Additional file 2 **Supplemental Table 1**. A reference collection of deuterostome tetraspanin proteins. Click here for file

Additional file 3 **Supplemental Table 2**. Results of likelihood ratio tests for positive selection using PAML 4. Click here for file

Additional file 4 **Supplemental Table 3**. Detection for rate shifts among sites between tetraspanin ohnolog lineages using DIVERGE2. Click here for file

Additional file 5 **Supplemental Table 4**. Detection for rate shifts among sites on tetraspanin ohnolog lineages using RASER2. Click here for file

Additional file 6 **All used alignments (alignment.zip)**. Click here for file
