# Supplementary material for: The evolution of vertebrate tetraspanins: gene loss, retention, and massive positive selection after whole genome duplications
Source: BMC Evol Biol. 2010 Oct 13;10:306. doi: 10.1186/1471-2148-10-306 (PMC2965184; doi:10.1186/1471-2148-10-306)
Supplement: Additional file 1 — Supplemental text and figures, including:. Figure S1. Protein ME tree of reference tetraspanins, using poisson correction distance. Figure S2. Protein ME tree of reference tetraspanins, using p-distance. Figure S3. Protein ML tree of reference tetraspanins. Figure S4. Protein MP tree of reference tetraspanins. Figure S5-S21, One-ratio codon trees of 17 ancestral tetraspanin lineage inferred using PAML4; used for positive selection tests. Figure S22-S36, One-ratio codon trees of other tetraspanin lineage used for positive selection tests. Figure S37-S53, Bayesian inference protein trees of 17 ancestral tetraspanin lineage inferred using MrBayes. [file 1471-2148-10-306-S1.PDF]

The evolution of vertebrate tetraspanins: gene loss, retention, and massive positive selection after whole genome duplications

Supplemental materials

Page 2 to 4. Supplemental text.

Page5. Figure S1. Protein ME tree of reference tetraspanins, using poisson correction distance.

Page 6. Figure S2. Protein ME tree of reference tetraspanins, using p-distance.

Page 7. Figure S3. Protein ML tree of reference tetraspanins.

Page 8. Figure S4. Protein MP tree of reference tetraspanins.

Page 9 to 25. Figure S5-S21, One-ratio codon trees of 17 ancestral tetraspanin lineage inferred using PAML4; used for positive selection tests.

Page 26 to 32. Figure S22-S36, One-ratio codon trees of other tetraspanin lineage used for positive selection tests.

Page 33 to 49. Figure S37-S53, Bayesian inference protein trees of 17 ancestral tetraspanin lineage inferred using MrBayes.

Page 50. Figure S1. Protein ME tree of reference tetraspanins, using poisson correction distance.

Page 51. Figure S2. Protein ME tree of reference tetraspanins, using p-distance.

Page 52. Figure S3. Protein ML tree of reference tetraspanins.

Page 53. Figure S4. Protein MP tree of reference tetraspanins.

Supplemental text

A reference set of high-quality deuterostome tetraspanins - results of manual correction

- A total of 33 and 32 tetraspanins were identified from human and mouse, respectively. One mouse gene has been annotated as pseudogene. Human and mouse genome sequences represent the best quality of all genomes used here and as a result, no manual correction is required for these sequences.
- Garcia-Espana *et al* reported a set of 40 zebrafish tetraspanins [28]. In this study ten more sequences have been identified, so there are a total of 50 tetraspanin genes in zebrafish. Of these genes, several need manual correction, including three partially annotated genes and two genes missing on the current genome version.
- A total of 33 *C. intestinalis* tetraspanins have been found, with one of them absent from the current draft genome. Many sequences are problematic, two of which failed the manual correction and hence were excluded from further analysis.
- A total of 39 amphioxus tetraspanins have been identified, 34 of which have EST evidence. This number is more than that of mammals, birds and amphibian *X. tropicalis*, but less than that of zebrafish. More than half the gene models are problematic but all have been corrected manually.
- The *S. purpuratus* draft genome contributes 29 tetraspanins, but because of the poor quality of the genome sequence it is not known if this is a complete set. Most *S. purpuratus* tetraspanin sequences are problematic and several of them are unrepairable at present. Therefore, only 18 sequences are qualified for further analysis.

Reconstruction of 17 ancestral vertebrate tetraspanin lineages - supporting evidence for the lineages which are most difficult to define

- The relation between the vertebrate TSPAN3 and its basal deuterotome orthologs have weak bootstrap value (<70), and its *S. purpuratus* counterpart even separates from the lineage due to long-branch attraction (Fig S1). However, the exact intron configuration and similar syntenic context (i.e. hect/RLD2-TSPAN3-RPL24-NCKX, etc) confirm their ortholog relationships.
- TSPAN10 and RDS/peripherin are previously mistaken as sister lineages [28], but it is an artifact due to long branch attraction. Their protein identity is low than 20%. TSPAN10 belongs to the “8Cys” LEL type, whereas RDS/perpherin is a regular “6a” type. Their intron configurations are completely different.
- In the TSPAN18 lineage, TSPAN16 is the most divergent member. It is only slightly closer to TSPAN1 than other tetraspanins and sometimes separated from TSPAN1 and TSPAN18 in the comprehensive tree (Fig S1). However, syntenic analysis indicates that TSPAN1, TSPAN16 and TSPAN18 are derived from the same genomic region through two rounds of WGD (Fig 3).
- The vertebrate TSPAN12 lineage has weak homologs in amphioxus and *C. intestinalis* (Fig 4K). However, since they have poor protein identity (<30%), no conserved syntenic context and no similar intron configuration, their ortholog relationships are rejected for safety.
- Similar situation is also observed in the uroplakin lineage (Fig 4K), where the *C.*

intestinalis tetraspanin shares only 20% protein identity with vertebrate uroplakins, hence an ortholog relationship is rejected.

- The vertebrate TSPAN8 lineage has 14 amphioxus homologs (TSPAN8-like) and 5 *C. intestinalis* homologs (Fig S1). They apparently share a common origin because they all have similar LEL structures (type 6b1 and 6c) and most of them reside in the TSPAN4 cluster (Fig 3). However, since the TSPAN8-like family of amphioxus and *C. intestinalis* have undergone rapid gene duplications, losses and diversification, it is impossible to determine which TSPAN8-like gene is the true ortholog of vertebrate TSPAN8.
- The CD37 lineage (including CD37, CD82 and TSPAN19) is a vertebrate-specific tetraspanin lineage. TSPAN19 is the diverged one and sometimes separated from CD37 and CD82 in the comprehensive tree (Fig S1). However, all three members share unique intron configuration, unique LEL cysteine configuration (type 6b2) and similar syntenic context (Fig 3).
- The CD9 lineage is a vertebrate-specific tetraspanin lineage, which included CD9, CD81, TSPAN2, TSPAN32 and CD9-like. Three of them, namely CD9, CD81 and TSPAN2, shared unique intron configuration and LEL structure (type 4c), and are derived from two rounds of WGD (Fig 3). As for TSPAN32, it looks like a "rogue" and was previously placed together with uroplakins [28]. Indeed, it is too divergent to cluster with the CD9 lineage (Fig S1), but it has the intron configuration and LEL structure specific to the CD9 lineage. Besides, TSPAN32 is co-localized with CD81 and absent in amphibians and fishes. So TSPAN32 should be derived from CD81 by independent tandem duplication on the common stem of the bird-mammal lineage. Similarly, the zebrafish CD9-like is derived from CD9 by independent duplication.

The monophyletic 8-cysteine super-lineage and the vertebrate TSPAN4 gene cluster - supporting evidence and discussion

- The TSPAN4 gene cluster suggests that four common cluster members, CD151, TSPAN4, TSPAN18 and TSPAN8(-like), should be produced from a single gene through ancient tandem duplications occurred before the divergence of chordata and echinodermata.
- There are 12 TSPAN8-like genes in the amphioxus TSPAN4 cluster and only one (TSPAN8) in the vertebrate part. It is sufficient to suggest that the vertebrate TSPAN8 lineage is originated within the gene cluster.
- Members of the CD9 and CD37 lineages are distributed on different paralogous regions of the modern vertebrate TSPAN4 cluster. Since there are no invertebrate orthologs for CD9 or CD37, they should be derived from certain cluster members and subsequently underwent rapid diversification on the vertebrate stem.
- Analysis of the amphioxus TSPAN4 cluster shows that it contains 3/4 of the amphioxus-specific tetraspanins, suggesting that this region is the major factory for producing new tetraspanins. In addition to CD9 and CD37, there were three more vertebrate tetraspanin lineages (uroplakin, CD63 and TSPAN13) having members on the TSPAN4 cluster, suggesting their possible relationships with the cluster.
- In the chordate phylum, the TSPAN4 cluster not only produces many new tetraspanins, but also seems to produce all non-canonical LEL structures, such as type 4a (human CD53), 4c (vertebrate CD9), 6b2 (vertebrate CD37), 6b1 (amphioxus TSPAN8-like-B) and 6c (amphioxus TSPAN8-like-A and vertebrate TSPAN8). Type 4c, 6b1, 6b2 and 6c share

some similarity, implying that the tetraspanins of these structures might have a kind of connection, for example, all derived from a gene with non-canonical 6-cysteine LEL structure. Actually, CD9 and CD37.

Huang_FigS1

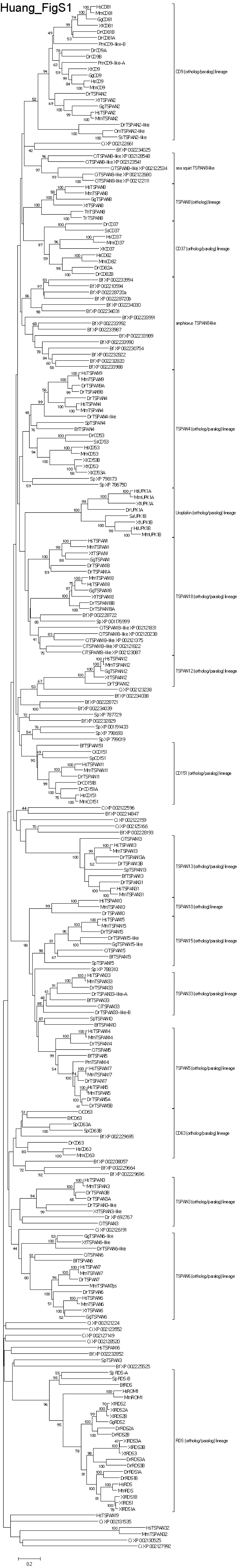

CD9 (ortholog/paralog) lineage
sea squirt TSPAN8-like
TSPAN8 (ortholog) lineage
CD37 (ortholog/paralog) lineage
amphioxus TSPAN8-like
TSPAN4 (ortholog/paralog) lineage
Uroplakin (ortholog/paralog) lineage
TSPAN18 (ortholog/paralog) lineage
TSPAN12 (ortholog/paralog) lineage
CD151 (ortholog/paralog) lineage
TSPAN13 (ortholog/paralog) lineage
TSPAN10 (ortholog) lineage
TSPAN15 (ortholog/paralog) lineage
TSPAN33 (ortholog/paralog) lineage
TSPAN5 (ortholog/paralog) lineage
CD63 (ortholog/paralog) lineage
TSPAN3 (ortholog/paralog) lineage
TSPAN6 (ortholog/paralog) lineage
RDS (ortholog/paralog) lineage
0.2

Huang_FigS2

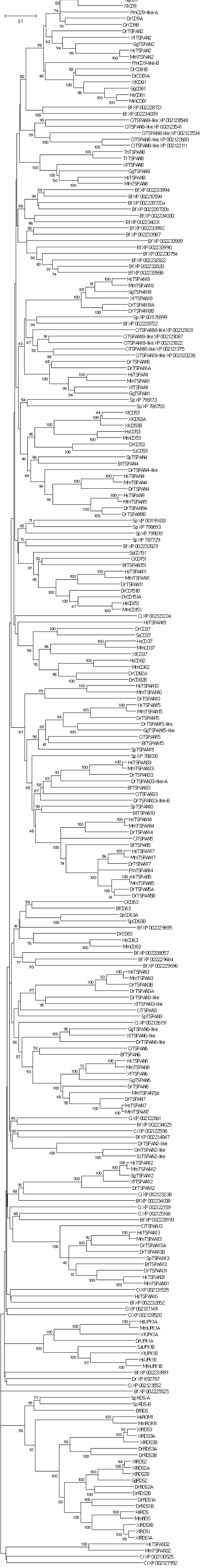

Huang_FigS3

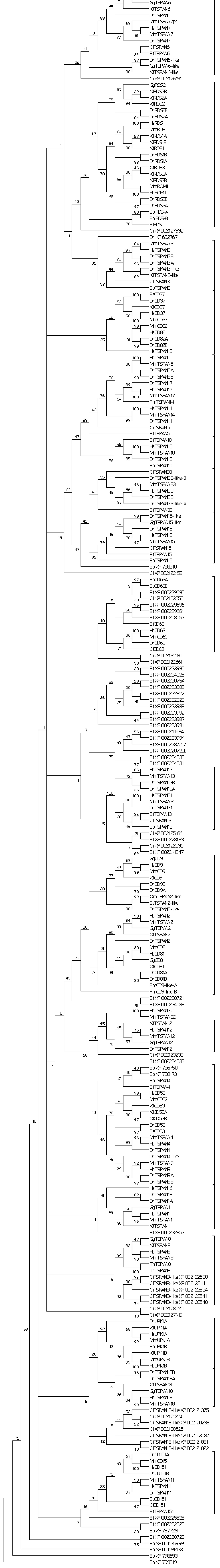
MmTSPAN6
HsTSPAN6
GgTSPAN6
XtTSPAN6
DrTSPAN6
MmTSPAN7ps
HsTSPAN7
MmTSPAN7
DrTSPAN7
CiTSPAN6
BfTSPAN6
DrTSPAN6-like
GgTSPAN6-like
XtTSPAN6-like
Ci XP 002126191
GgRDS2
XlRDS2B
XlRDS2A
XtRDS2
DrRDS2B
DrRDS2A
HsRDS
MmRDS
XlRDS1A
XlRDS1B
XtRDS1
DrRDS1B
DrRDS1A
XtRDS3
XlRDS3A
XlRDS3B
MmROM1
HsROM1
DrRDS3B
DrRDS3A
Sp RDS-A
Sp RDS-B
BfRDS
Ci XP 002127992
Dr XP 692767
MmTSPAN3
HsTSPAN3
DrTSPAN3B
DrTSPAN3A
DrTSPAN3-like
XtTSPAN3-like
CiTSPAN3
SpTSPAN3
SsCD37
DrCD37
XtCD37
HsCD37
MmCD37
MmCD82
HsCD82
DrCD82A
DrCD82B
HsTSPAN19
HsTSPAN5
MmTSPAN5
DrTSPAN5A
DrTSPAN5B
DrTSPAN17
HsTSPAN17
MmTSPAN17
PmTSPAN14
HsTSPAN14
MmTSPAN14
DrTSPAN14
CiTSPAN5
BfTSPAN5
BfTSPAN10
HsTSPAN10
MmTSPAN10
DrTSPAN10
SpTSPAN10
CiTSPAN33
DrTSPAN33-like-B
MmTSPAN33
HsTSPAN33
DrTSPAN33
DrTSPAN33-like-A
BfTSPAN33
DrTSPAN15-like
GgTSPAN15-like
DrTSPAN15
HsTSPAN15
MmTSPAN15
CiTSPAN15
BfTSPAN15
SpTSPAN15
Sp XP 788310
Ci XP 002122159
SpCD63A
SpCD63B
Bf XP 002229695
Ci XP 002123552
Bf XP 002229696
Bf XP 002229664
Bf XP 002208057
BfCD63
HsCD63
MmCD63
DrCD63
CiCD63
Ci XP 002131535
Ci XP 002122661
Bf XP 002233990
Bf XP 002234025
Bf XP 002230754
Bf XP 002233988
Bf XP 002232822
Bf XP 002232820
Bf XP 002233989
Bf XP 002233992
Bf XP 002233987
Bf XP 002233991
Bf XP 002210594
Bf XP 002233994
Bf XP 002228720a
Bf XP 002228720b
Bf XP 002234030
Bf XP 002234031
HsTSPAN13
MmTSPAN13
DrTSPAN13B
DrTSPAN13A
HsTSPAN31
MmTSPAN31
DrTSPAN31
BfTSPAN13
CiTSPAN13
SpTSPAN13
Ci XP 002125166
Bf XP 002228193
Ci XP 002122596
Bf XP 002214847
GgCD9
HsCD9
MmCD9
XtCD9
DrCD9B
DrCD9A
OmTSPAN2-like
SsTSPAN2-like
DrTSPAN2-like
HsTSPAN2
MmTSPAN2
GgTSPAN2
XtTSPAN2
DrTSPAN2
MmCD81
HsCD81
GgCD81
XtCD81
DrCD81A
DrCD81B
PmCD9-like-A
PmCD9-like-B
Bf XP 002228721
Bf XP 002234039
HsTSPAN32
MmTSPAN32
XtTSPAN12
HsTSPAN12
MmTSPAN12
GgTSPAN12
DrTSPAN12
Ci XP 002123238
Bf XP 002234038
Sp XP 786750
Sp XP 798173
SpTSPAN4
BfTSPAN4
HsCD53
MmCD53
XtCD53
XlCD53A
XlCD53B
DrCD53
SsCD53
MmTSPAN4
HsTSPAN4
DrTSPAN4
DrTSPAN4-like
MmTSPAN9
HsTSPAN9
DrTSPAN9A
DrTSPAN9B
HsTSPAN16
DrTSPAN1B
DrTSPAN1A
GgTSPAN1
HsTSPAN1
MmTSPAN1
XtTSPAN1
Bf XP 002232852
GgTSPAN8
XtTSPAN8
HsTSPAN8
MmTSPAN8
TnTSPAN8
TrTSPAN8
CiTSPAN8-like XP 002122680
CiTSPAN8-like XP 002122111
CiTSPAN8-like XP 002122534
CiTSPAN8-like XP 002123541
CiTSPAN8-like XP 002128548
Ci XP 002128520
Ci XP 002127149
DrUPK1A
XtUPK1A
HsUPK1A
MmUPK1A
SaUPK1B
XtUPK1B
MmUPK1B
HsUPK1B
DrTSPAN18B
DrTSPAN18A
XtTSPAN18
GgTSPAN18
HsTSPAN18
MmTSPAN18
CiTSPAN18-like XP 002121375
Ci XP 002121224
CiTSPAN18-like XP 002120238
Ci XP 002130525
CiTSPAN18-like XP 002123087
CiTSPAN18-like XP 002121831
CiTSPAN18-like XP 002121822
DrCD151A
MmCD151
HsCD151
DrCD151B
MmTSPAN11
HsTSPAN11
DrTSPAN11
SpCD151
CiCD151
BfTSPAN151
Bf XP 002225525
Bf XP 002232829
Sp XP 787729
Bf XP 002228722
Sp XP 001176999
Sp XP 001191433
Sp XP 798693
Sp XP 799019

Huang_FigS4

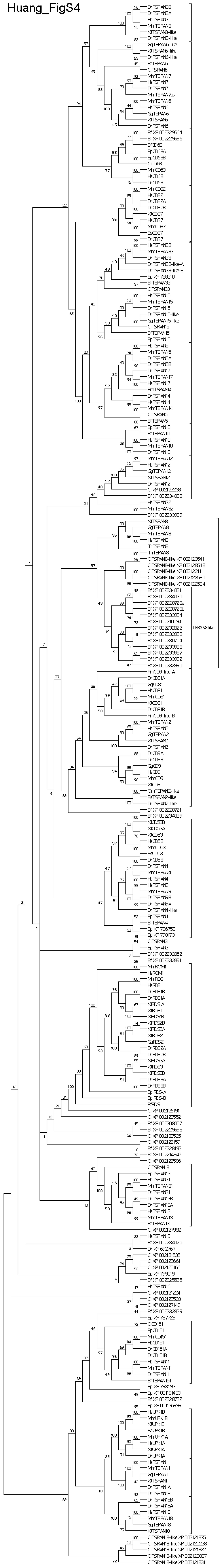

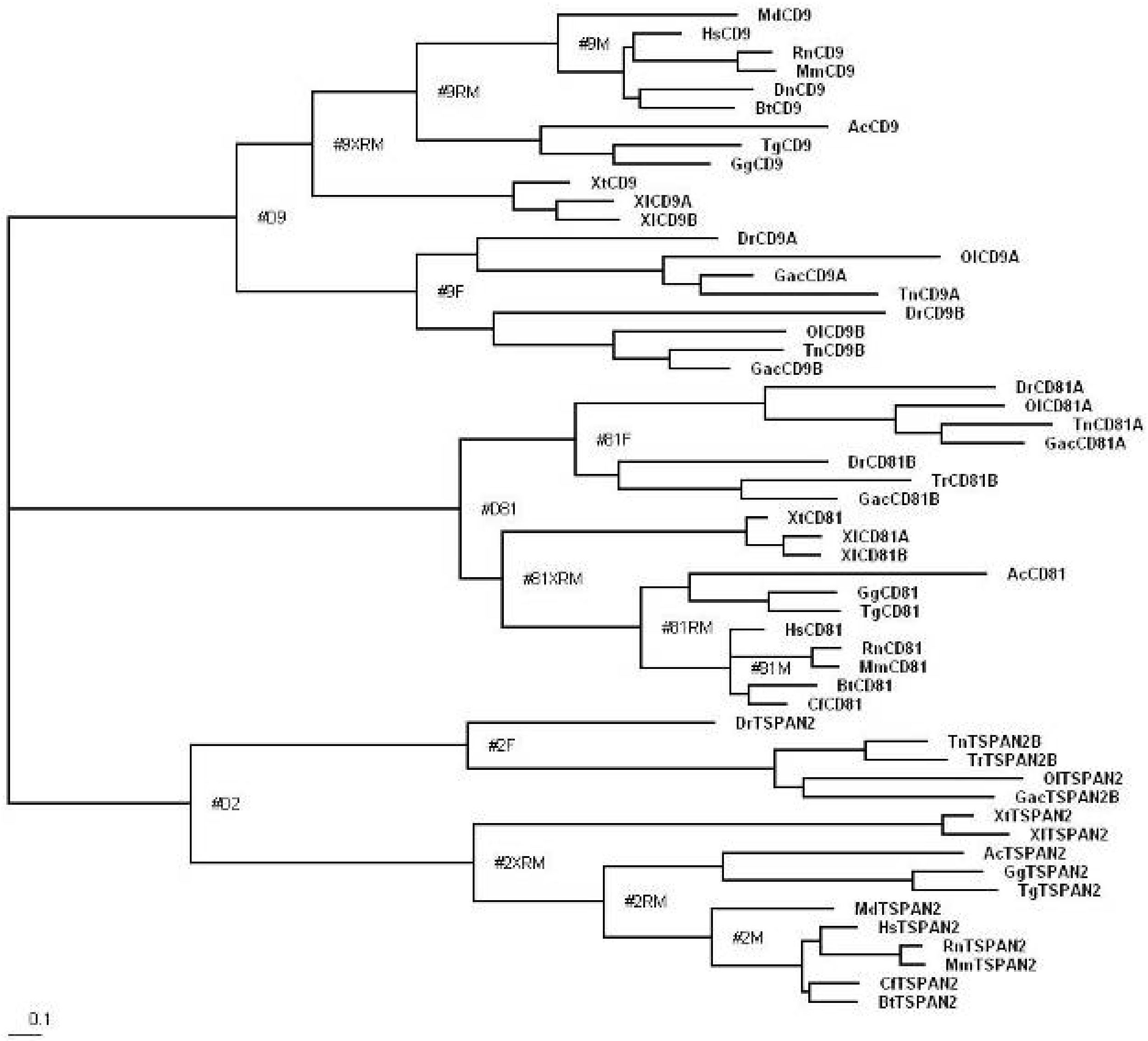

Figure S5. One-ratio tree of CD9 family, used for testing positive selection. The branches for testing are labeled.

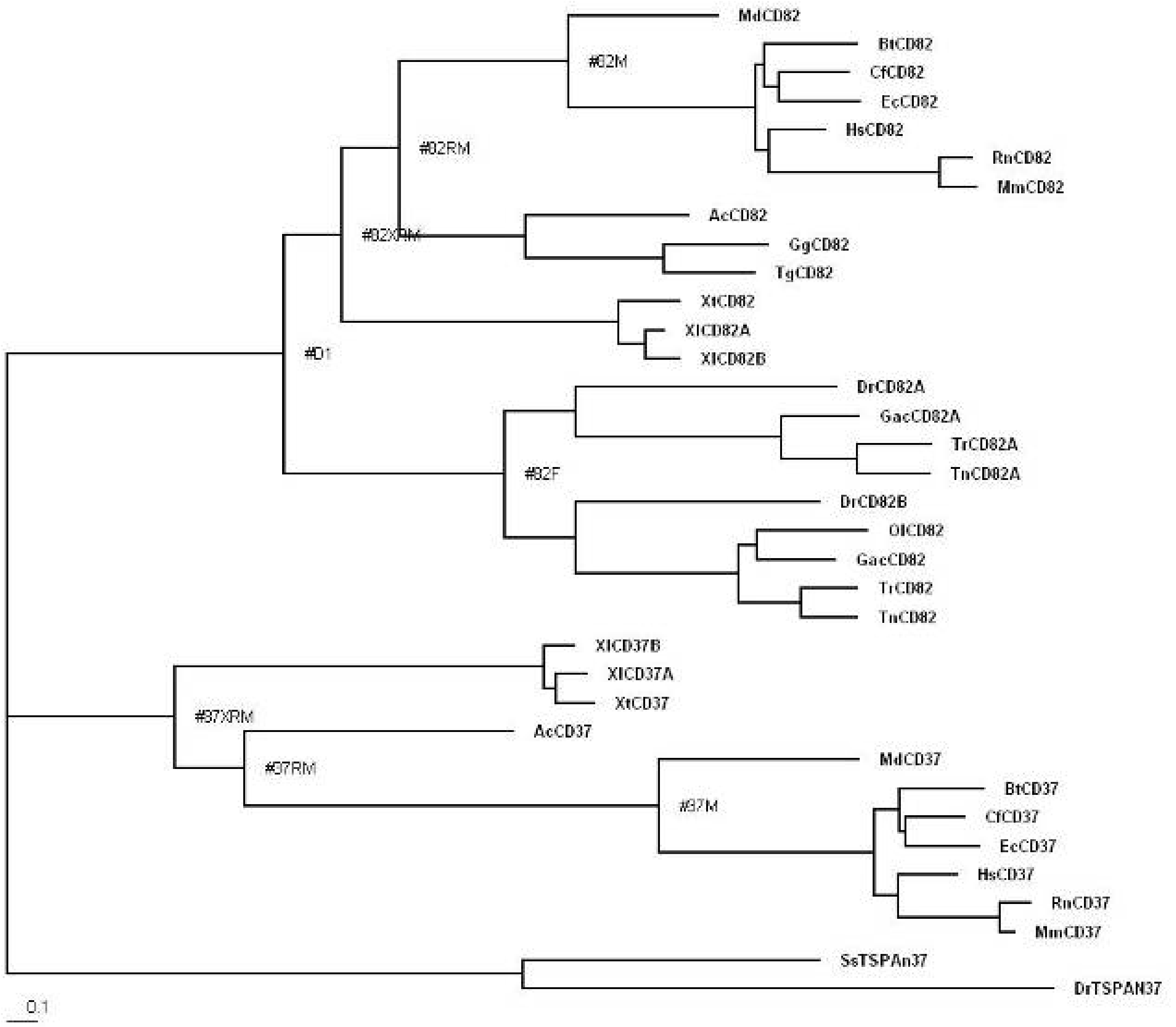

Figure S6. One-ratio tree of CD37 family, used for testing positive selection. The branches for testing are labeled.

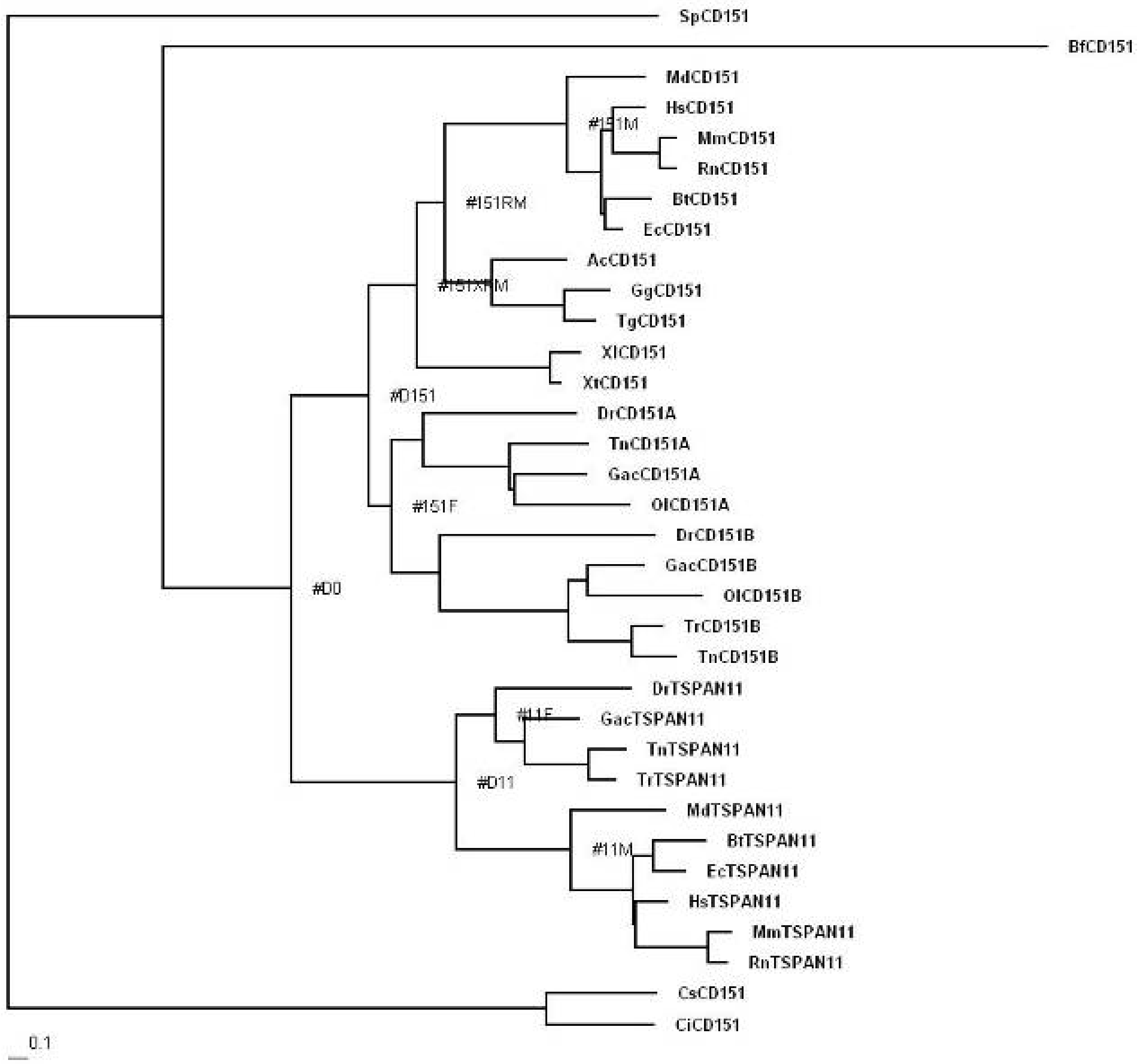

Figure S7. One-ratio tree of CD151 family, used for testing positive selection. The branches for testing are labeled.

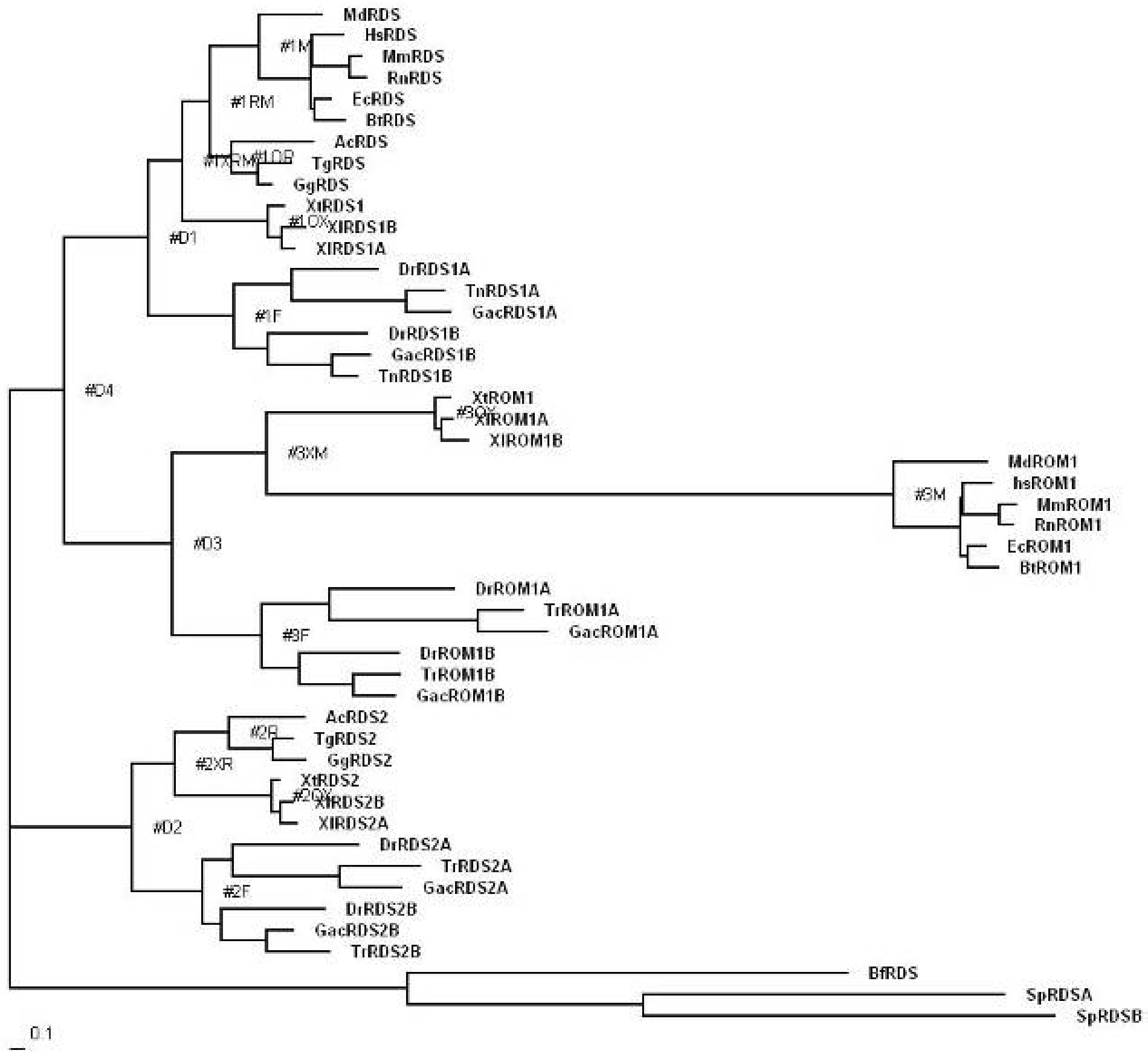

Figure S8. One-ratio tree of RDS family, used for testing positive selection. The branches for testing are labeled.

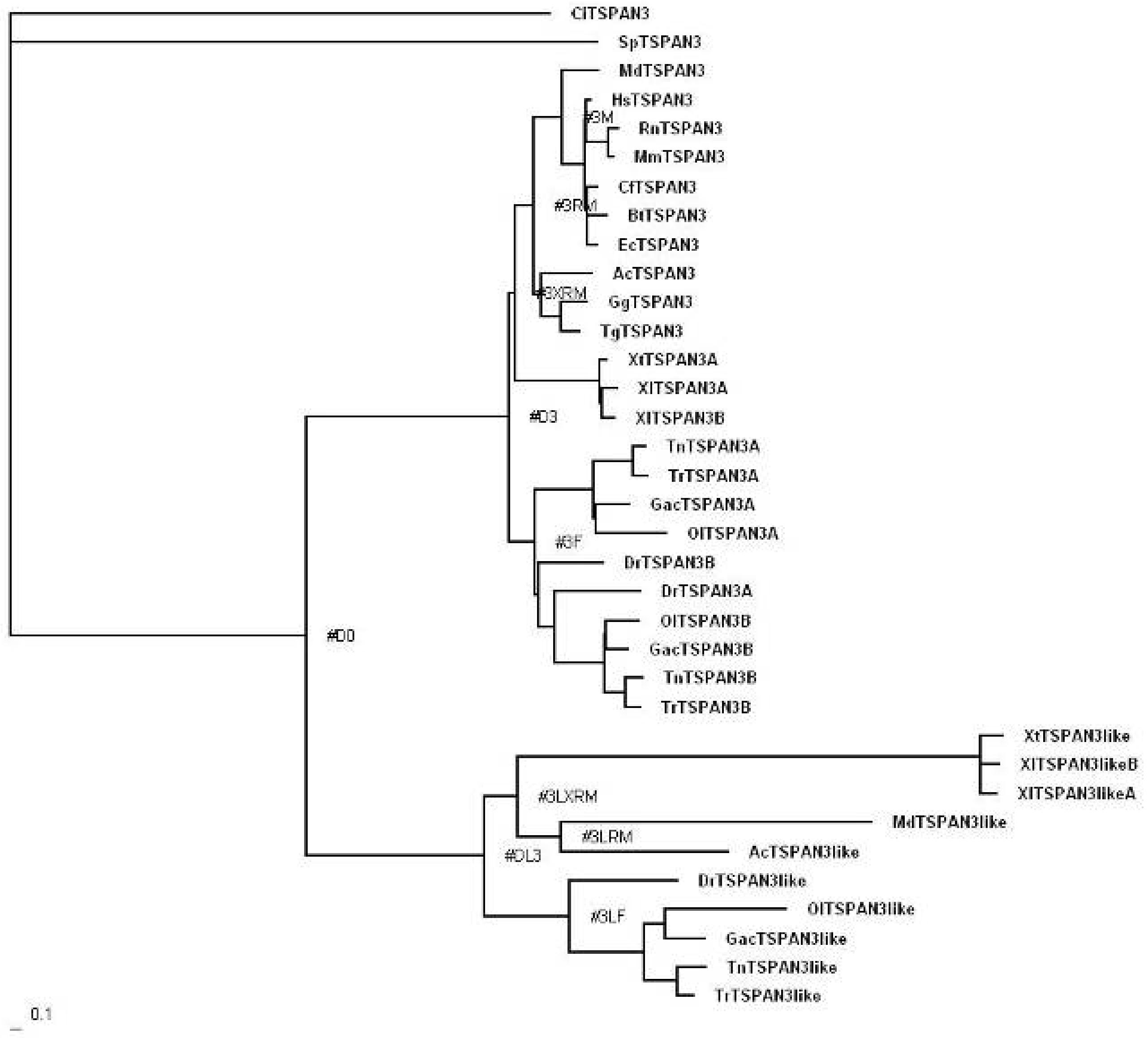

Figure S9. One-ratio tree of TSPAN3 family, used for testing positive selection. The branches for testing are labeled.

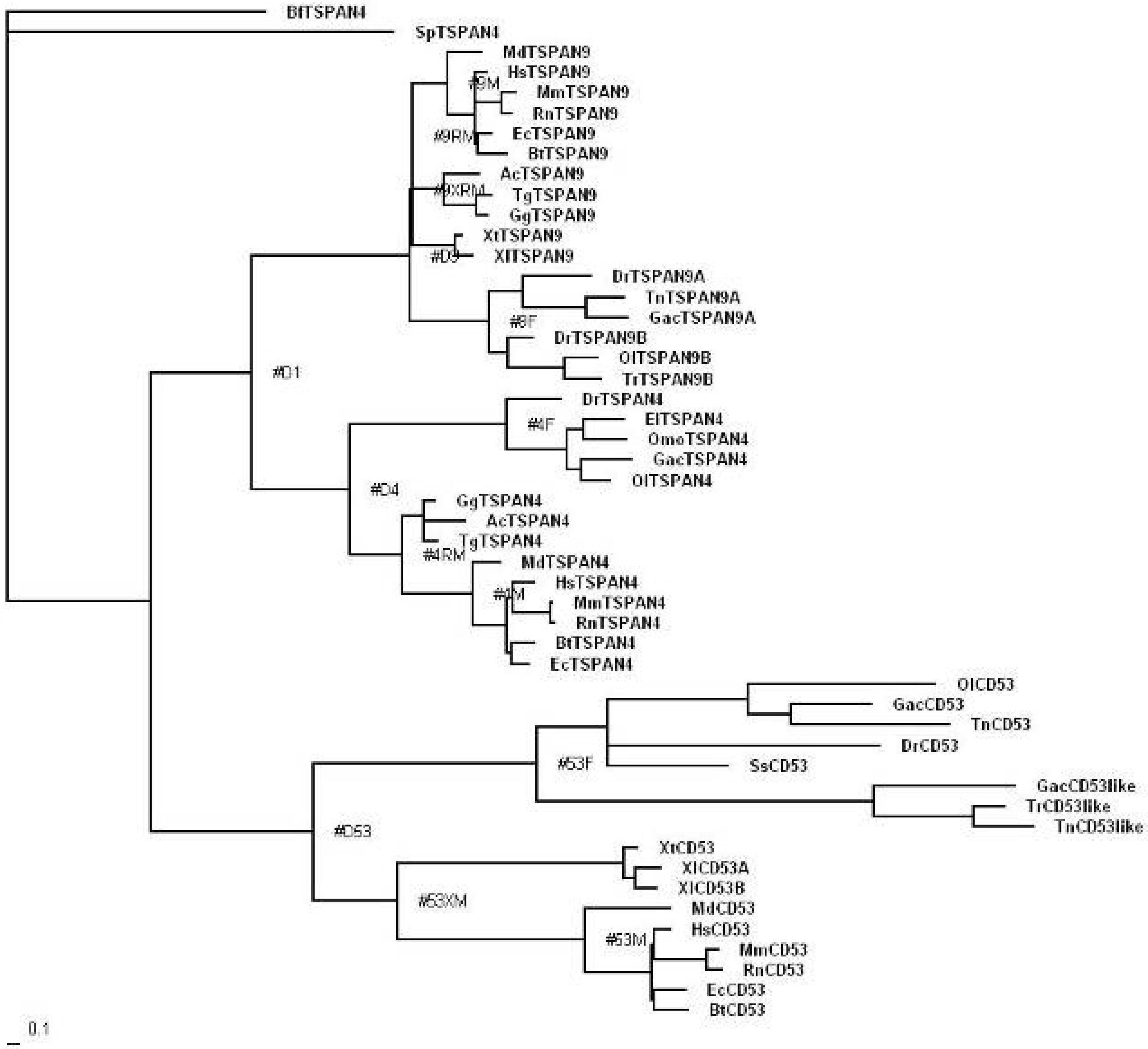

Figure S10. One-ratio tree of TSPAN4 family, used for testing positive selection. The branches for testing are labeled.

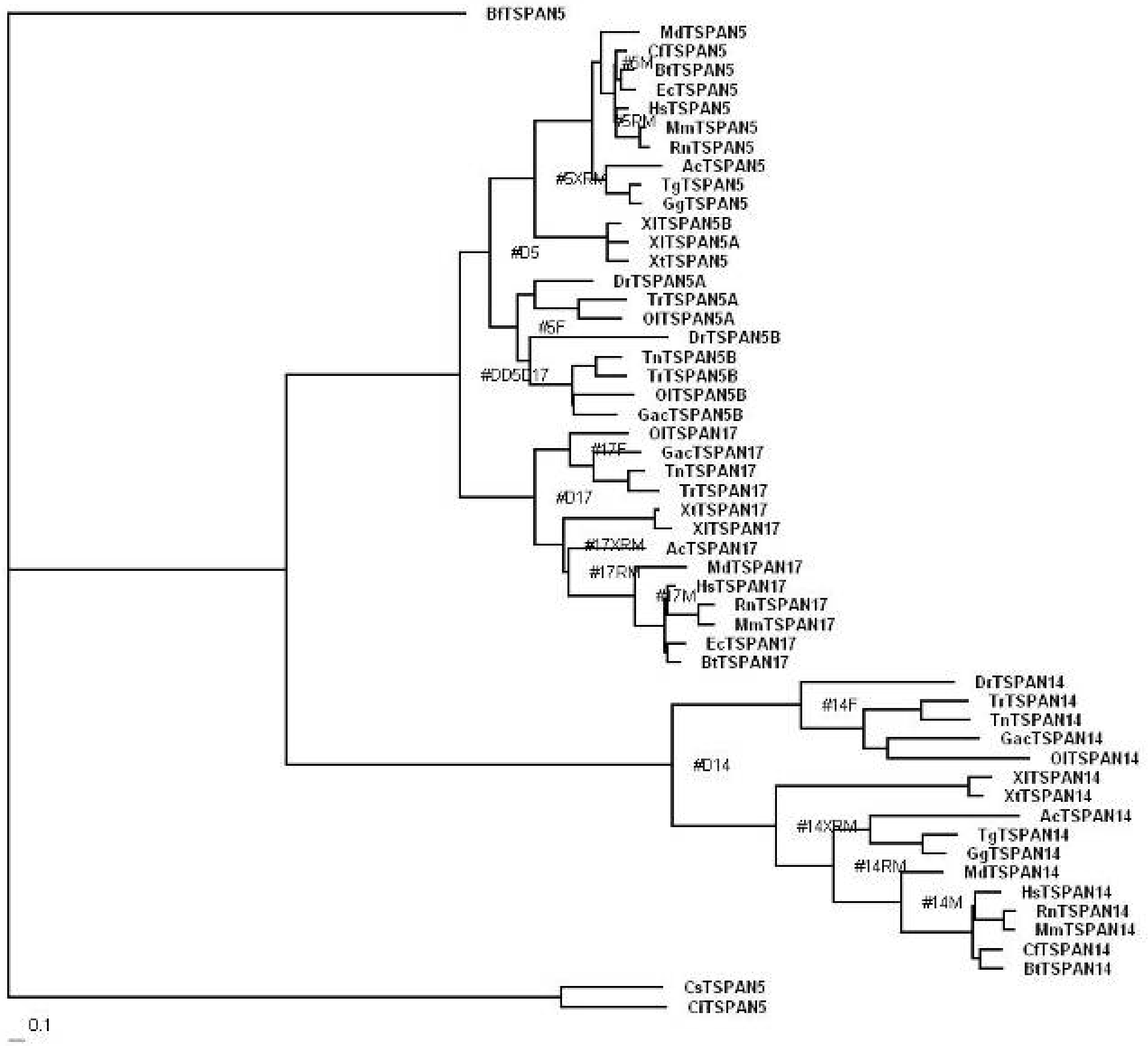

Figure S11. One-ratio tree of TSPAN5 family, used for testing positive selection. The branches for testing are labeled.

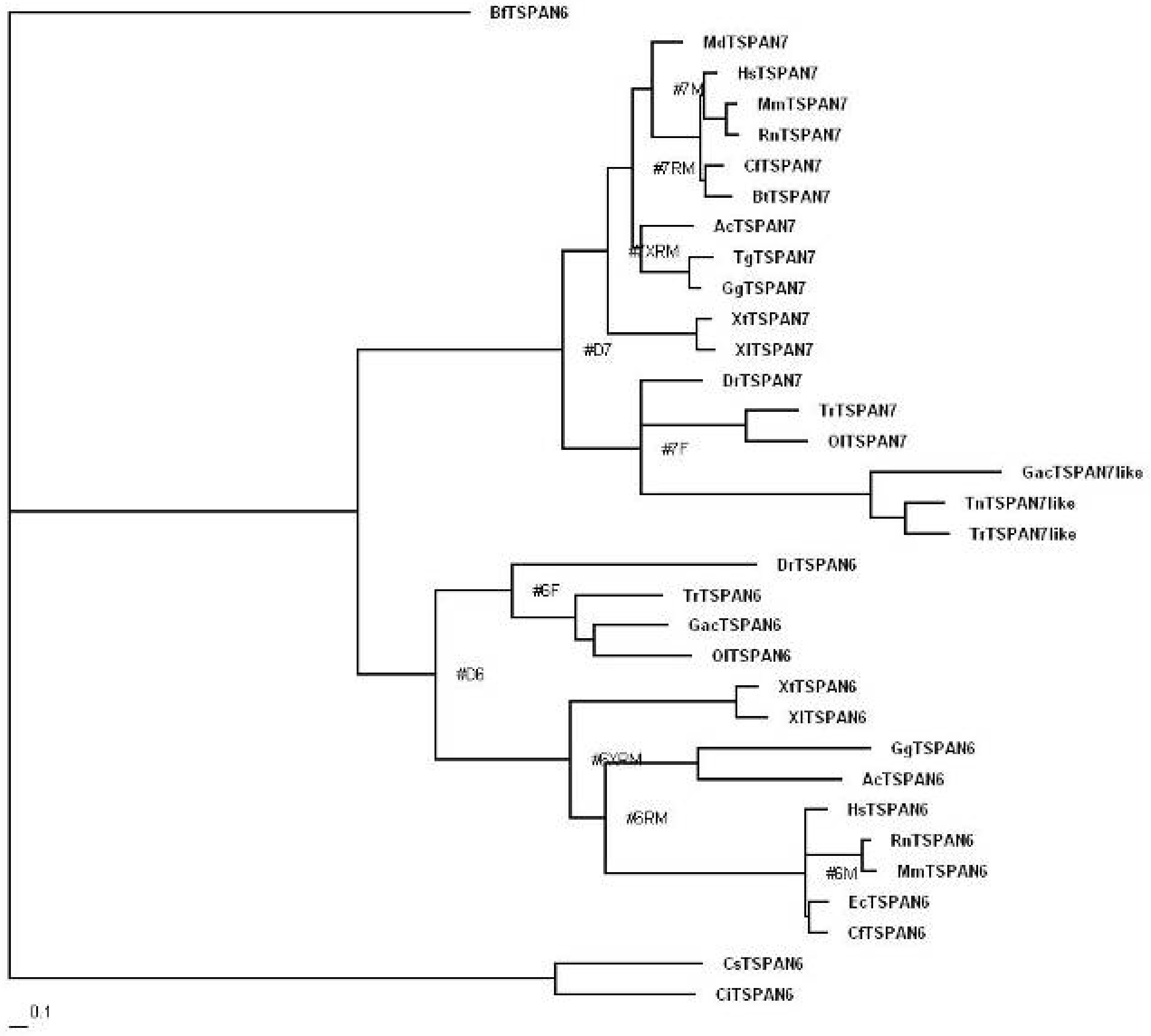

Figure S12. One-ratio tree of TSPAN6 family, used for testing positive selection. The branches for testing are labeled.

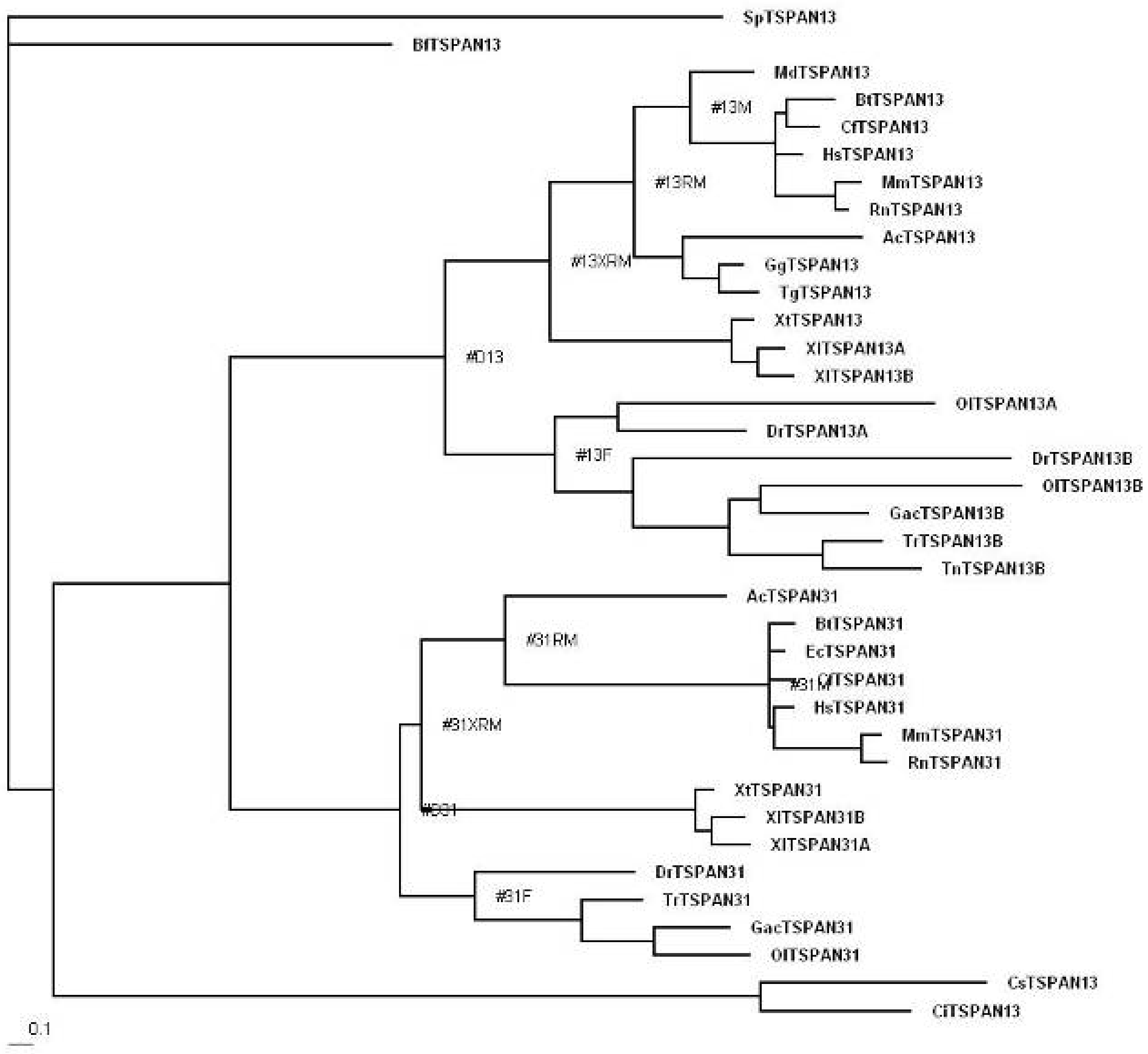

Figure S13. One-ratio tree of TSPAN13 family, used for testing positive selection. The branches for testing are labeled.

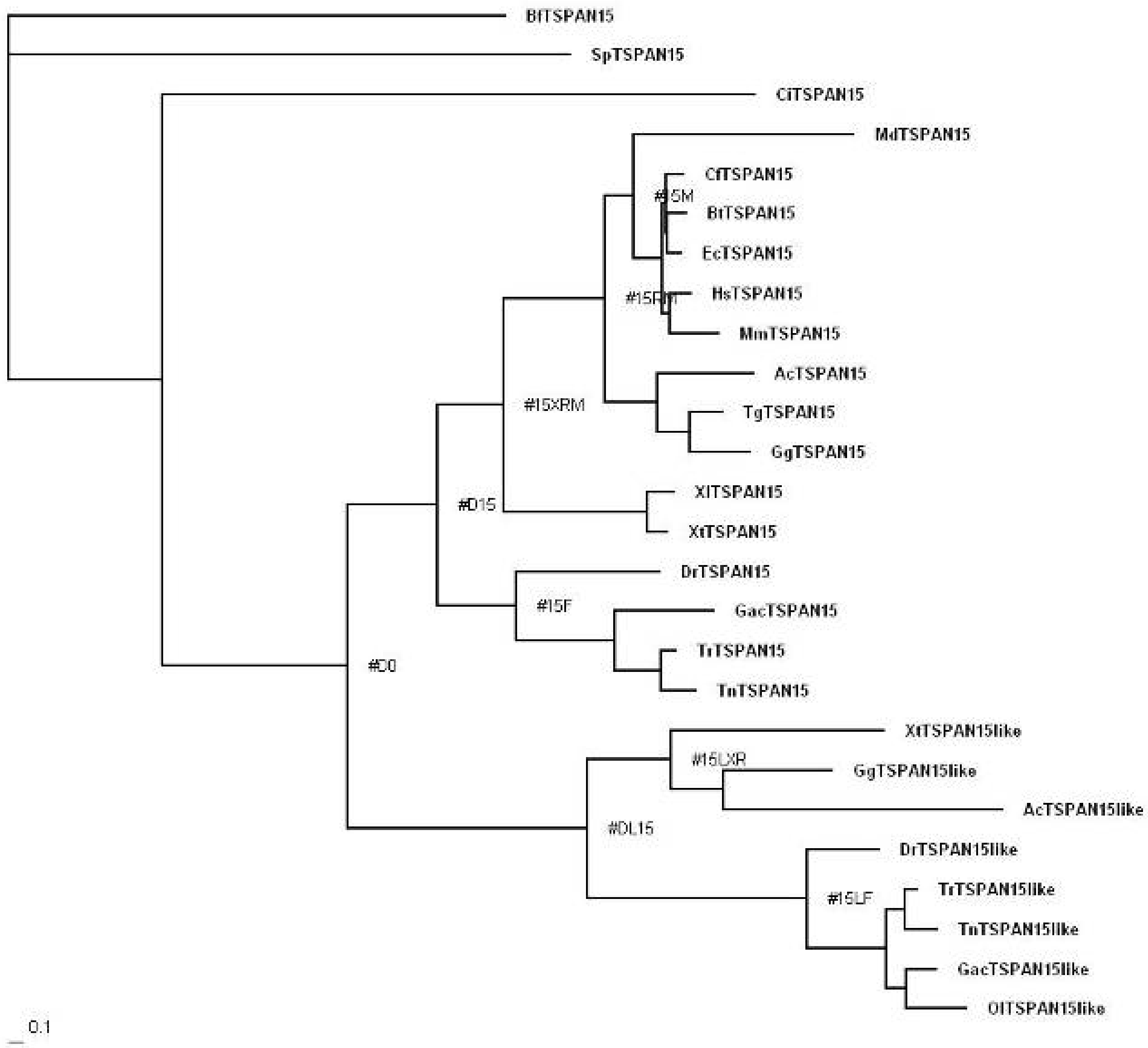

Figure S14. One-ratio tree of TSPAN15 family, used for testing positive selection. The branches for testing are labeled.

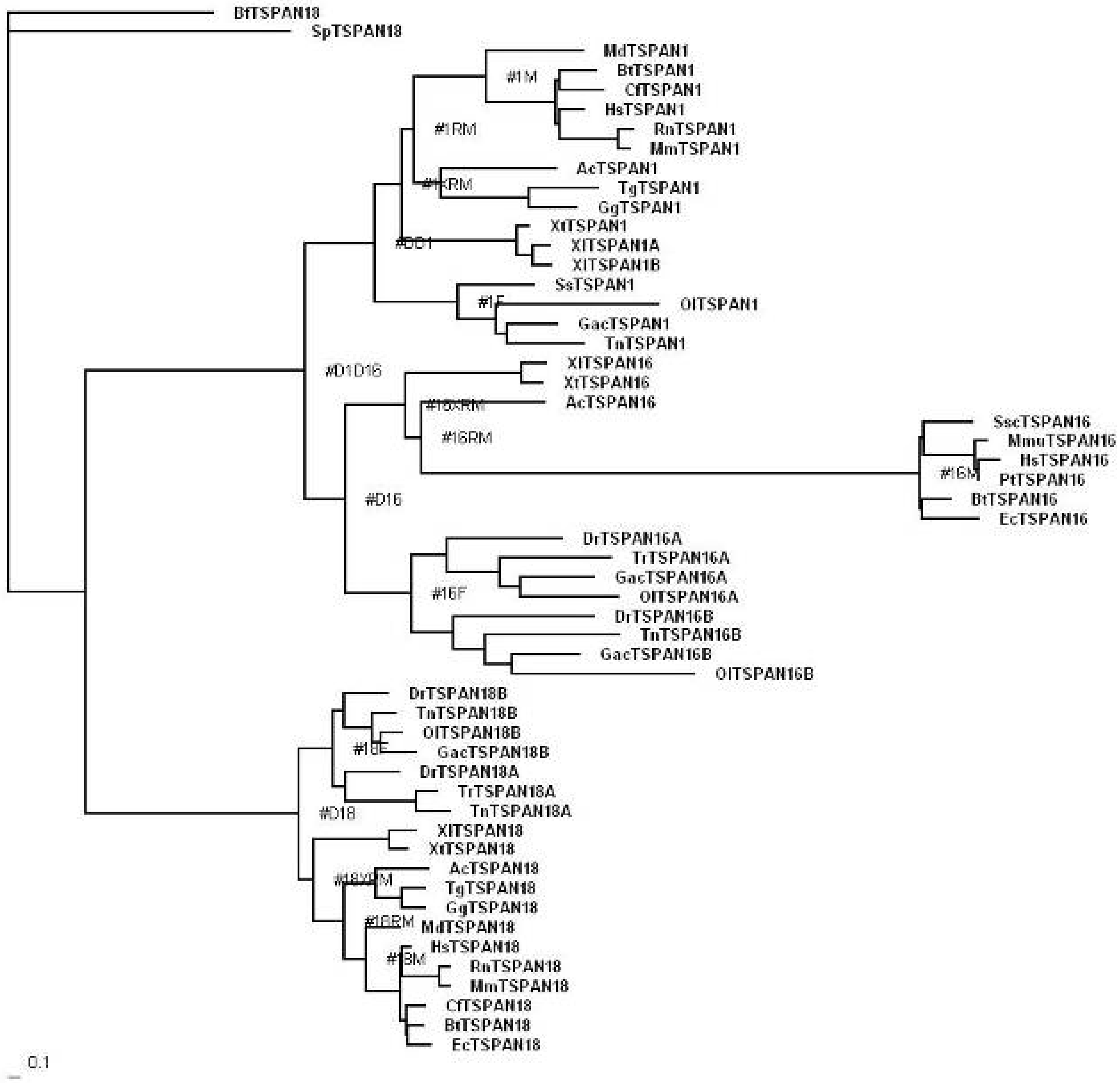

Figure S15. One-ratio tree of TSPAN1 family, used for testing positive selection. The branches for testing are labeled.

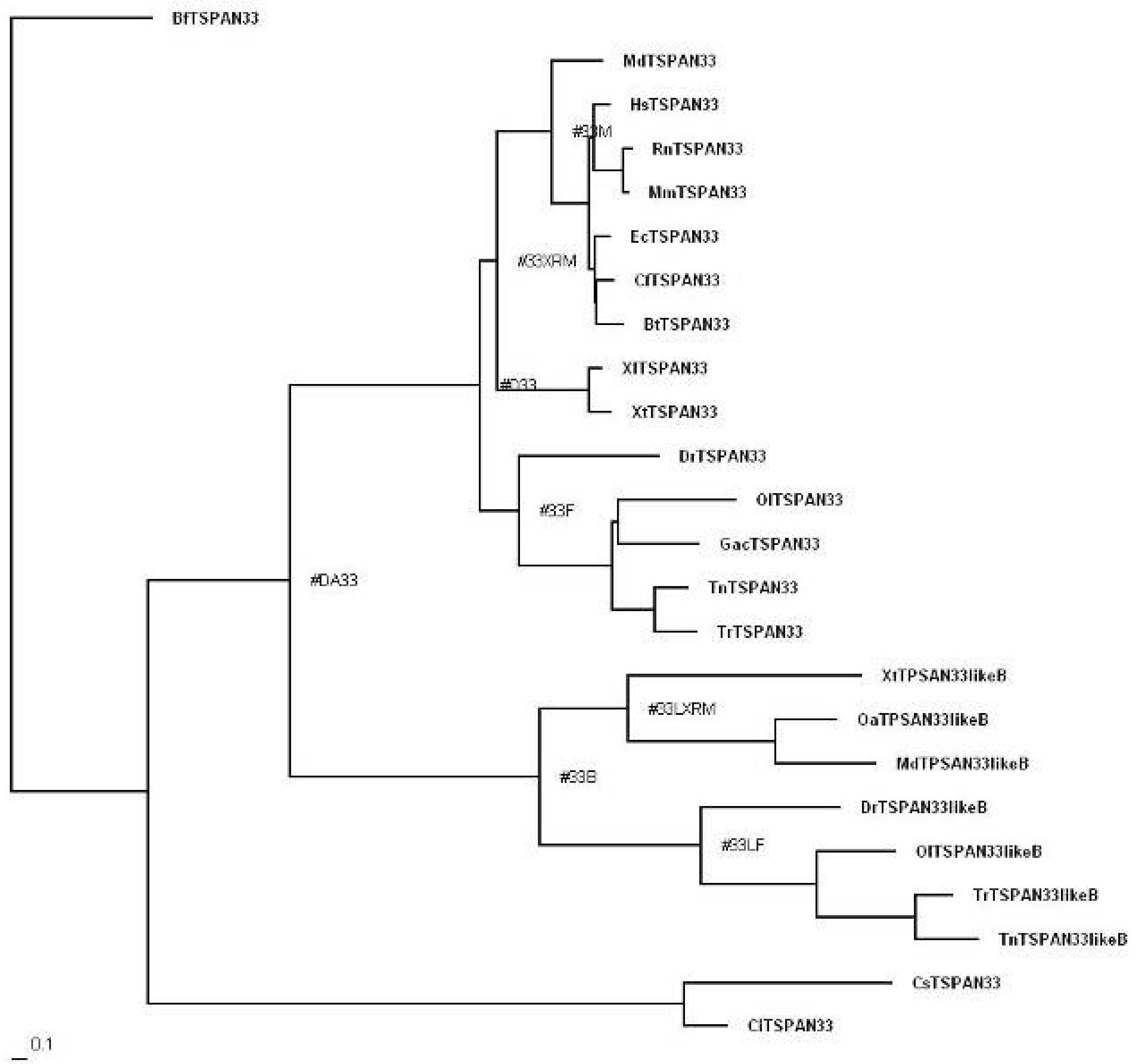

Figure S16. One-ratio tree of TSPAN33 family, used for testing positive selection. The branches for testing are labeled.

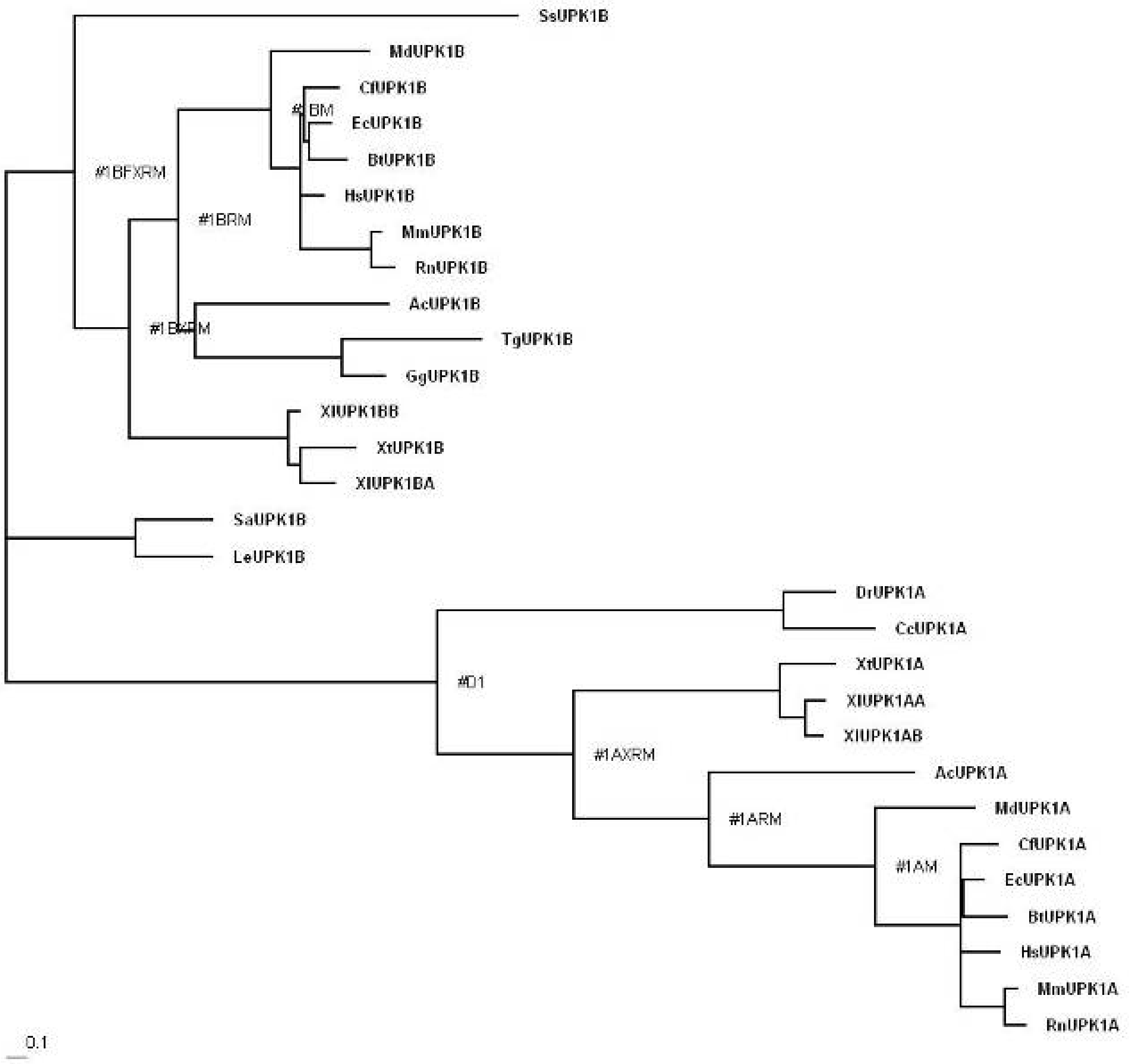

Figure S17. One-ratio tree of UPK1 family, used for testing positive selection. The branches for testing are labeled.

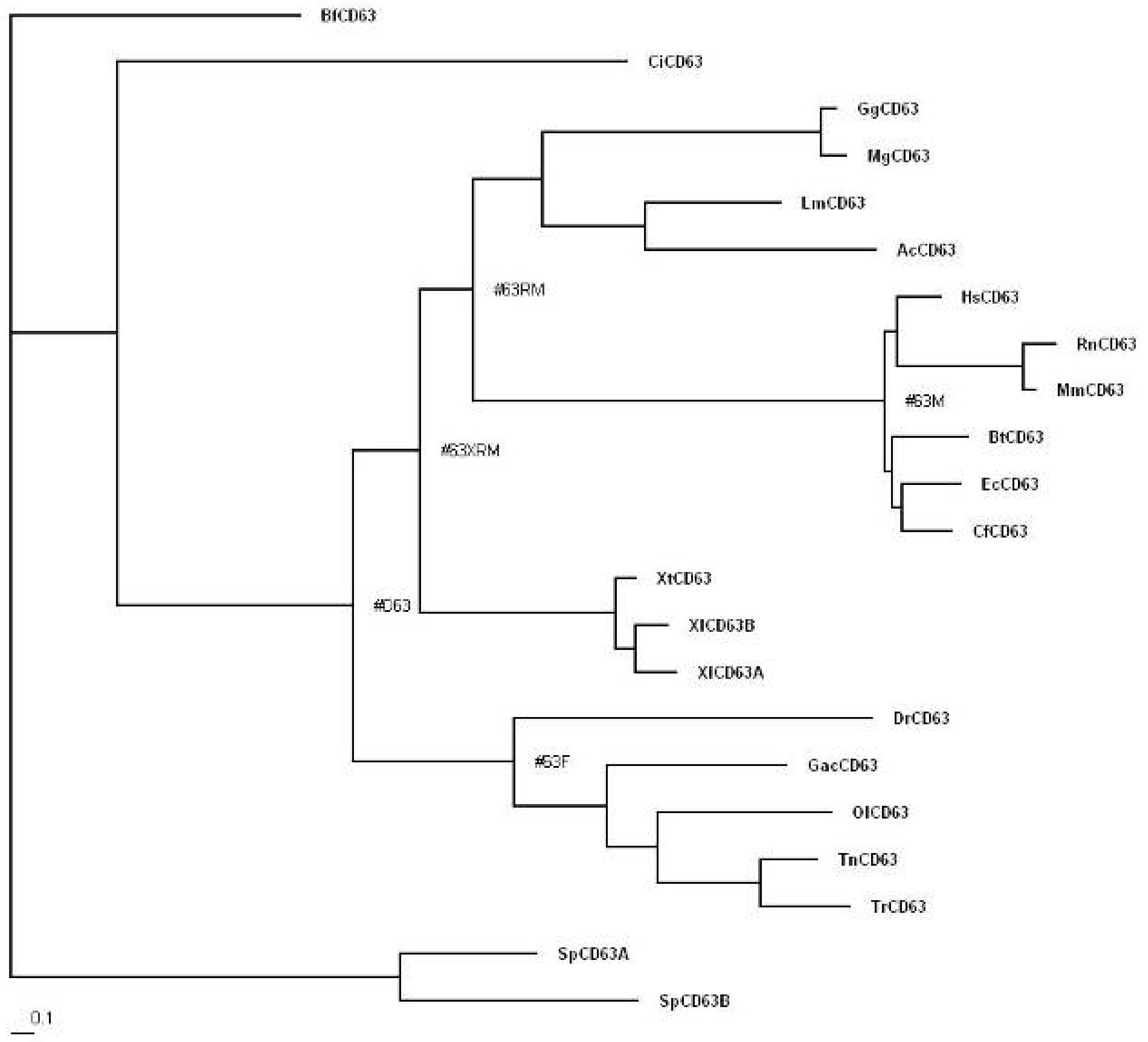

Figure S18. One-ratio tree of CD63 family, used for testing positive selection. The branches for testing are labeled.

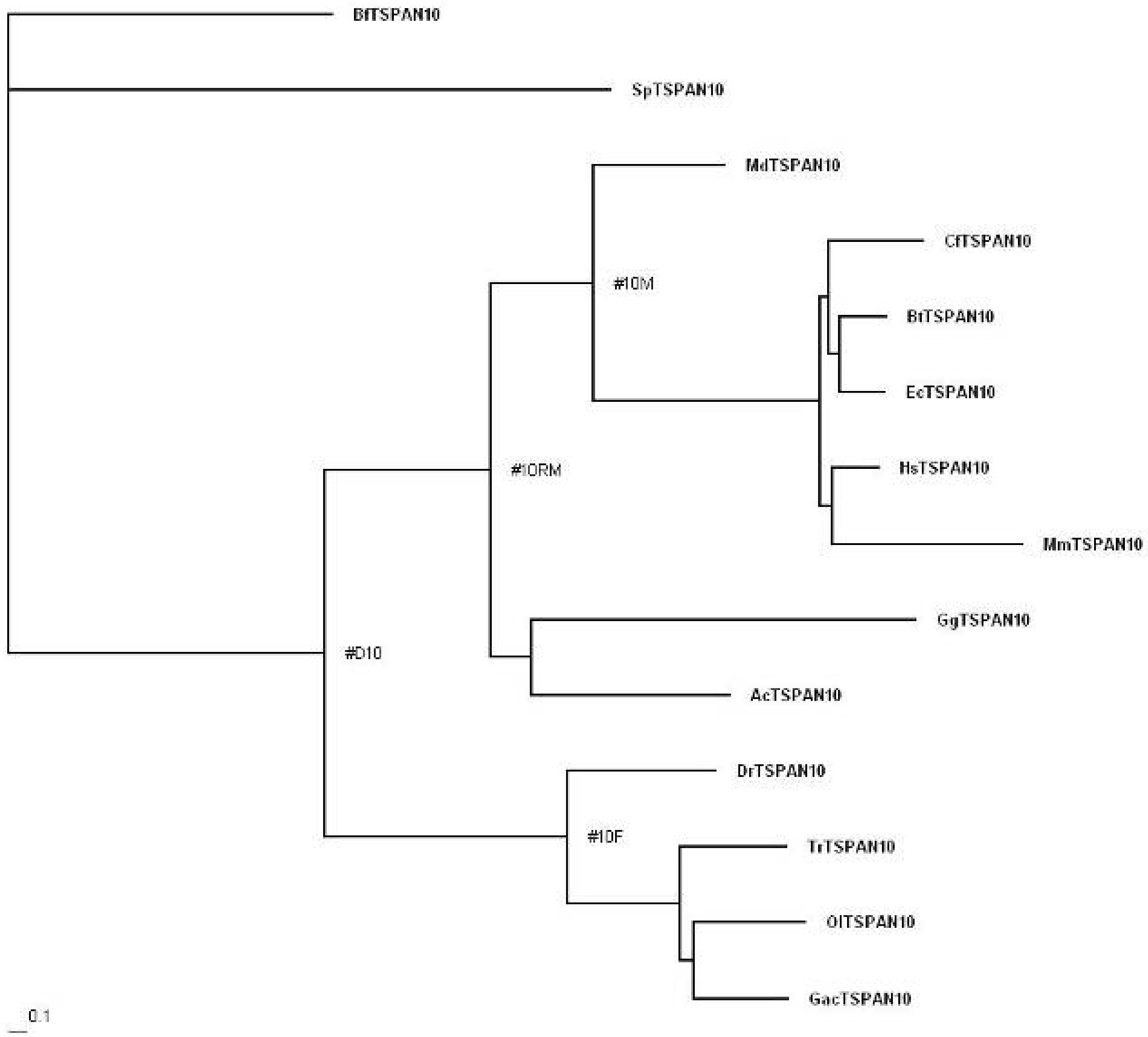

Figure S19. One-ratio tree of TSPAN10 family, used for testing positive selection. The branches for testing are labeled.

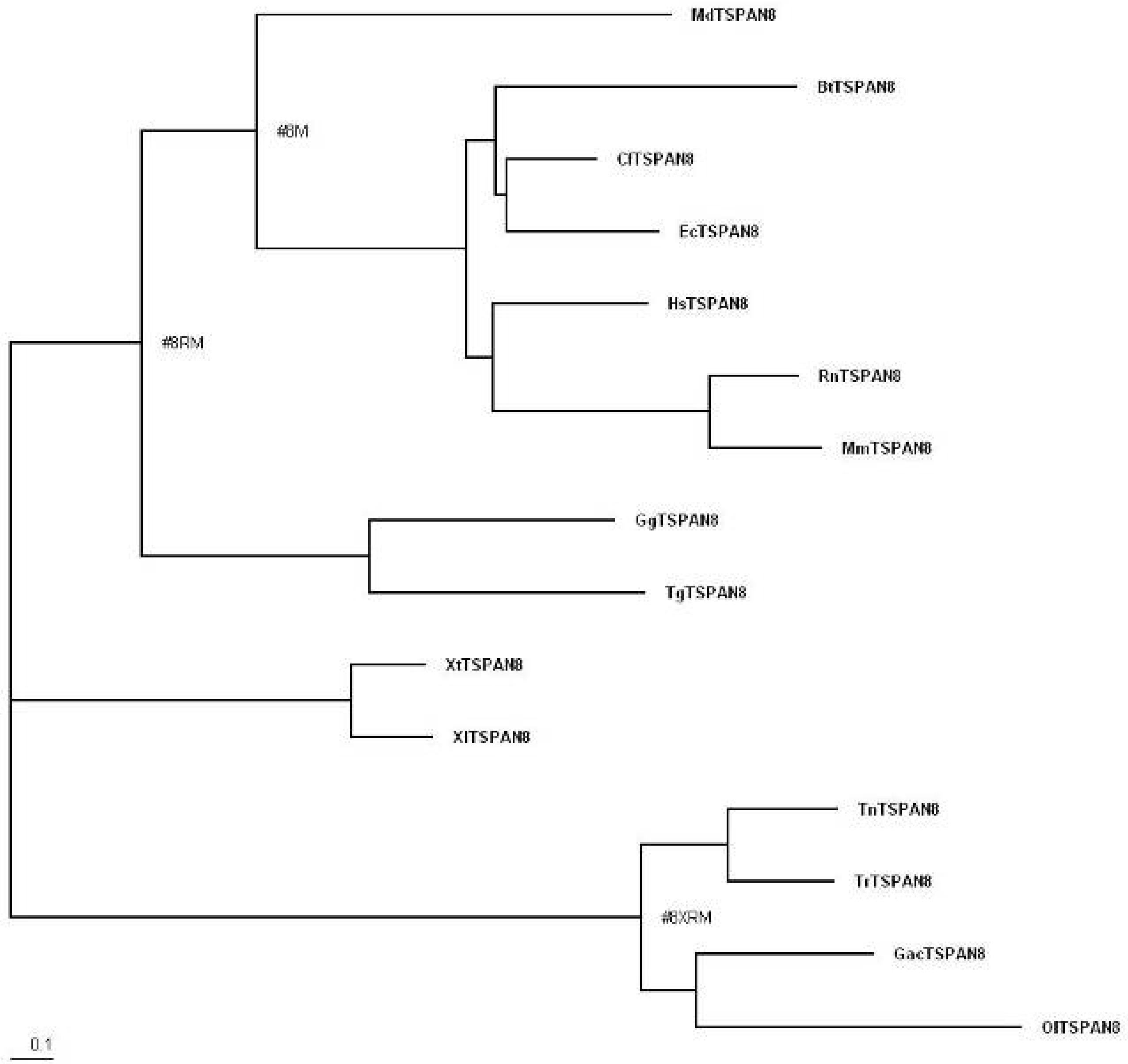

Figure S20. One-ratio tree of TSPAN8 family, used for testing positive selection. The branches for testing are labeled.

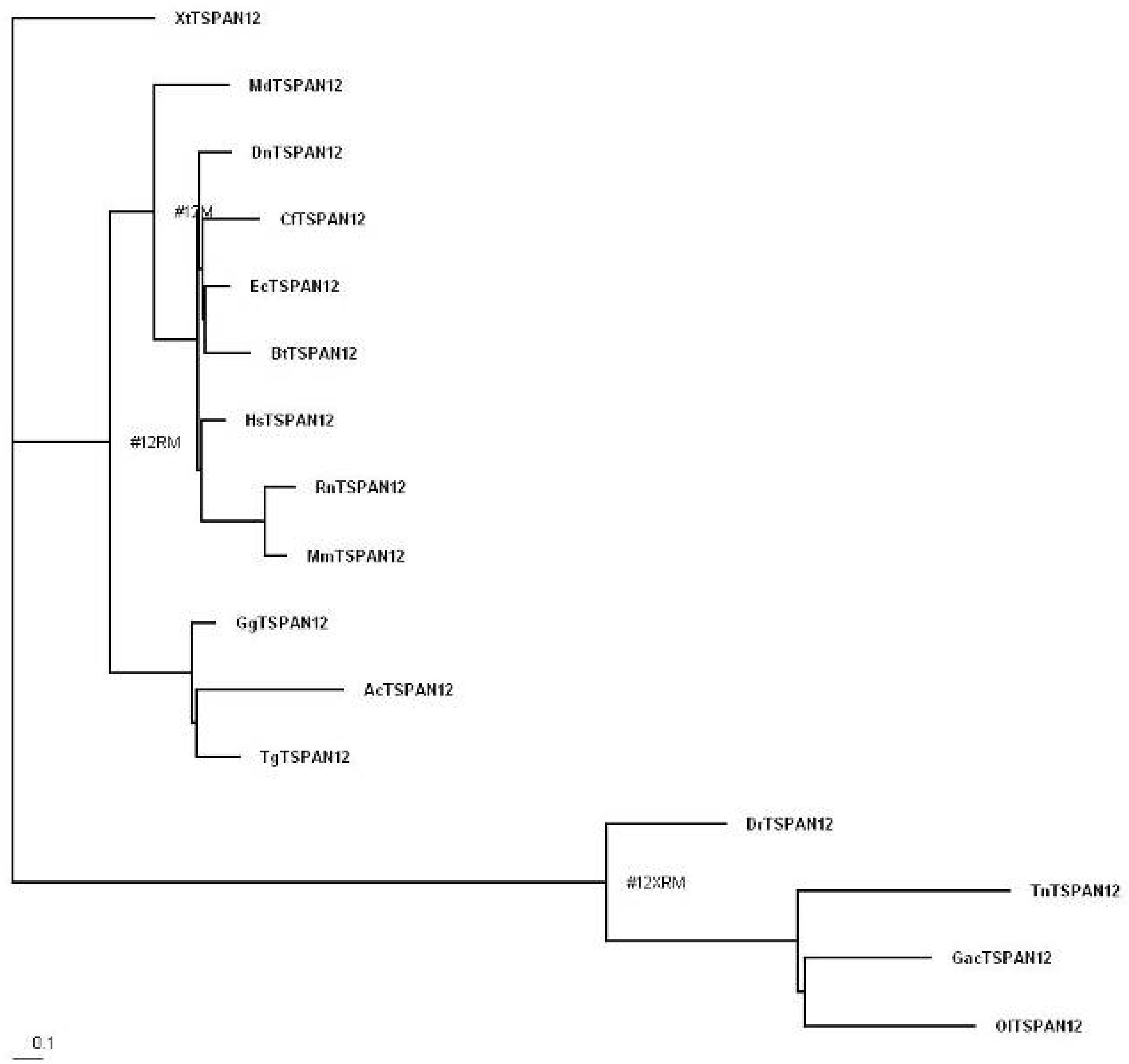

Figure S21. One-ratio tree of TSPAN12 family, used for testing positive selection. The branches for testing are labeled.

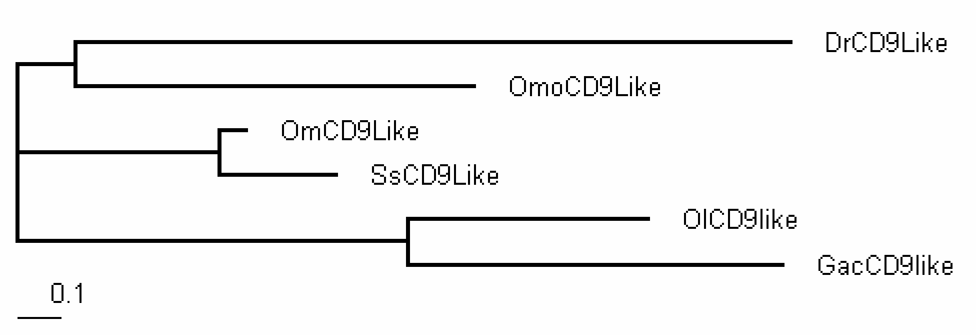

Figure S22. One-ratio tree of CD9-like family, used for testing positive selection.

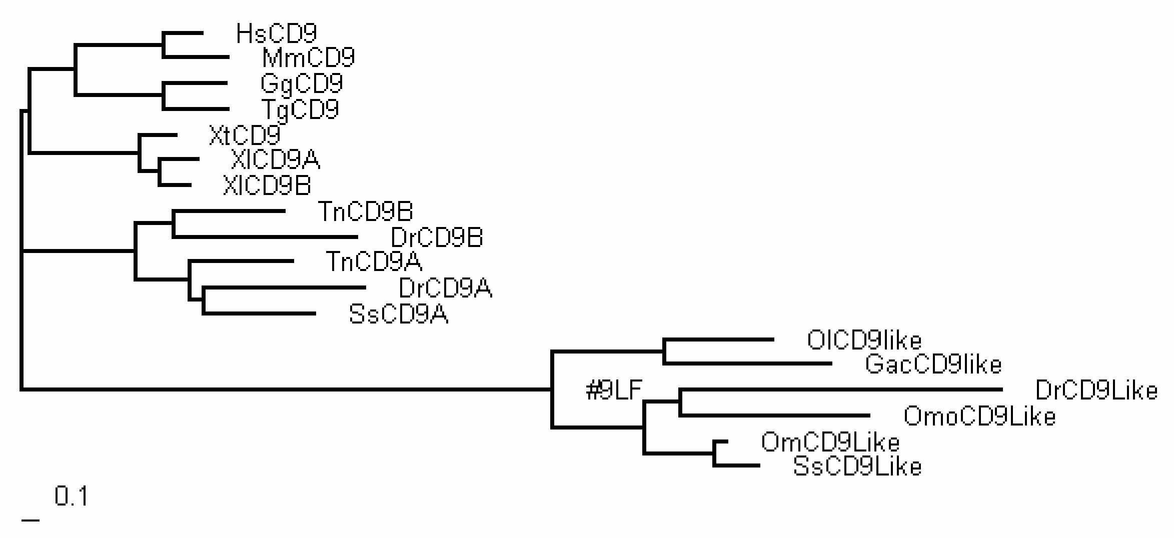

Figure S23. One-ratio tree of CD9-like and CD9, used for testing positive selection. The branches for testing are labeled.

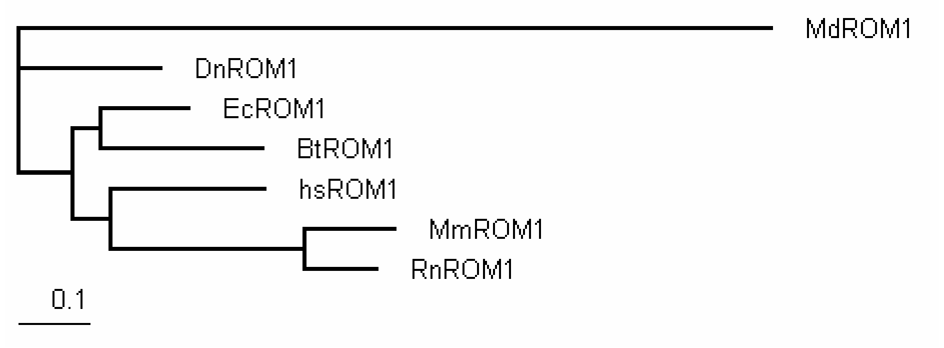

Figure S24. One-ratio tree of mammal ROM1, used for testing positive selection.

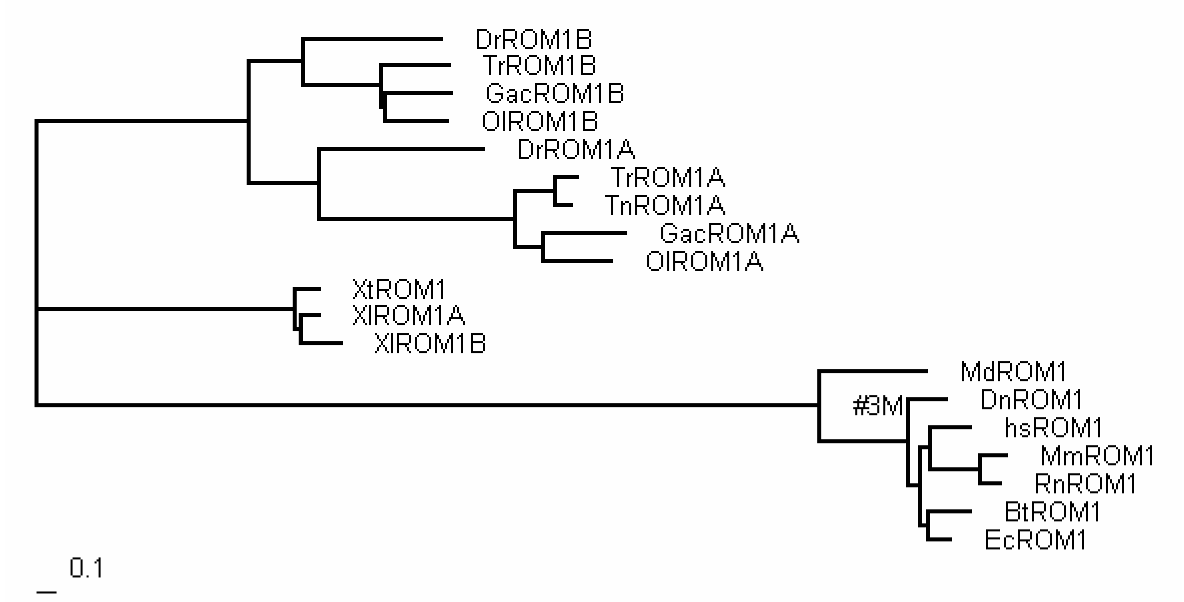

Figure S25. One-ratio tree of ROM1 family, used for testing positive selection. The branches for testing are labeled.

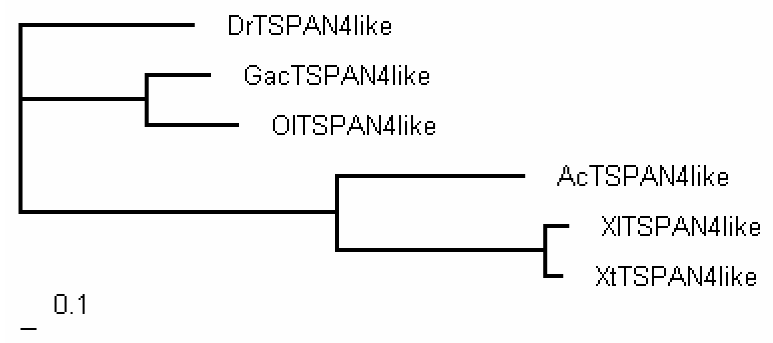

Figure S26. One-ratio tree of TSPAN4-like family, used for testing positive selection.

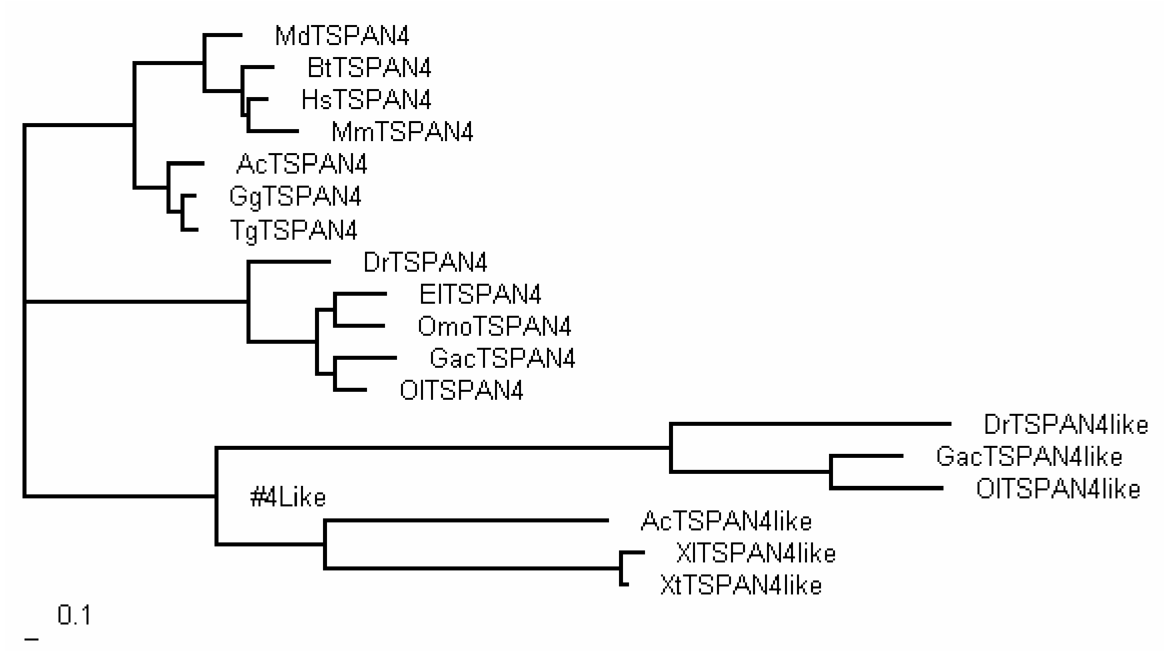

Figure S27. One-ratio tree of TSPAN4 and TSPAN4-like, used for testing positive selection. The branches for testing are labeled.

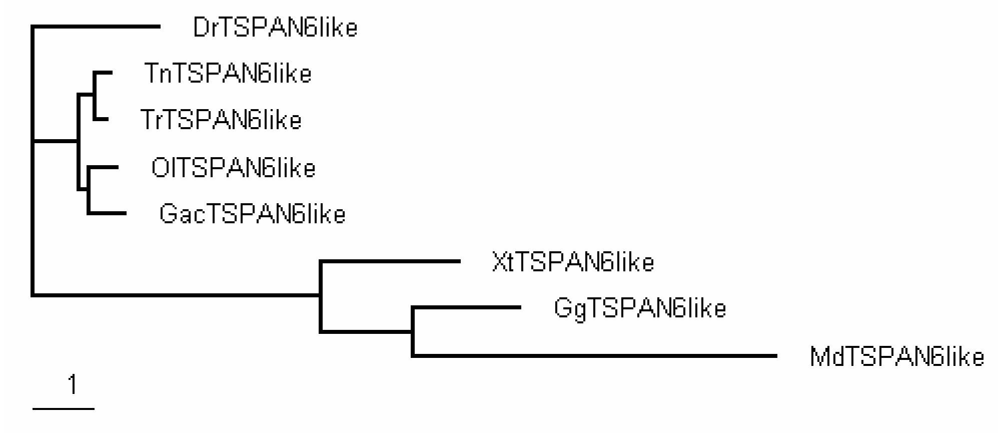

Figure S28. One-ratio tree of TSPAN6-like (full-length), used for testing positive selection.

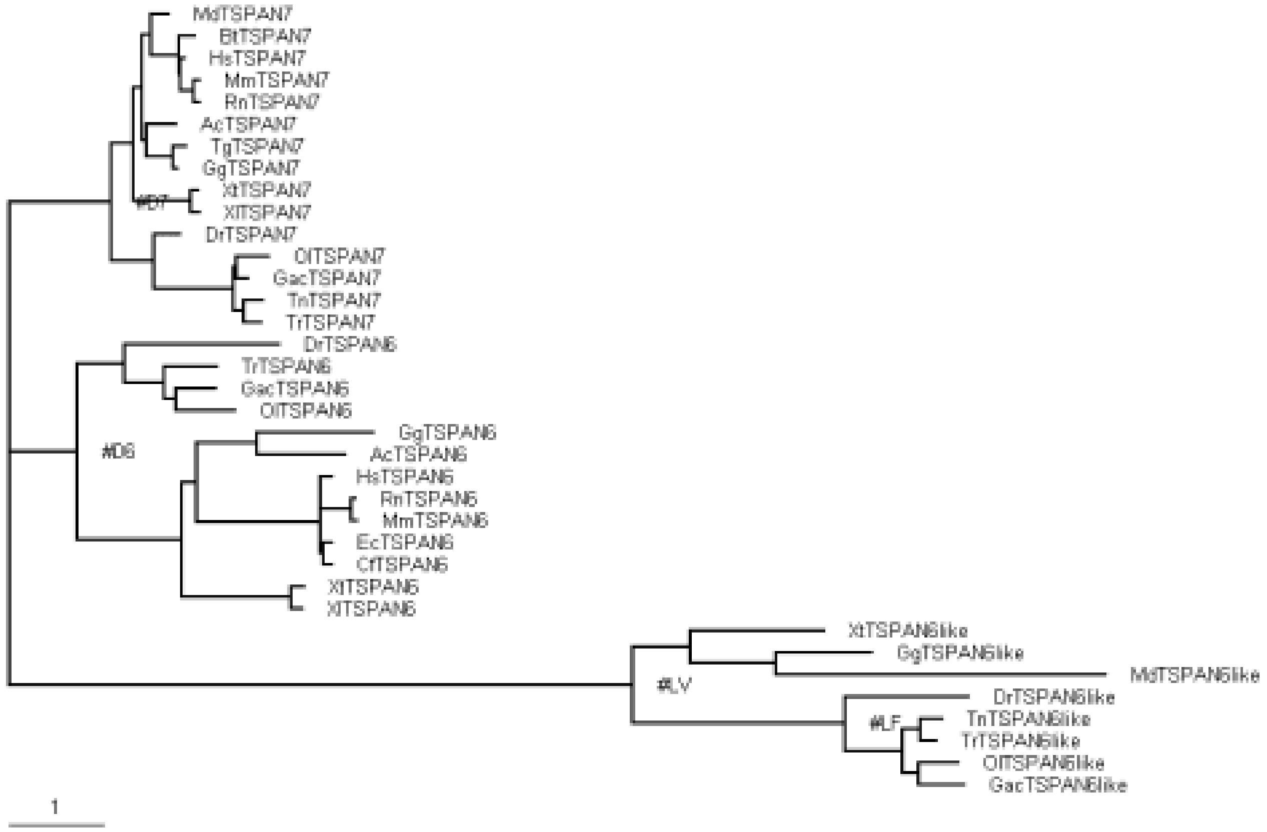

Figure S29. One-ratio tree of TSPAN6 family and TSPAN6-like, used for testing positive selection. The branches for testing are labeled.

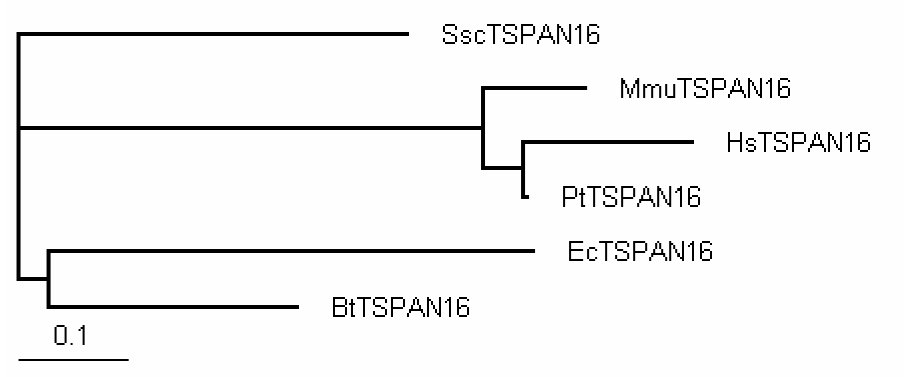

Figure S30. One-ratio tree of mammal TSPAN16 family, used for testing positive selection.

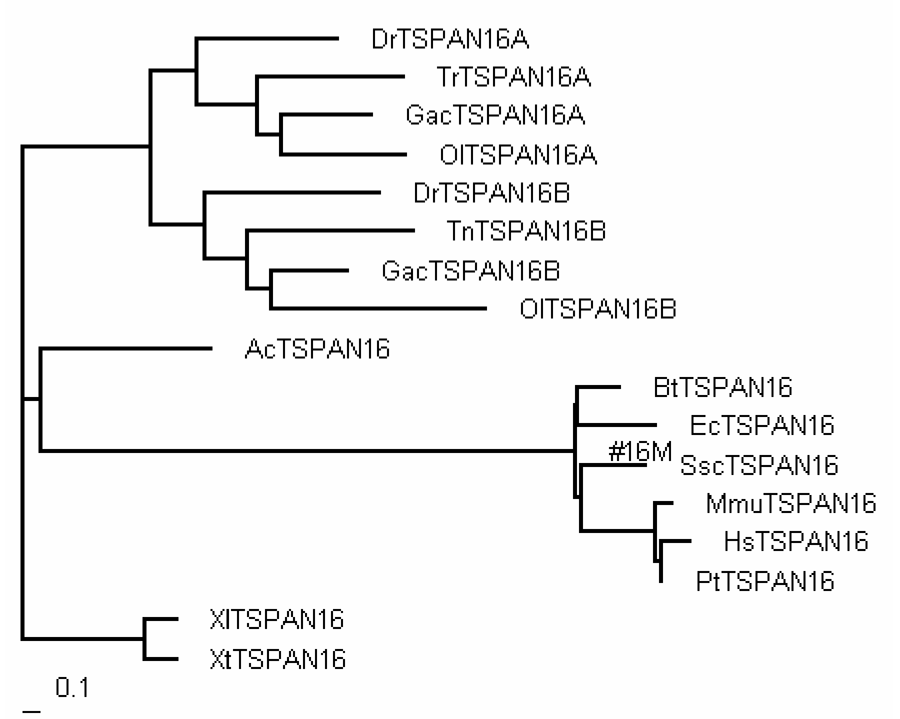

Figure S31. One-ratio tree of TSPAN16 family, used for testing positive selection. The branches for testing are labeled.

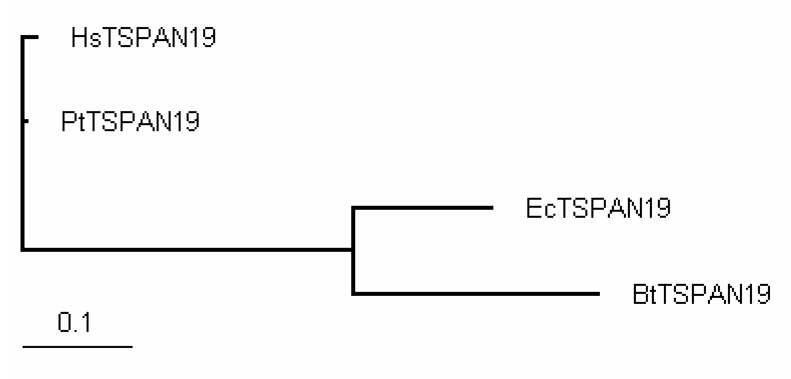

Figure S32. One-ratio tree of TSPAN19 family, used for testing positive selection.

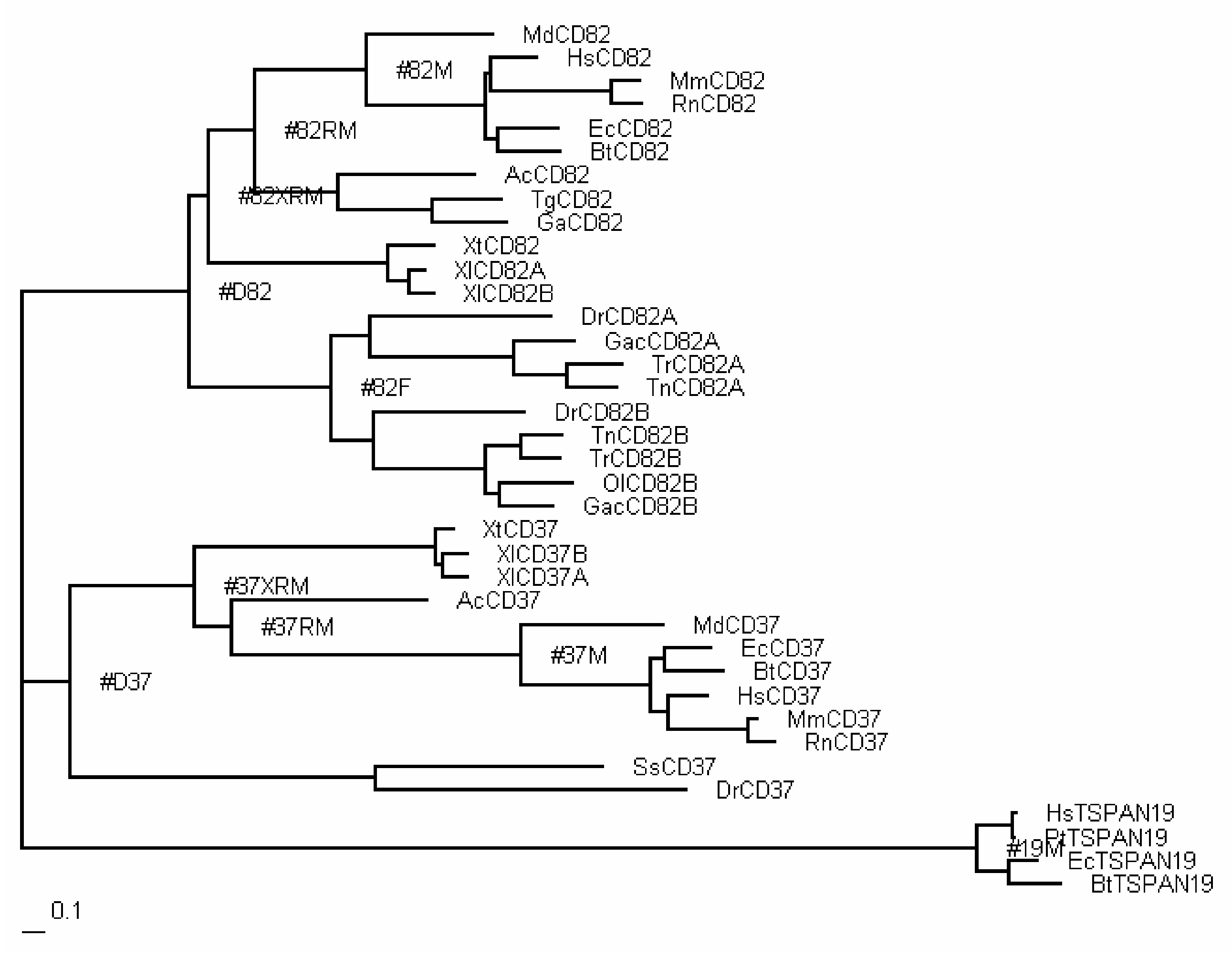

Figure S33. One-ratio tree of CD37 family (including TSPAN19), used for testing positive selection. The branches for testing are labeled.

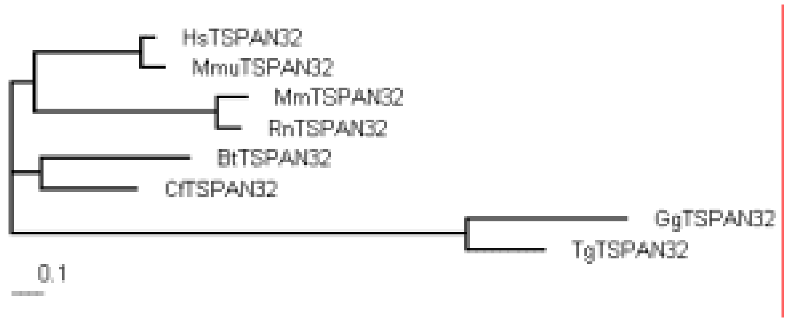

Figure S34. One-ratio tree of TSPAN32 family, used for testing positive selection.

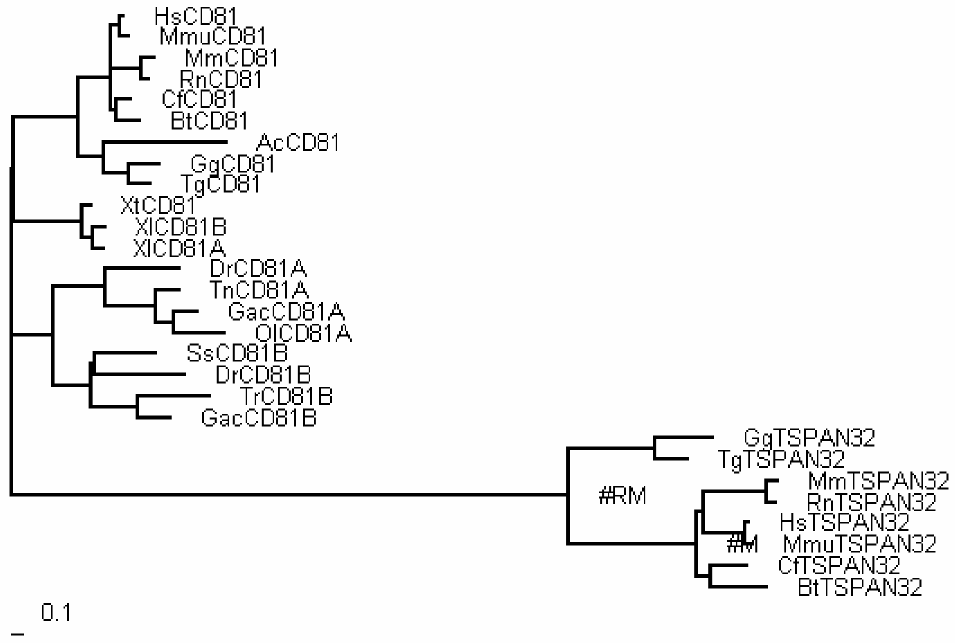

Figure S35. One-ratio tree of CD81 and TSPAN32 family (structure 1), used for testing positive selection. The branches for testing are labeled.

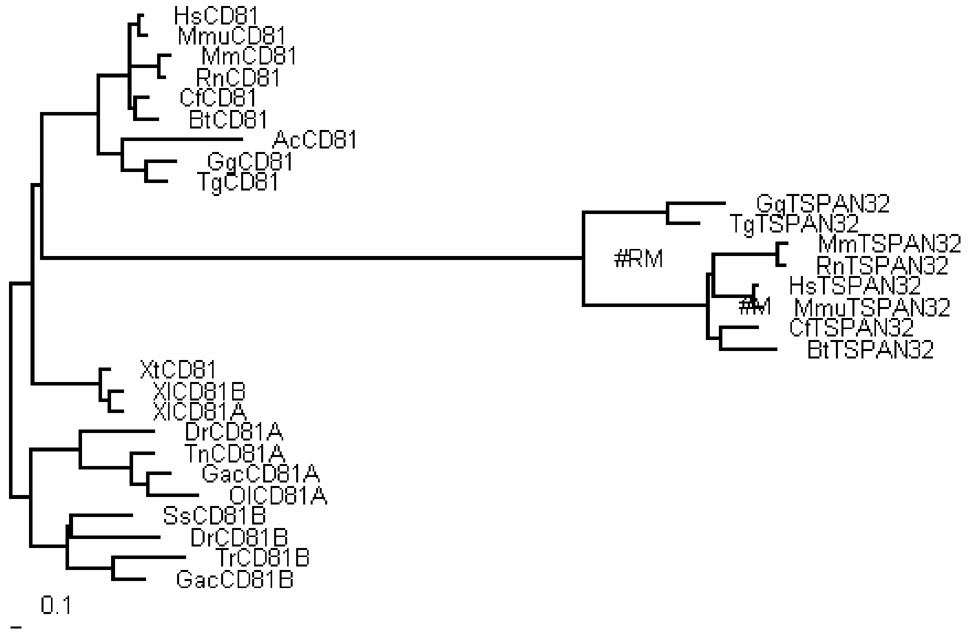

Figure S36. One-ratio tree of CD81 and TSPAN32 family (structure 2), used for testing positive selection. The branches for testing are labeled.

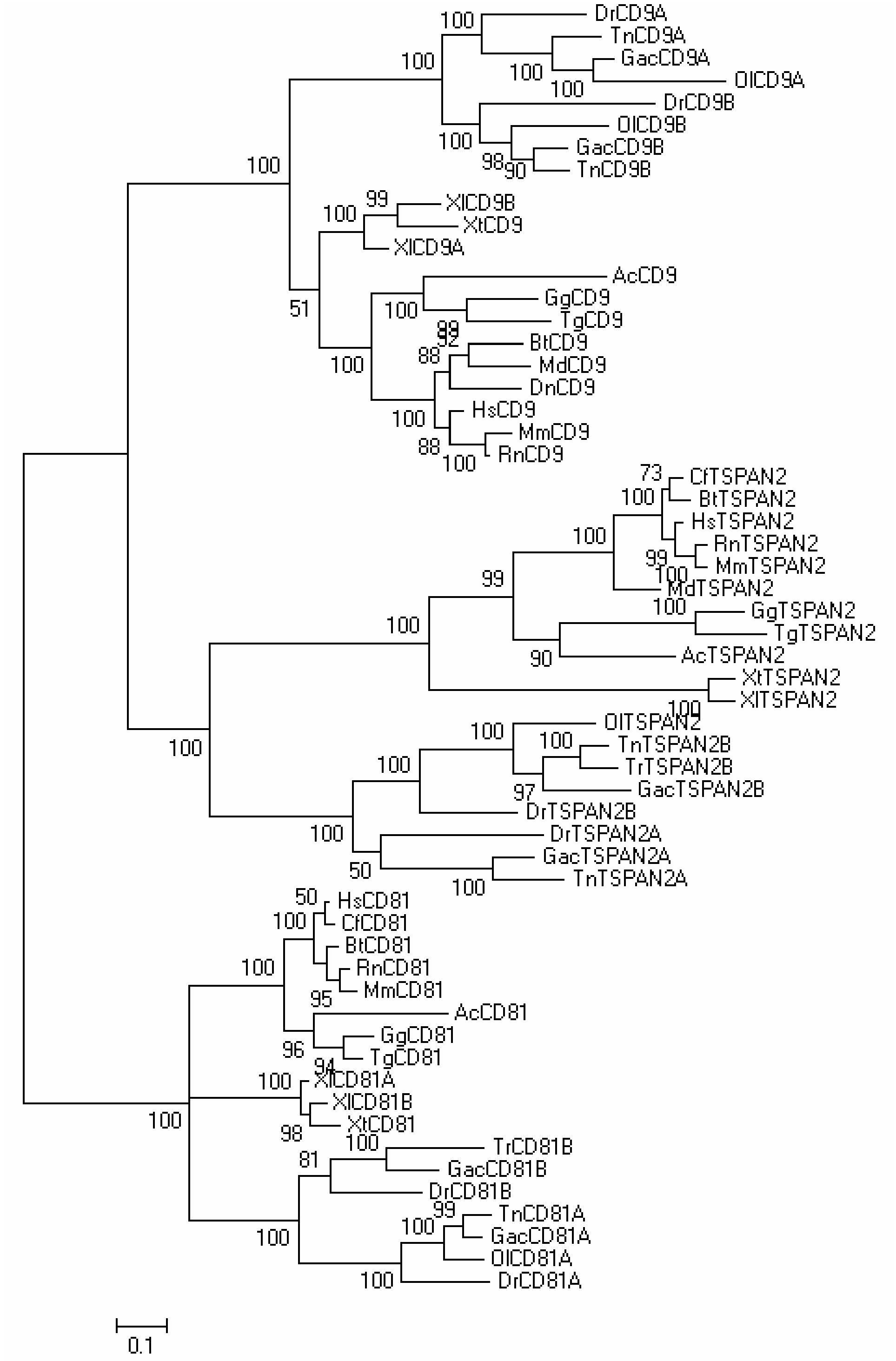

Figure S37. Bayesian protein tree of CD9 family.

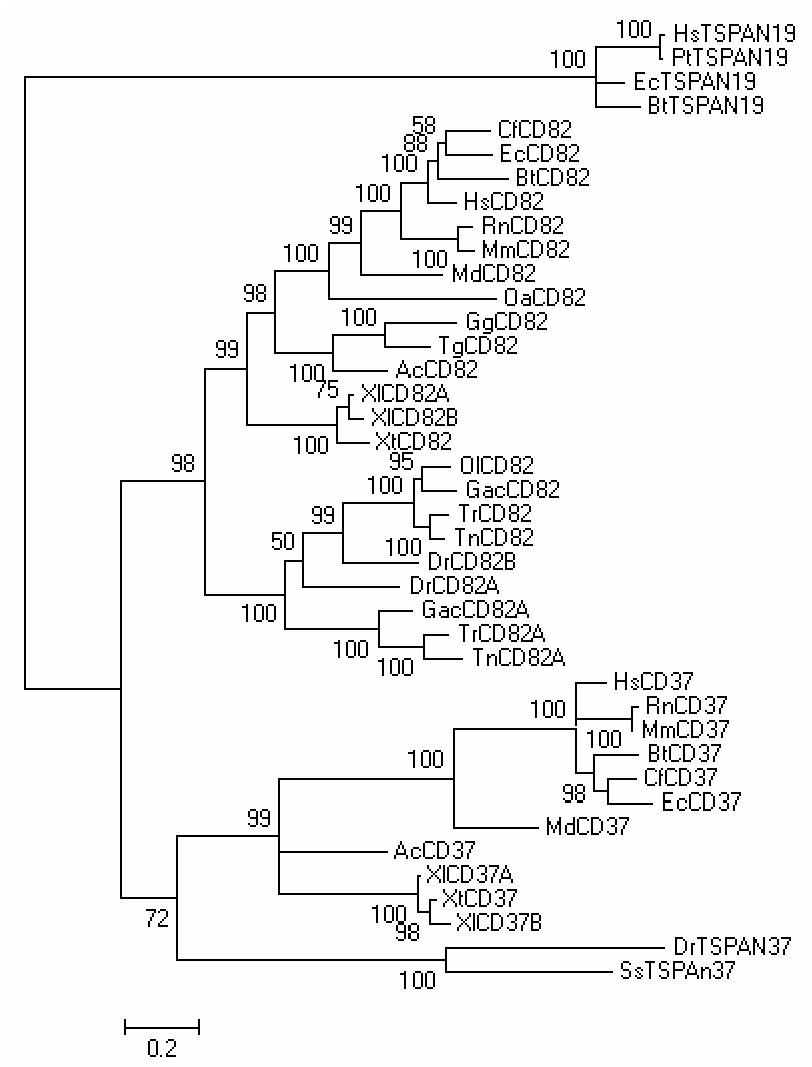

Figure S38. Bayesian protein tree of CD37 family.

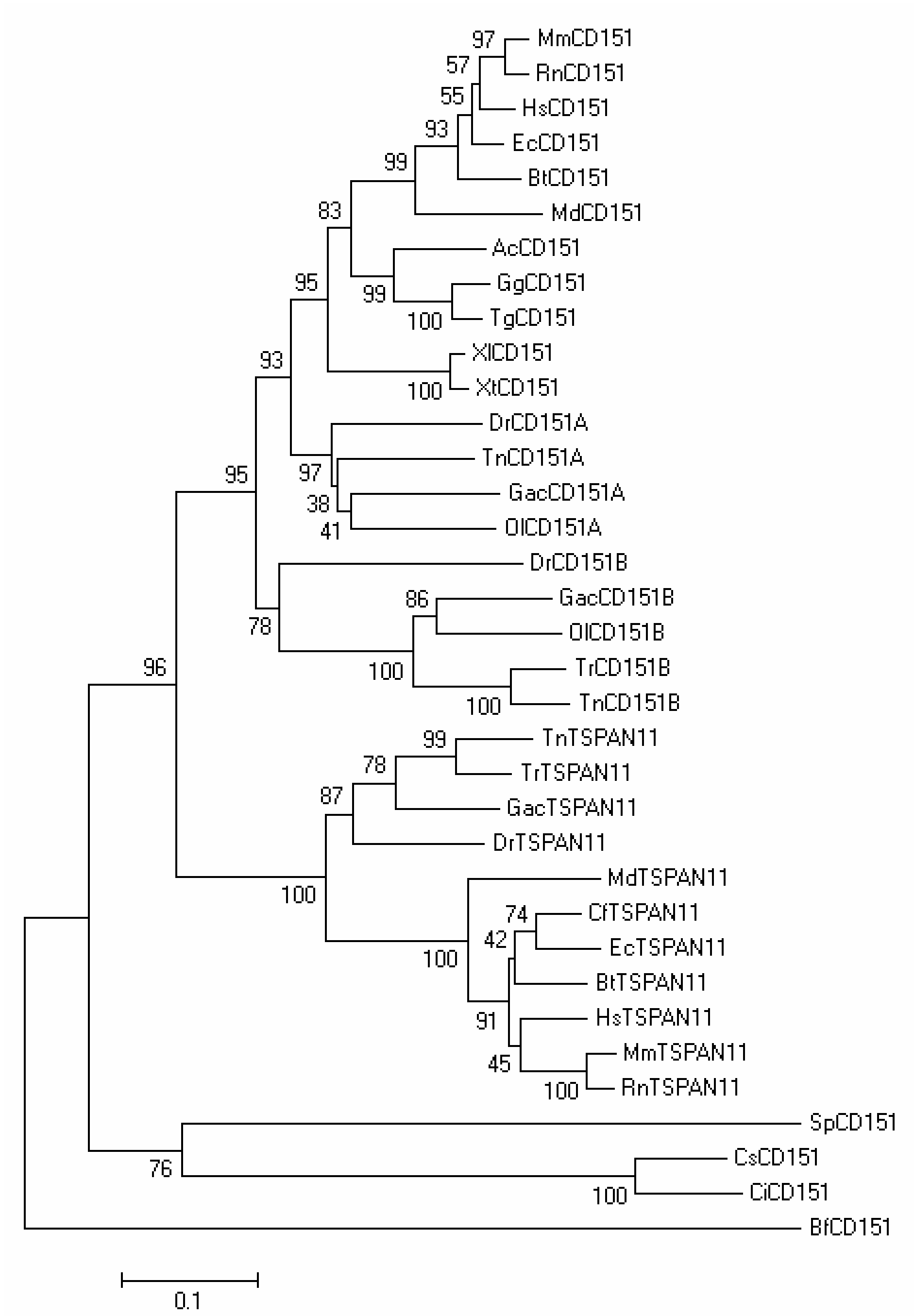

Figure S39. Bayesian protein tree of CD151 family.

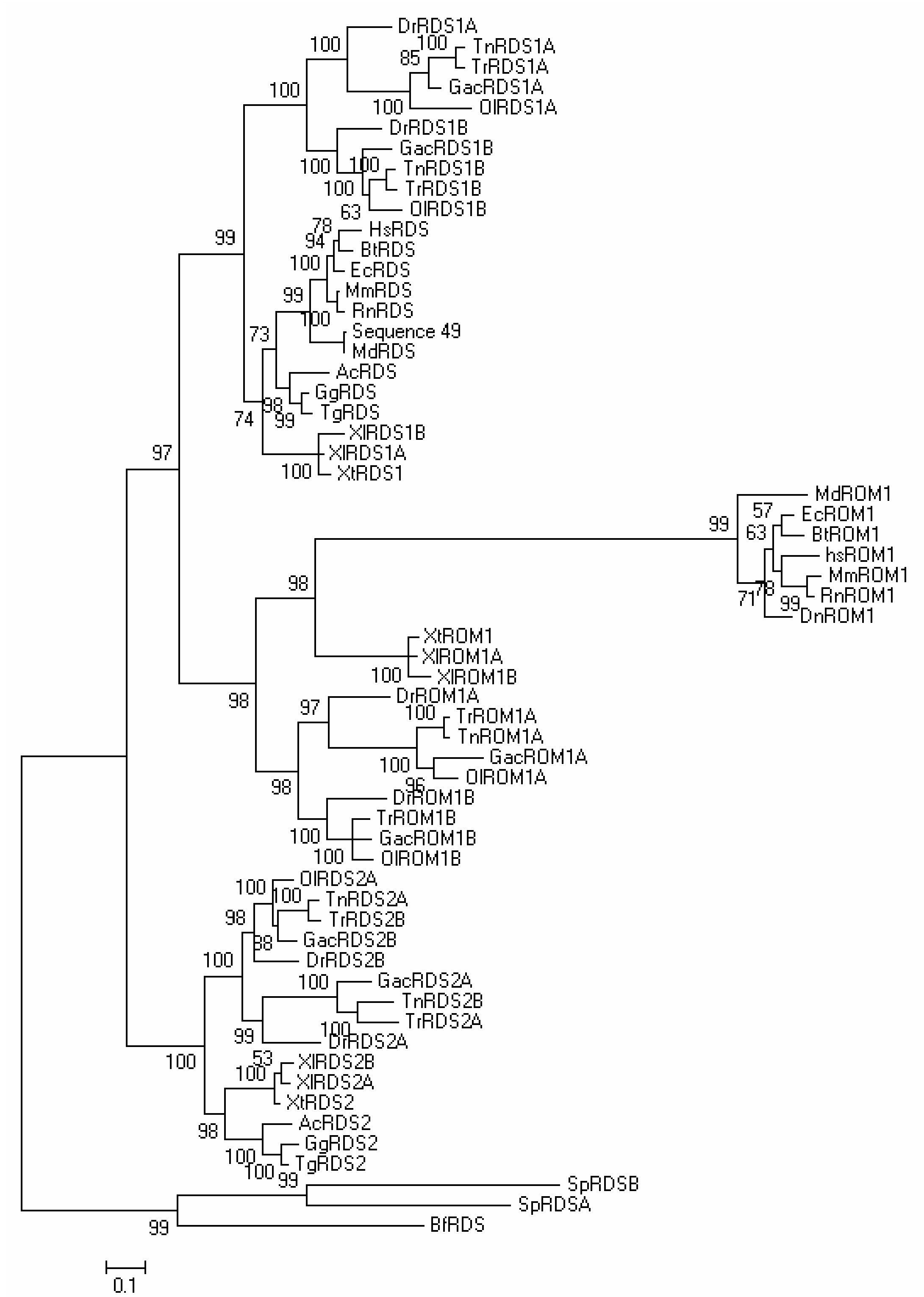

Figure S40. Bayesian protein tree of RDS family.

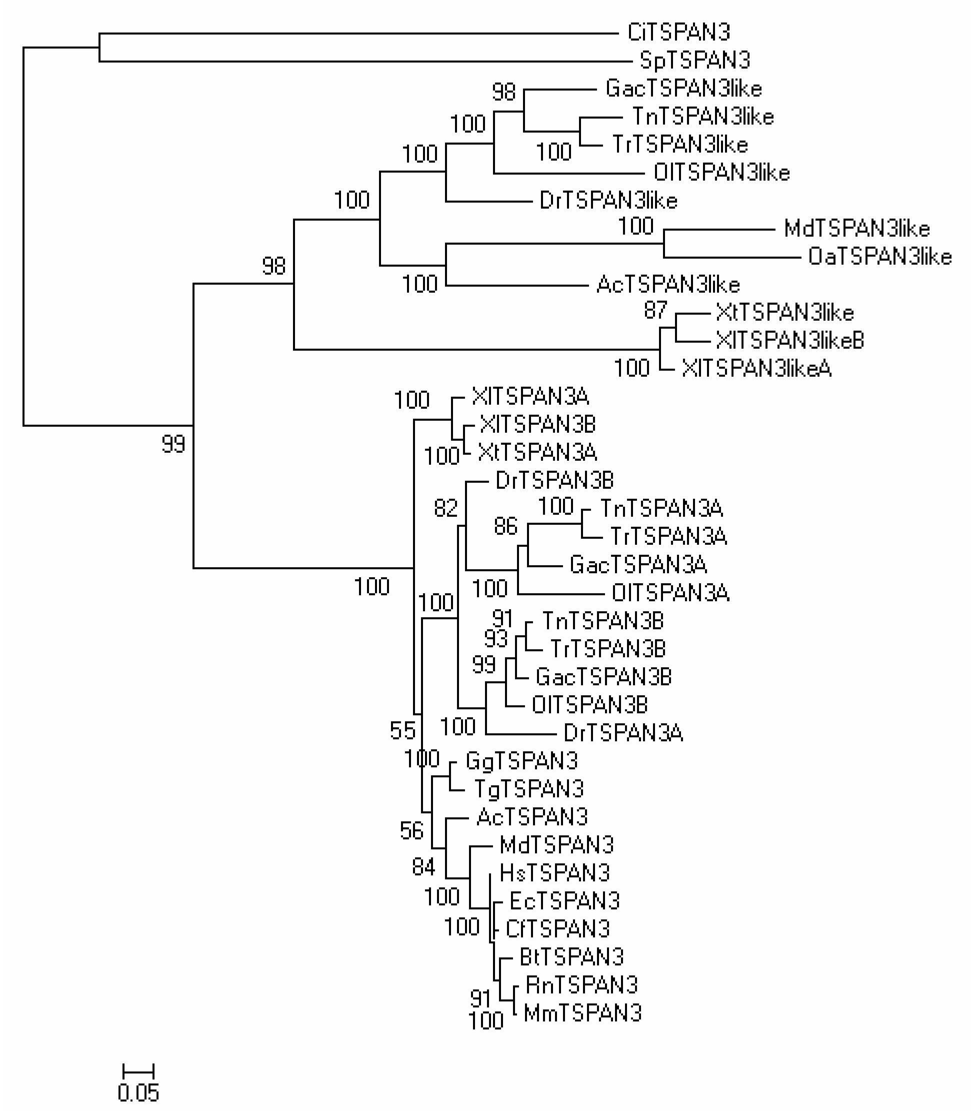

Figure S41. Bayesian protein tree of TSPAN3 family.

Note that xenopus TSPAN3-like violates the species tree, but syntenic information suggests that it is the ortholog of reptile/mammalian TSPAN3-like (CCDC11-RKHD2-TSPAN3-like-ME2).

Note that xenopus TSPAN3 also violates the species tree, but syntenic information suggests that it is the ortholog of mammalian TSPAN3 (ETFA-TSPAN3-PSTPIP1).

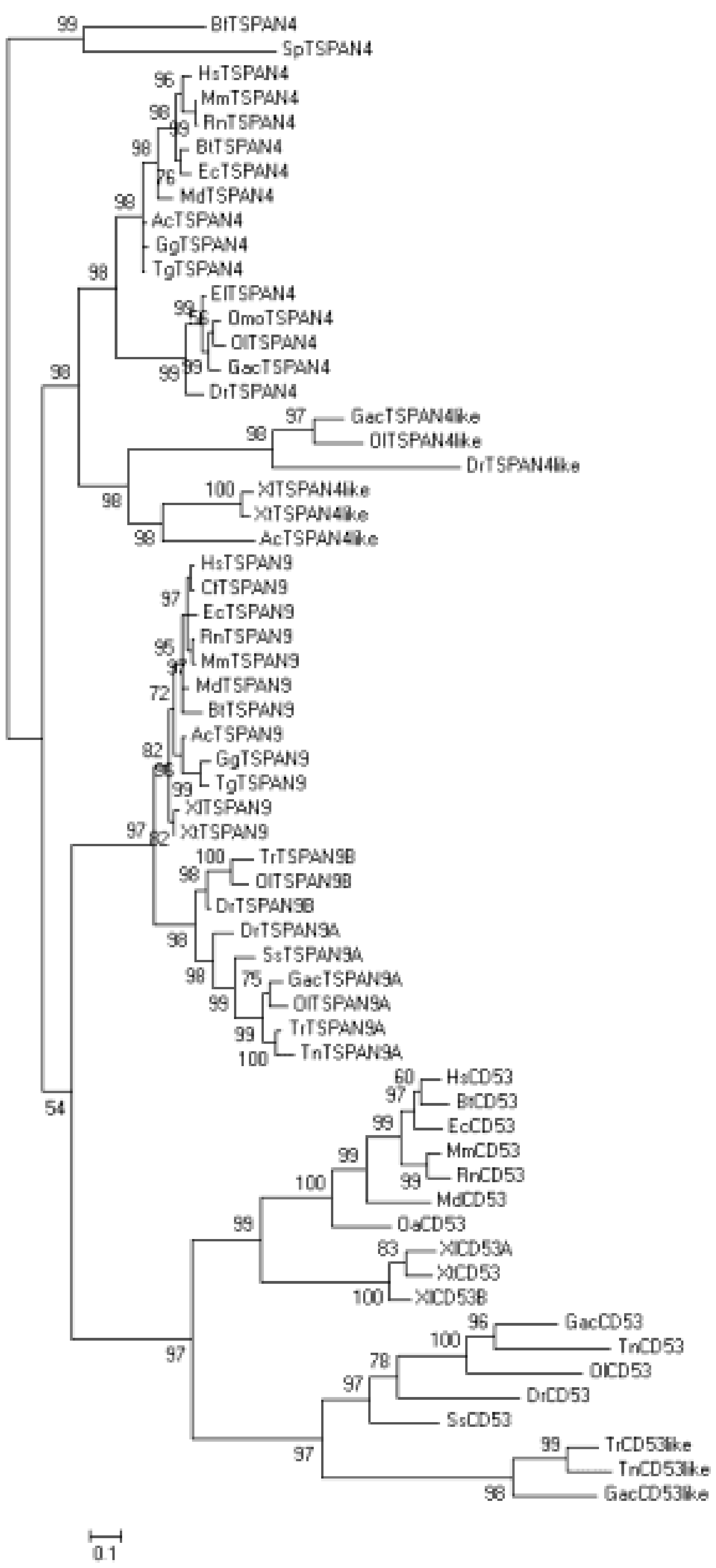

Figure S42. Bayesian protein tree of TSPAN4 family.

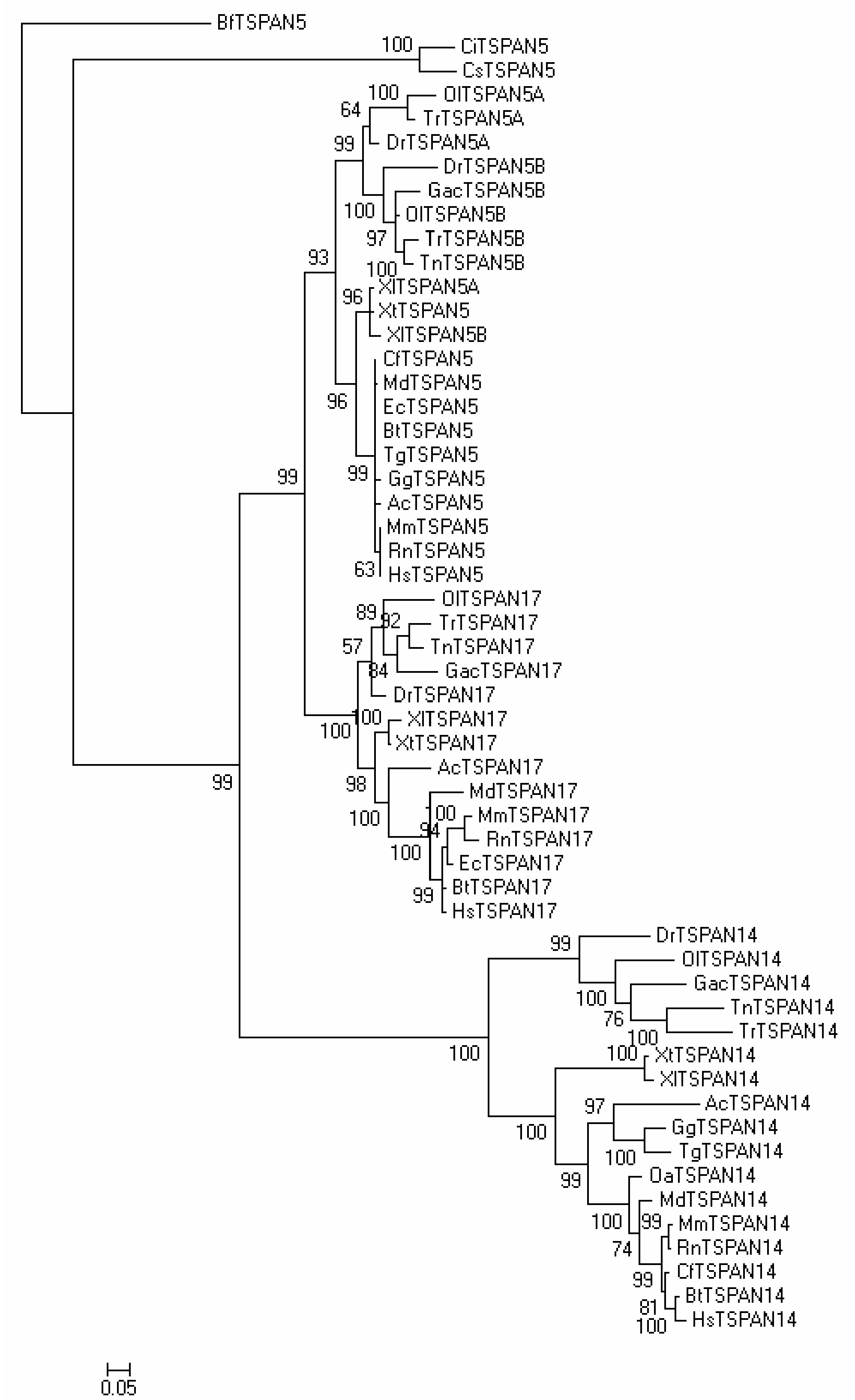

Figure S43. Bayesian protein tree of TSPAN5 family.

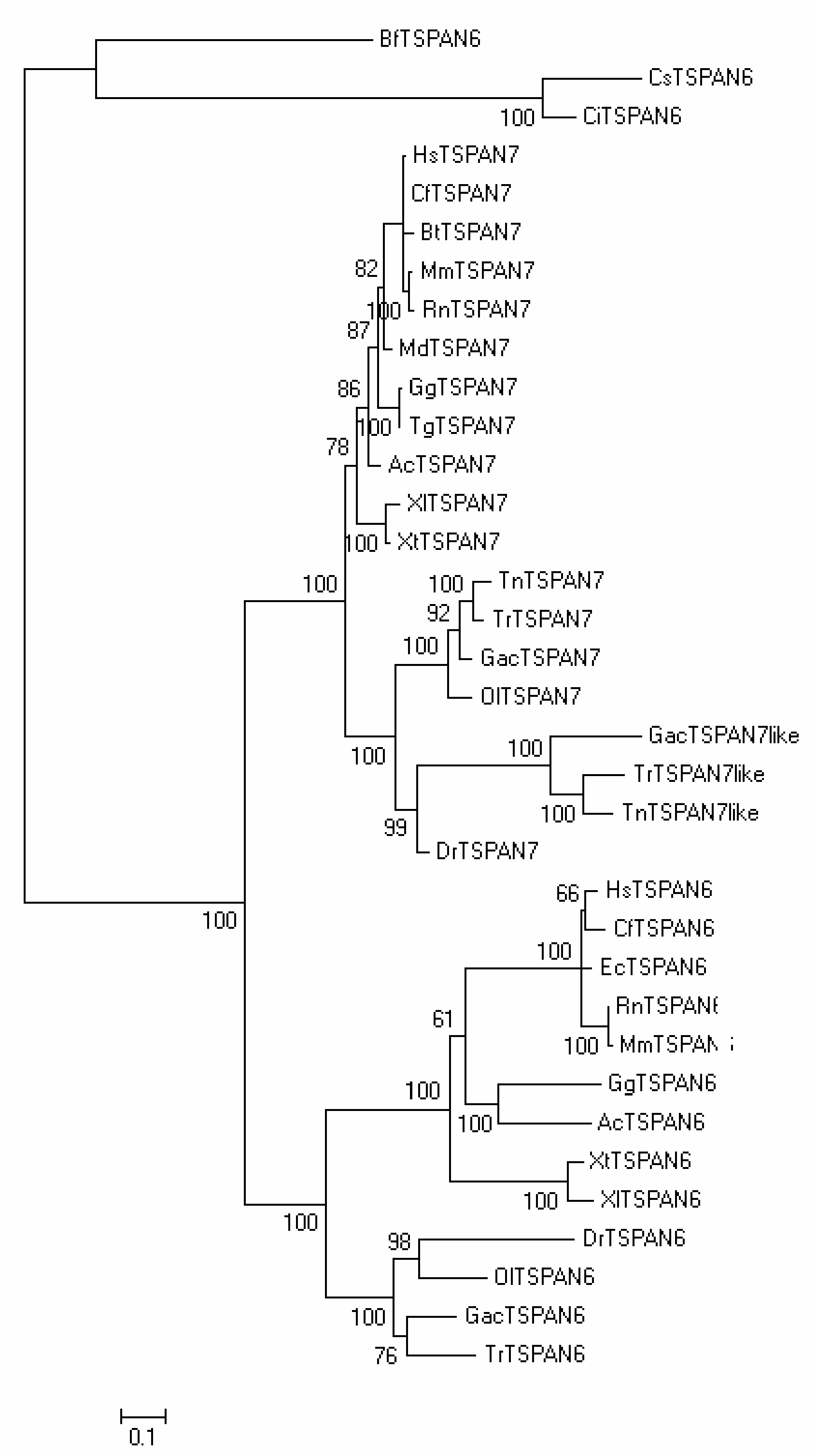

Figure S44. Bayesian protein tree of TSPAN6 family.

Note that lizard(AC) TSPAN7 violates the species tree, but syntenic information suggests that it is the ortholog of mammalian/bird TSPAN7 (MED12-TSPAN7-SRPX-CYBB).

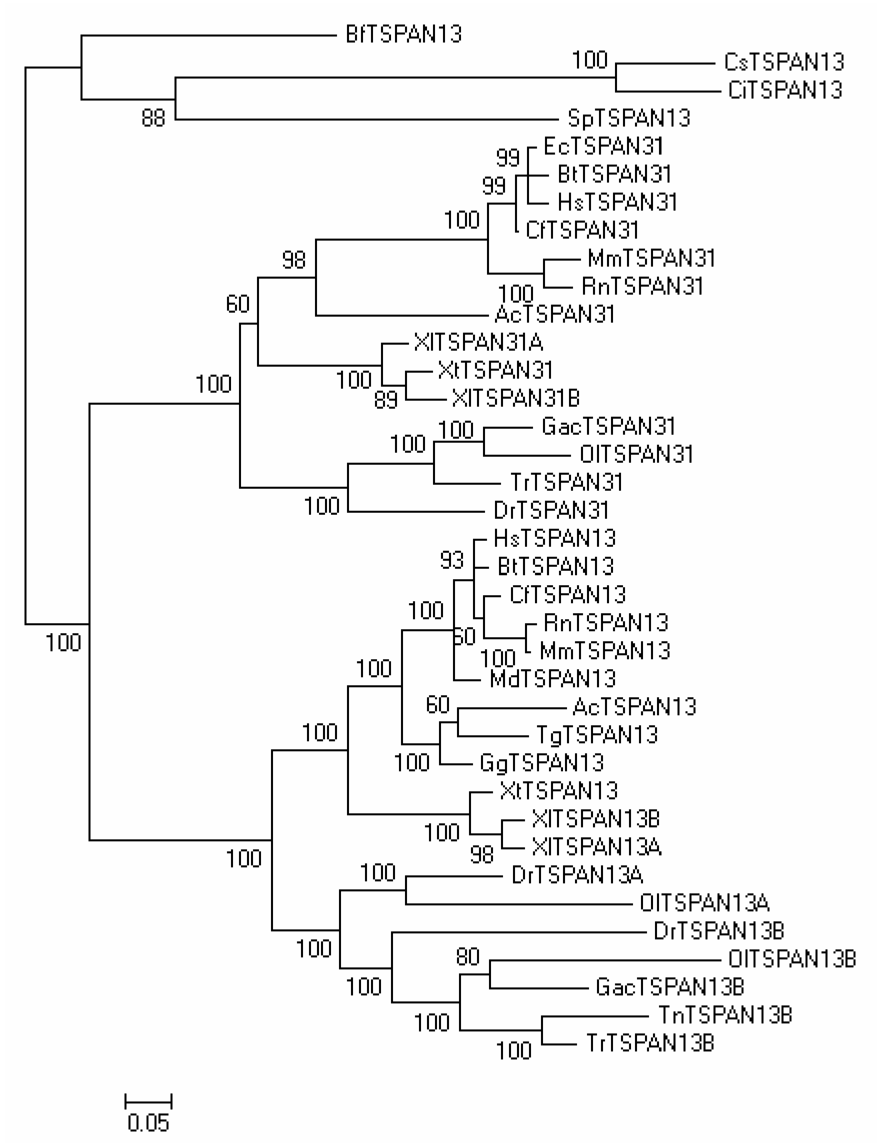

Figure S45. Bayesian protein tree of TSPAN13 family.

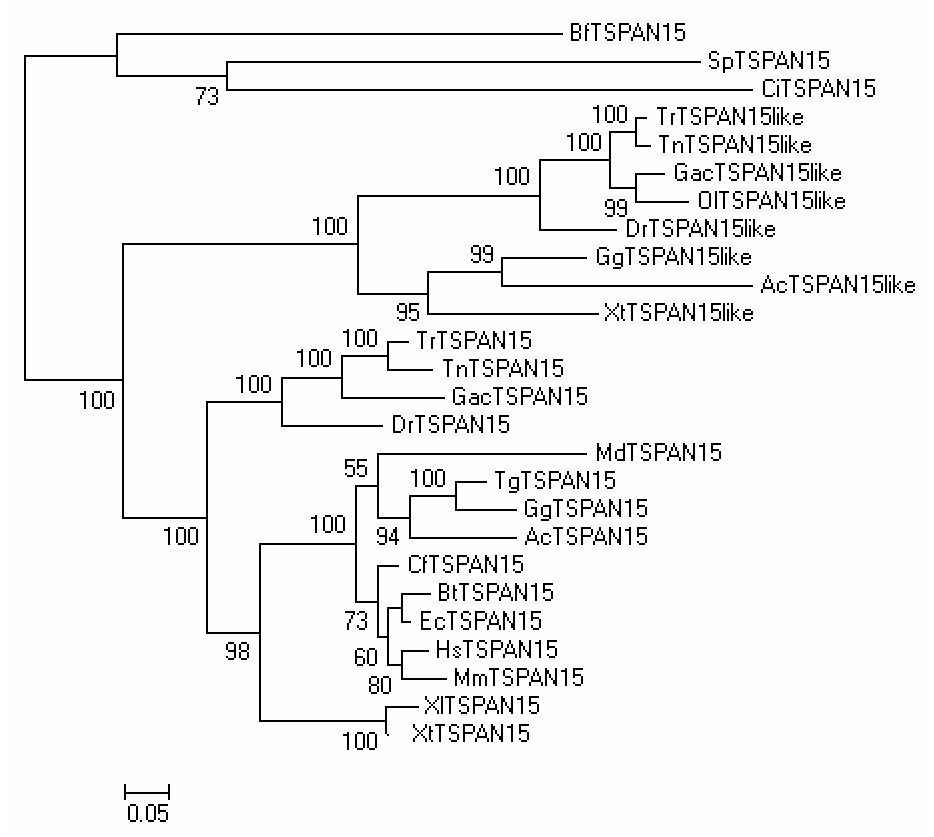

Figure S46. Bayesian protein tree of TSPAN15 family.

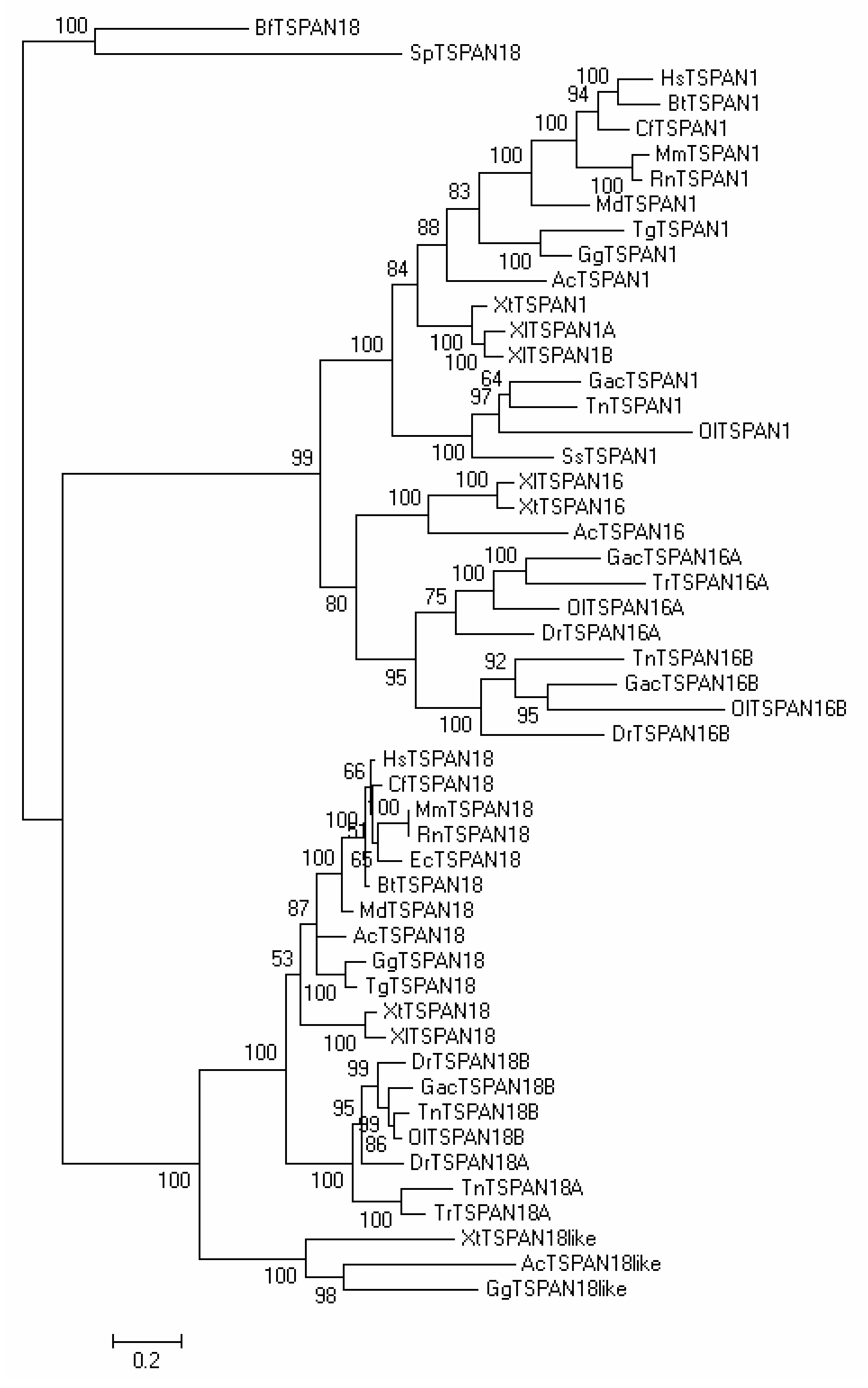

Figure S47. Bayesian protein tree of TSPAN1/TSPAN18 family.

Note that lizard(Ac) TSPAN1 violates the species tree, but syntenic information suggests that it is the ortholog of mammalian/bird TSPAN1 (TSPAN1-POMGNT1).

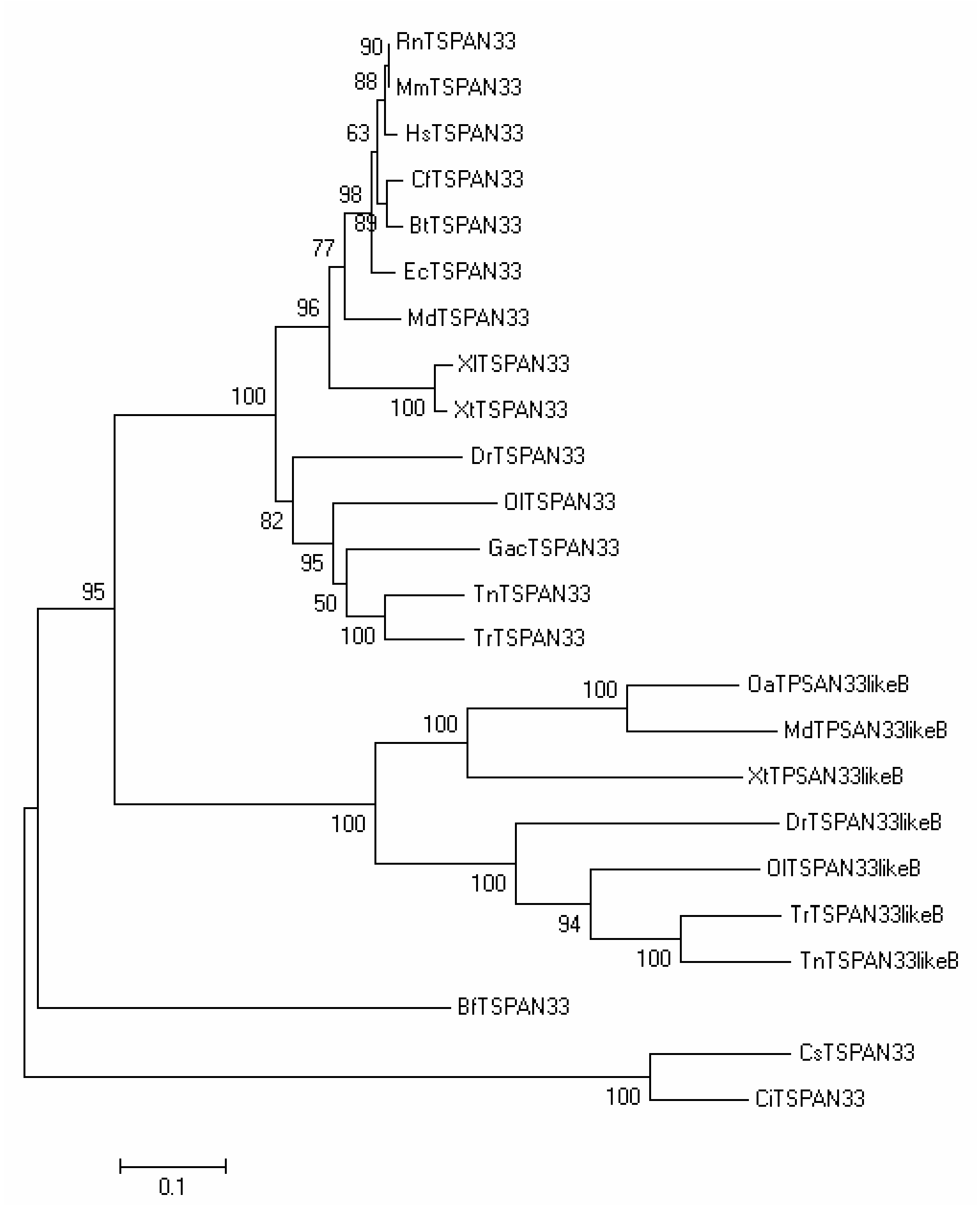

Figure S48. Bayesian protein tree of TSPAN33 family.

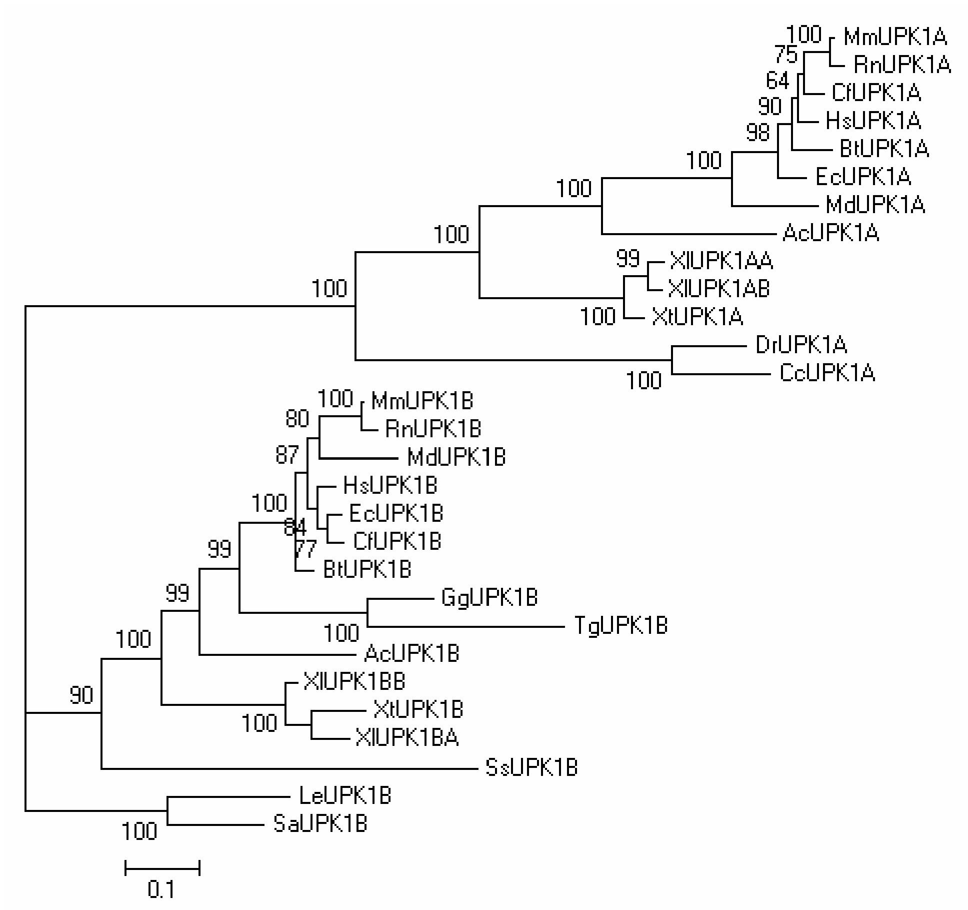

Figure S49. Bayesian protein tree of UPK1 family.

Note that lizard(Ac) UPK1B violates the species tree, but syntenic information suggests that it is the ortholog of bird UPK1B (UPK1B-CDGAP).

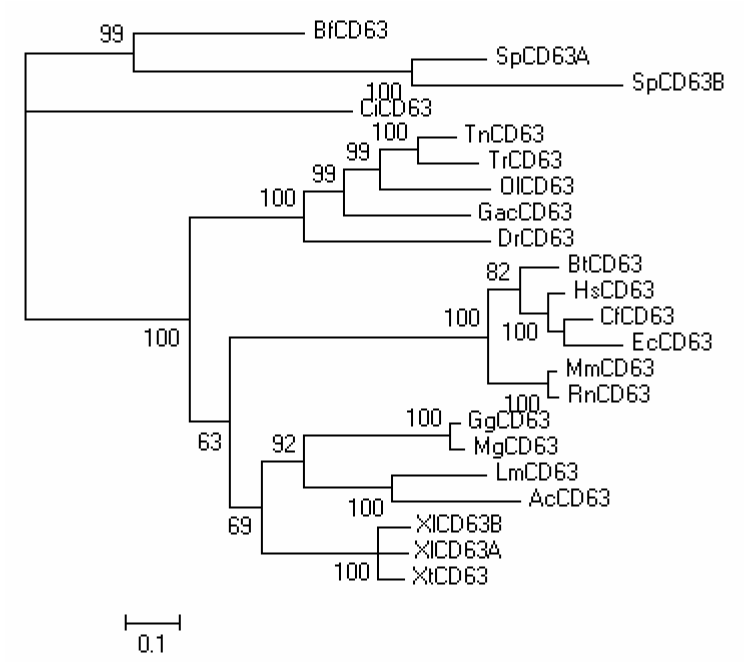

Figure S50. Bayesian protein tree of CD63 family.

Note that mammalian CD63 violates the species tree, but syntenic information suggests that they are the orthologs of reptile/amphibian CD63 (CD63-RDH5-BLOC1S1).

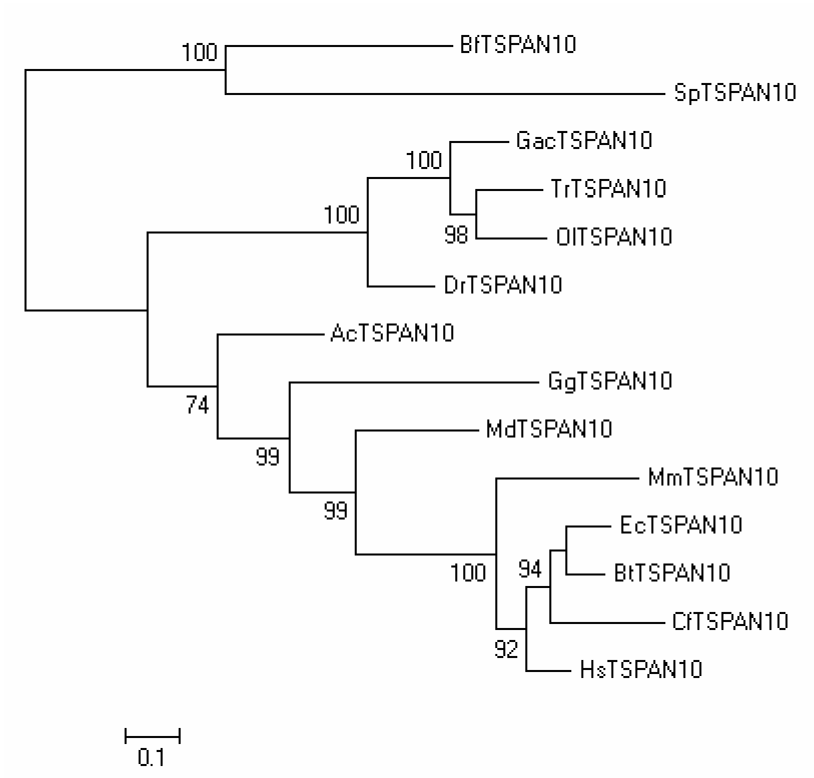

Figure S51. Bayesian protein tree of TSPAN10 family.

Note that lizard (Ac) TSPAN10 violates the species tree, but syntenic information suggests that it is the ortholog of bird TSPAN10 (PDE6G-TSPAN10-NPLOC4).

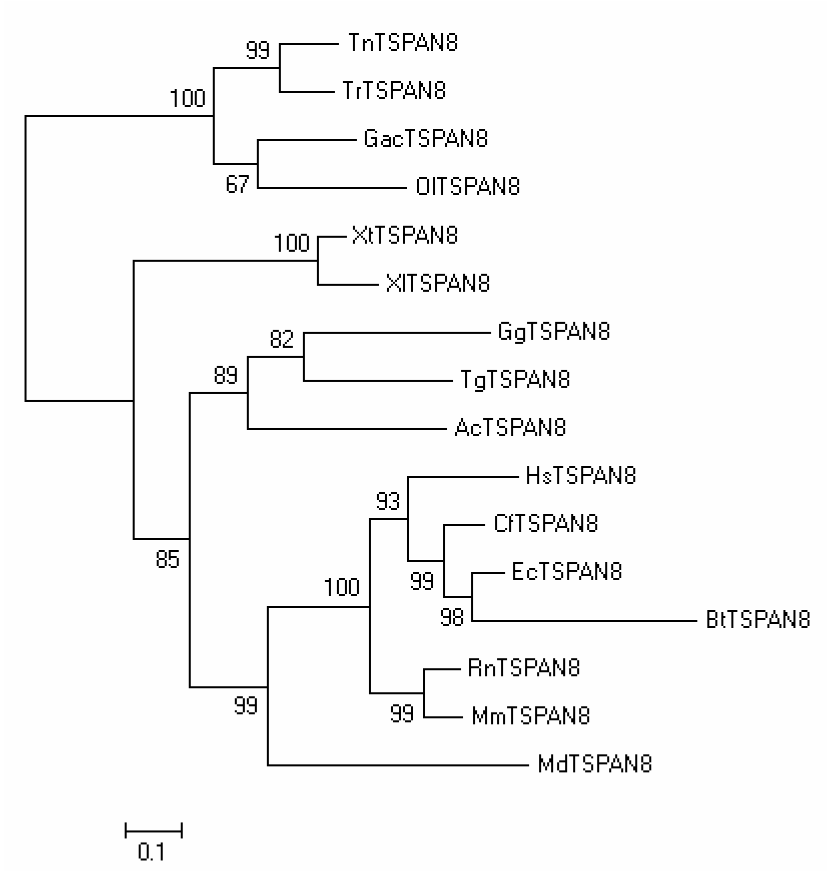

Figure S52. Bayesian protein tree of TSPAN8 family.

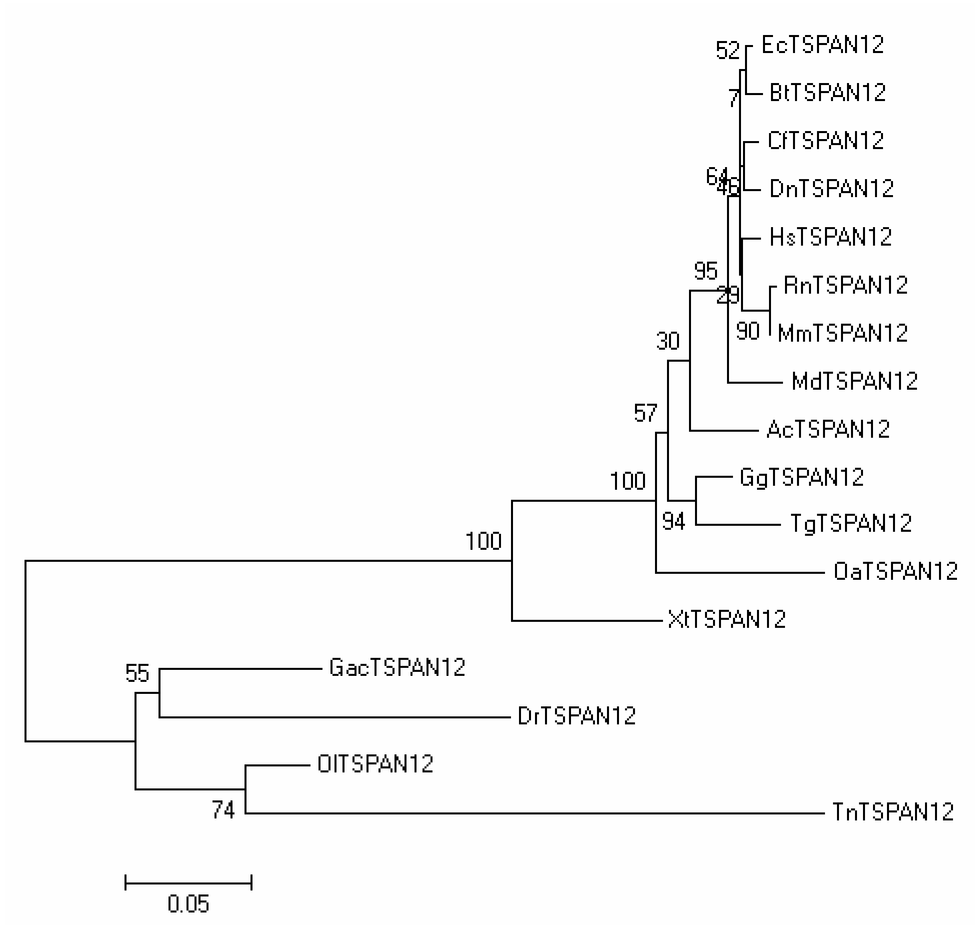

Figure S53. Bayesian protein tree of TSPAN12 family.

Note that platypus and lizard (Ac) TSPAN12 violate the species tree, but syntenic information suggests that they are the orthologs of bird/mammalian TSPAN12 (TSPAN12-ING3-WNT16).
